# Multi-omics analysis of kidney renal cell carcinoma in silico with preliminary *in vivo* validation

**DOI:** 10.3389/fimmu.2026.1732965

**Published:** 2026-06-08

**Authors:** Tingyu Su, Fan Jiang, Jianjun Gao, Xiubin Li, Hongmei Cheng, Yang Wang

**Affiliations:** 1 Department of Nephrology, First Medical Center of Chinese People's Liberation Army (PLA) General Hospital, State Key Laboratory of Kidney Diseases, National Clinical Research Center for Kidney Diseases, Beijing, China; 2 Key Laboratory of Medical Devices and Integrated Traditional Chinese and Western Drug Development for Severe Kidney Diseases, Beijing, China; 3 Key Laboratory of Digital Intelligent TCM for the Prevention and Treatment of Pan-vascular Diseases, Key Disciplines of National Administration of Traditional Chinese Medicine (zyyzdxk-2023310), Beijing, China; 4 Department of Nephrology, the Eighth Medical Center of People's Liberation Army General Hospital, Beijing, China; 5 Section of Health, No. 94804 Unit of the Chinese People’s Liberation Army, Shanghai, China; 6 Resident Standardization Training Cadet Corps, Air Force Medical Center, Beijing, China; 7 Department of Nephrology, the Ninth Medical Center of People's Liberation Army (PLA) General Hospital, Beijing, China; 8 Department of Urology, the Third Medical Center of People's Liberation Army General Hospital, Beijing, China

**Keywords:** bioinformatics, clinical prognosis, immune microenvironment, in silico knockout, kidney renal clear cell carcinoma (KIRC), two-sample mendelian randomization (TSMR)

## Abstract

**Background:**

Kidney renal clear cell carcinoma (KIRC) has a poor clinical prognosis, and its tumor progression and immunotherapy response are closely associated with genomic aberrations and immune microenvironment disorders. This study aimed to identify key prognostic biomarkers and potential therapeutic targets for KIRC via multi-omics integrative analysis and preliminary experimental validation.

**Methods:**

Multi-omics data including transcriptome, methylation, copy number variation (CNV), and immune infiltration data of KIRC were integrated from public databases, and further validated by single-cell RNA sequencing (scRNA-seq), two-sample Mendelian randomization (TSMR), in silico gene knockout, and quantitative PCR (qPCR).

**Results:**

Results demonstrated that CRHBP was a core downregulated prognostic biomarker in KIRC, with stage-dependent low expression and favorable survival predictive value (OS HR = 0.42, 95%CI: 0.395–0.45, P<0.05). ScRNA-seq revealed a compartmentalized CRHBP expression pattern and its differential regulation by immune checkpoint blockade (ICB) treatment and gender. TSMR confirmed the causal risk effect of testicular-derived UCN2 on KIRC, and UCN2 negatively correlated with tumor angiogenesis and hypoxia. Molecular docking screened dabrafenib as a promising candidate drug targeting CRHBP/UCN2 (binding affinity: -11.55/-17.23 kcal/mol). In silico knockout of CRHBP altered SRGN/TYROBP expression and inhibited cytokine production. QPCR verified decreased CRHBP expression in human KIRC tissues and elevated UCN2 expression in mouse KIRC tissues.

**Conclusions:**

The CRHBP-UCN2 axis critically regulates KIRC prognosis and immune microenvironment remodeling. CRHBP serves as a reliable prognostic biomarker and a potential anticancer peptide target, while dabrafenib is a promising therapeutic agent for KIRC.

## 1 Introduction

Kidney renal clear cell carcinoma (KIRC) stands as a formidable adversary in the landscape of urological malignancies, representing the majority of kidney cancer cases (1). Its aggressive nature, coupled with a propensity for metastasis and resistance to conventional therapies, renders KIRC a significant threat to public health (1). The gravity of this condition is further amplified by its often-late diagnosis and the subsequent poor prognosis. The intricate interplay of genomic alterations and immune cell dynamics within the tumor microenvironment is posited as a critical determinant of KIRC progression and patient survival, underscoring the imperative for a deeper understanding of these elements to inform therapeutic strategies (2).

The genomic architecture of KIRC is complex, with numerous studies unveiling a plethora of genetic mutations and copy number variations that contribute to its pathogenesis. Genes such as MFSD4 (3), CRHBP (4), and MPP7 (5) have emerged as pivotal players in the disease’s progression, with their expression patterns reflecting the tumor’s behavior and prognosis. Concurrently, the immune context of KIRC, characterized by the infiltration of various immune cells, including Natural Killer T cells (NKT) (6), FOLR2-Hi expressing tumor-associated macrophages (TAM) (7), and CXCL10-Hi TAM (8), is increasingly recognized for its role in modulating the tumor’s trajectory and response to treatments. Despite these advances, a cohesive framework that integrates genomic and immunological data to predict clinical outcomes in KIRC remains elusive.

Against this backdrop, our study endeavors to bridge this gap by conducting a comprehensive analysis of the genomic and immune cell landscapes in KIRC. By identifying differentially expressed genes (DEGs) and most differentially expressed genes (MDSGs), and elucidating their correlation with immune cell infiltration and clinical prognosis, we aim to discover an eligible biomarker with a significant impact on tumor existence and clinical prognosis. Our findings, which reveal the significant impact of CRHBP on methylation levels and its inverse association with NKT infiltration, offer novel insights into the disease’s biology. Furthermore, the identification of CRHBP’s expression in specific immune cell types and its modulation by immune checkpoint inhibitors (ICB) and sex provides a foundation for the development of tailored therapeutic interventions. This study is instrumental in advancing our understanding of KIRC, with the potential to refine prognostic assessments and guide personalized medicine approaches for patients. No artificial intelligence was used in the research and writing of this paper, adhering to the TITAN guidelines 2025 (9).

## 2 Materials and methods

The work has been reported in line with the REMARK criteria (10).

### 2.1 Screening of prognostic biomarkers

**Figure 1:**
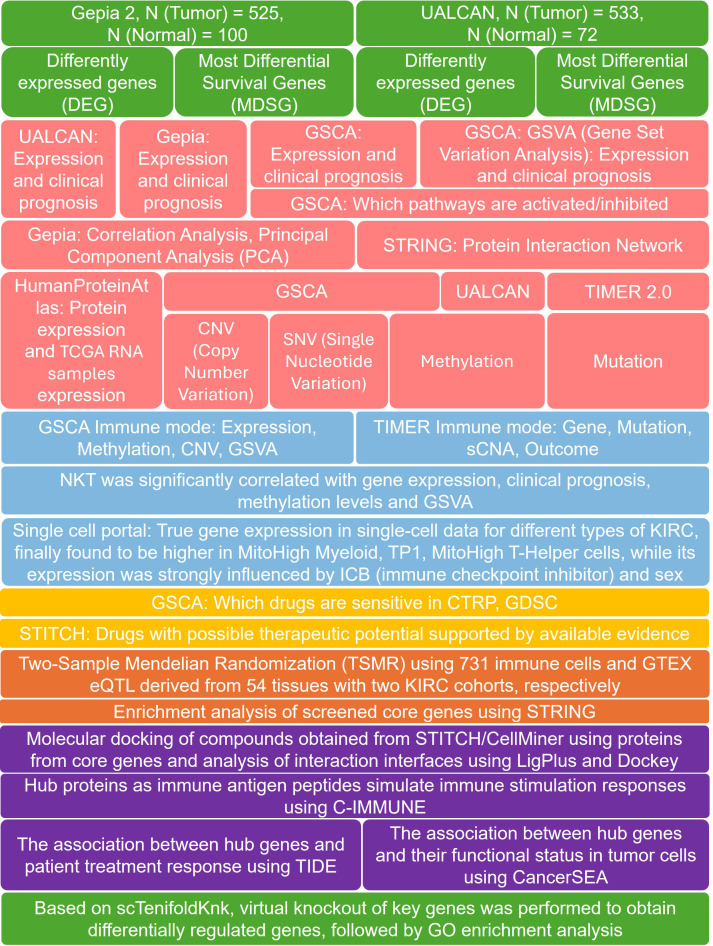
Research roadmap.

The overall research workflow is illustrated in **Figure 1**. GEPIA2 database was used to screen DEGs and major prognostic differentially expressed genes (MSDGs) of KIRC (Tumor=525, Normal=100) with screening criteria: Log2FC ≥ 1, FDR-adjusted P-value (q-value) = 0.05, LIMMA differential analysis (11). UALCAN database was used to obtain KIRC DEGs and prognostic MSDGs based on TCGA dataset (Tumor=533, Normal=72) (12). The Upset tool of Chiplot online platform was applied to acquire the intersection of DEGs and MSDGs from the two databases, and overlapping genes were identified as candidate biomarkers.

### 2.2 Multi-omics correlation analysis of candidate biomarkers

GEPIA2, UALCAN and GSCA databases were comprehensively utilized to analyze the transcriptome expression, stage-specific expression, and prognostic value of candidate biomarkers in KIRC (13, 14). The promoter methylation levels, single nucleotide variation (SNV), and copy number variation (CNV) of candidate genes were analyzed via GSCA and MethHC 2.0 databases to eliminate database deviation (15–19).

For immune infiltration analysis, TIMER2.0 and GSCA immune modules were used to evaluate the correlation between gene expression/genetic variation and immune cell abundance, and multi-algorithm immune infiltration results were summarized and visualized via SCImago Graphica software (20).

### 2.3 Single-cell RNA sequencing validation

Single-cell transcriptome data of advanced KIRC immunotherapy (SCP1288 dataset, 22879 cells) were downloaded from the Broad Single Cell Portal for validation (21, 22). Subgroup analyses were performed based on cell lineage, ICB treatment status, and gender. Key immune cell subtypes including MitoHigh Myeloid, MitoHigh T-Helper cells, VSIR-Hi TAMs and CXCL10-Hi TAMs were focused on to analyze the specific expression characteristics of candidate genes in different tumor microenvironment cell subsets.

MitoHigh Myeloid & MitoHigh T-Helper Cells: Definition: “MitoHigh”: Indicates cells with high mitochondrial metabolic activity, often linked to enhanced oxidative phosphorylation (OXPHOS). This metabolic state influences cell function, survival, and differentiation. Myeloid Cells: Include macrophages, dendritic cells, and monocytes. “MitoHigh Myeloid” specifically describes a subset of tumor-associated myeloid cells (e.g., macrophages) with elevated mitochondrial respiration. T-Helper (Th) Cells: CD4^+^ T cells that coordinate immune responses. “MitoHigh T-Helper” refers to a Th subset with high mitochondrial activity, often associated with sustained effector functions or specific differentiation states (e.g., Th1/Th17). Functional Significance: These cells exhibit heightened energy production, supporting prolonged immune activity in the tumor microenvironment (TME). In ICB (Immune Checkpoint Blockade) contexts, their metabolic state may correlate with therapy responsiveness. For example, MitoHigh CD8^+^ T cells show enhanced persistence in tumors post-ICB (23, 24).

Tumor program 1 (TP1) Cells: Definition: A subpopulation of tumor cells identified in renal cell carcinoma (RCC) scRNA-seq data via gene expression and copy number alterations (25, 26).

VSIR-Hi TAMs & CXCL10-Hi TAMs: Definition: TAMs (Tumor-Associated Macrophages): Macrophages infiltrating tumors, polarized into distinct functional states. VSIR-Hi TAMs: Express high levels of V-Set Immunoregulatory Receptor (VSIR, also called VISTA), an immune checkpoint molecule that suppresses T-cell activity. These TAMs display a hybrid state (mixed M1/M2 features) with co-expression of both pro-inflammatory and immunosuppressive markers. CXCL10-Hi TAMs: Characterized by high secretion of C-X-C Motif Chemokine Ligand 10 (CXCL10), a chemoattractant for T cells and NK cells. These are pro-inflammatory (M1-like) TAMs that enhance antitumor immunity. ICB Modulation: VSIR-Hi TAMs expand in ICB-resistant patients, inhibiting T-cell function via VSIR. CXCL10-Hi TAMs correlate with better ICB outcomes by recruiting effector T cells (27, 28).

### 2.4 Drug sensitivity screening and molecular docking

GSCA drug module (GDSC and CTRP databases) was used to screen gene-related sensitive drugs, and the intersection of candidate drugs was obtained via Jvenn online tool. STITCH database was used to analyze gene-drug interaction networks (29, 30). FDA-approved and clinical trial drugs with significant correlation with target genes were further screened via CellMiner database (31, 32). The 3D structures of CRHBP (Alphafold ID: P24387) and UCN2 (PDB ID: 3N95) were obtained. AutoDock Vina 1.2.7 and Dokey v1.0.3 were used for molecular docking. LigPlus was applied for post-docking interaction analysis. Compounds with binding affinity < -5 kcal/mol were defined as well-binding candidate drugs, and toxic drugs were excluded based on published literature (33, 34).

### 2.5 Two-sample mendelian randomization analysis

Peripheral blood immune cell GWAS data (PMID: 32929287) were used as exposure factors, and two KIRC-related phenotypes from FinnGen database were set as outcome factors (35). The TwoSampleMR R package was used for TSMR analysis with standard parameters (clump_kb=10000, clump_r2 = 0.001, P<5×10, F-statistic>10). MR-Egger intercept test and MR-PRESSO global test were performed to detect horizontal pleiotropy. Benjamini-Hochberg (BH) and Bonferroni multiple testing corrections were applied to control false positive rate. PPI network and GO enrichment analysis of causal genes were performed via STRING database.

### 2.6 Immune simulation and treatment response analysis

C-IMMSIM online server was used for in silico immune stimulation simulation of CRHBP and UCN2 epitope peptides, with three simulated immunizations on days 1, 52 and 100 (36–42). TIDE database was used to analyze the correlation between hub genes and ICB treatment response, and Kaplan-Meier survival curves were plotted to evaluate prognostic differences of ICB treatment subgroups. CancerSEA database was applied to analyze the correlation between hub genes and KIRC tumor functional status (43).

### 2.7 In silico gene knockout analysis

This study utilized single-cell transcriptomic data from the GSE152938 dataset for renal cell carcinoma. After manually annotating the data in conjunction with literature sources and comparing the cellular composition ratios between ccRCC and normal tissues, only ccRCC tumor cells were retained for subsequent analysis. The raw count matrix of RNA expression was extracted, and the top 3000 highly variable genes were screened, with the target gene CRHBP forcibly included. To reduce computational cost, a total of 500 tumor cells were randomly sampled to construct the final analytical matrix.

The scTenifoldKnk algorithm was applied to perform in silico knockout of CRHBP, and gene regulatory networks were constructed to identify genes with expression perturbation after gene knockout (44). Genes with adjusted p < 0.05 were defined as significantly differentially regulated genes.

Functional enrichment analysis of Gene Ontology (GO), including biological process (BP), cellular component (CC), and molecular function (MF), was performed using the clusterProfiler package. The false discovery rate (FDR) was used for multiple testing correction. Volcano plot, top 20 significant gene bar plot, as well as GO enrichment dot plot and bar plot were generated for result visualization.

### 2.8 Quantitative RT-PCR validation

Total RNA was isolated from all samples using TRIzol reagent (Invitrogen, Carlsbad, CA) in strict accordance with the manufacturer’s protocol. mRNA quantification was performed via RT-qPCR on an Applied Biosystems 7500 detection system (Applied Biosystems, Foster City, CA), with target-specific primer sequences provided in **Supplementary Material 1**. Primary murine renal tubular epithelial cells were isolated using established methodologies (45). The human renal proximal tubular cell line HK2 (Wuhan Procell Biotechnology Co., Ltd., Wuhan, China) and human renal carcinoma cell line A-498 (same supplier) were maintained in Minimum Essential Medium (MEM) supplemented with 10% fetal bovine serum (FBS). The murine renal carcinoma cell line Renca (Wuhan Procell Biotechnology Co., Ltd.) was cultured in RPMI-1640 medium containing 10% FBS, 0.1 mM non-essential amino acids, 1 mM sodium pyruvate, and 2 mM L-glutamine. All experimental samples underwent duplicate RT-qPCR analyses. Resultant data were imported into the R studio (2025.05.0) and R statistical environment (v4.5.0), where batch effect correction and normalization were implemented using the limma package (v3.64.3). Subsequent between-group comparisons employed Wilcoxon rank-sum tests with Benjamini-Hochberg (BH) false discovery rate adjustment for multiple hypothesis correction.

## 3 Results

### 3.1 MFSD4, CRHBP, MPP7 is both DEG and MDSG

**Figure 2:**
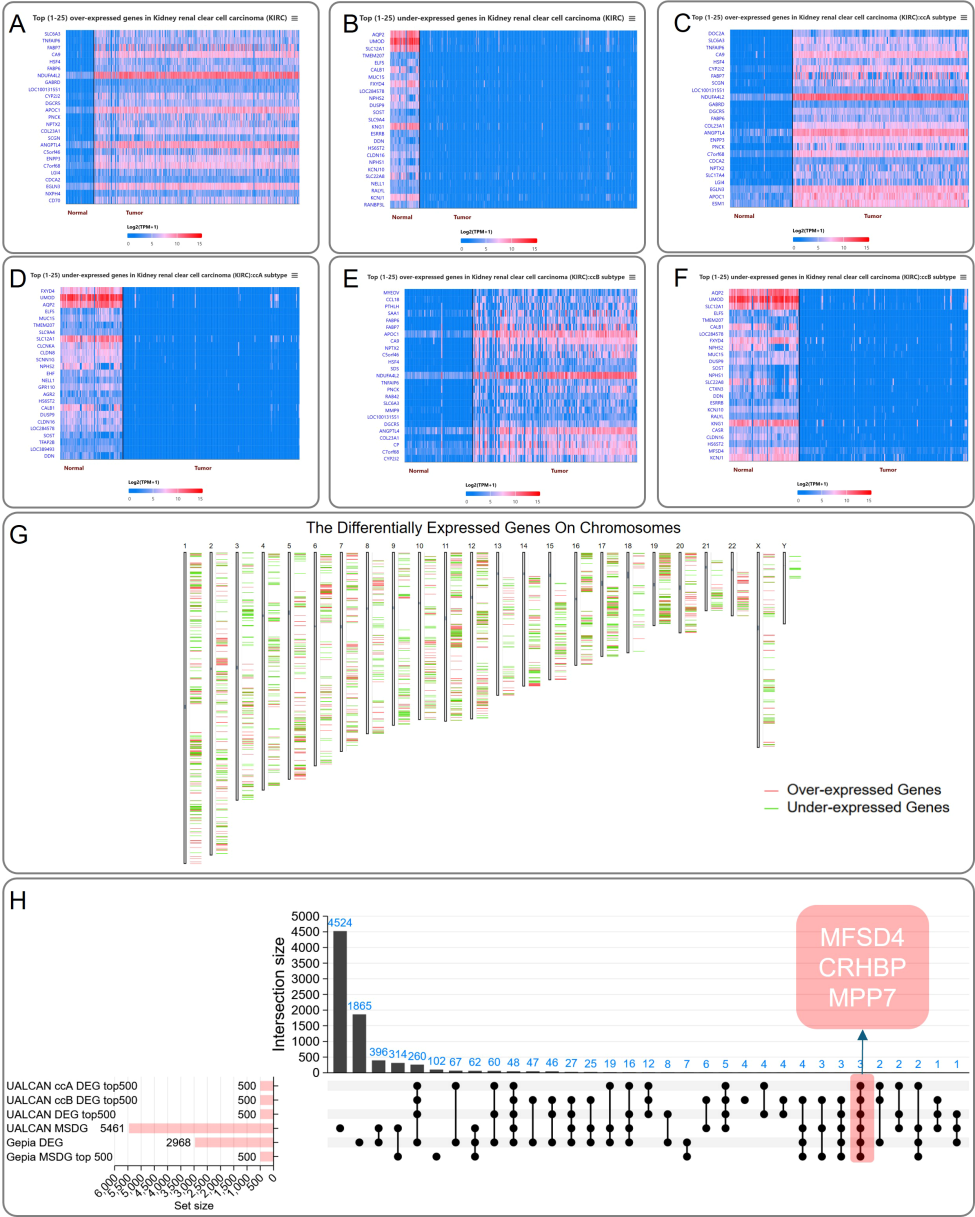
DEG expression and MDSG intersection result in both databases. **(A–F)** Heatmap of the top 25 differentially expressed genes in the UALCAN database; gene expression levels are normalized to Log2(TPM + 1). The x-axis represents the sample, and the y-axis represents the gene name. **(A)** Heatmap of the top 25 DEG of high expression of KIRC in UALCAN. **(B)** Heatmap of the top 25 DEGs of low expression of KIRC in UALCAN. **(C)** Heatmap of the top 25 DEG of high expression of ccA KIRC in UALCAN. **(D)** Heatmap of the top 25 DEG of low expression of ccA KIRC in UALCAN. **(E)** Heatmap of the top 25 DEG of high expression of ccB KIRC in UALCAN. **(F)** Heatmap of the top 25 DEG of low expression of ccB KIRC in UALCAN. **(G)** Expression of Gepia DEG on chromosomes, red indicates overexpression and green indicates low expression. Each row represents one chromosome. **(H)** Upset visualization of the intersection results of the six datasets, three biomarkers appear in all six datasets: MFSD4, CRHBP, and MPP7.

Gepia obtained 2970 DEGs and the top 500 MDSGs; UALCAN obtained 500 top DEGs in KIRC/ccA KIRC/ccB KIRC, and 5461 MDSGs (original results in **Supplementary Table 1**). In UALCAN, the top 25 differentially up-regulated and down-regulated genes in KIRC were **Figures 2A, B**, the top 25 differentially up-regulated and down-regulated genes in KIRC ccA subtype were **Figures 2C, D**, the top 25 differentially up-regulated and down-regulated genes in KIRC ccB subtype were **Figures 2E, F**, and the overexpression and underexpression of Gepia DEG on chromosomes are shown in **Figure 2G**. The six parts of the Upset diagram intersection result in **Figure 2H**, the intersection of 3 genes named MFSD4, CRHBP, and MPP7. It is important to note that MFSD4 is not recorded in some databases because both MFSD4A and MFSD4 are members of the Major Facilitator Superfamily (MFS) of transporter protein families and MFSD4A is partially representative of MFSD4, so MFSD4A was used for alternative analyses when necessary.

### 3.2 MFSD4, CRHBP, MPP7 on clinical prognosis and expression in different tissue types

**Figure 3:**
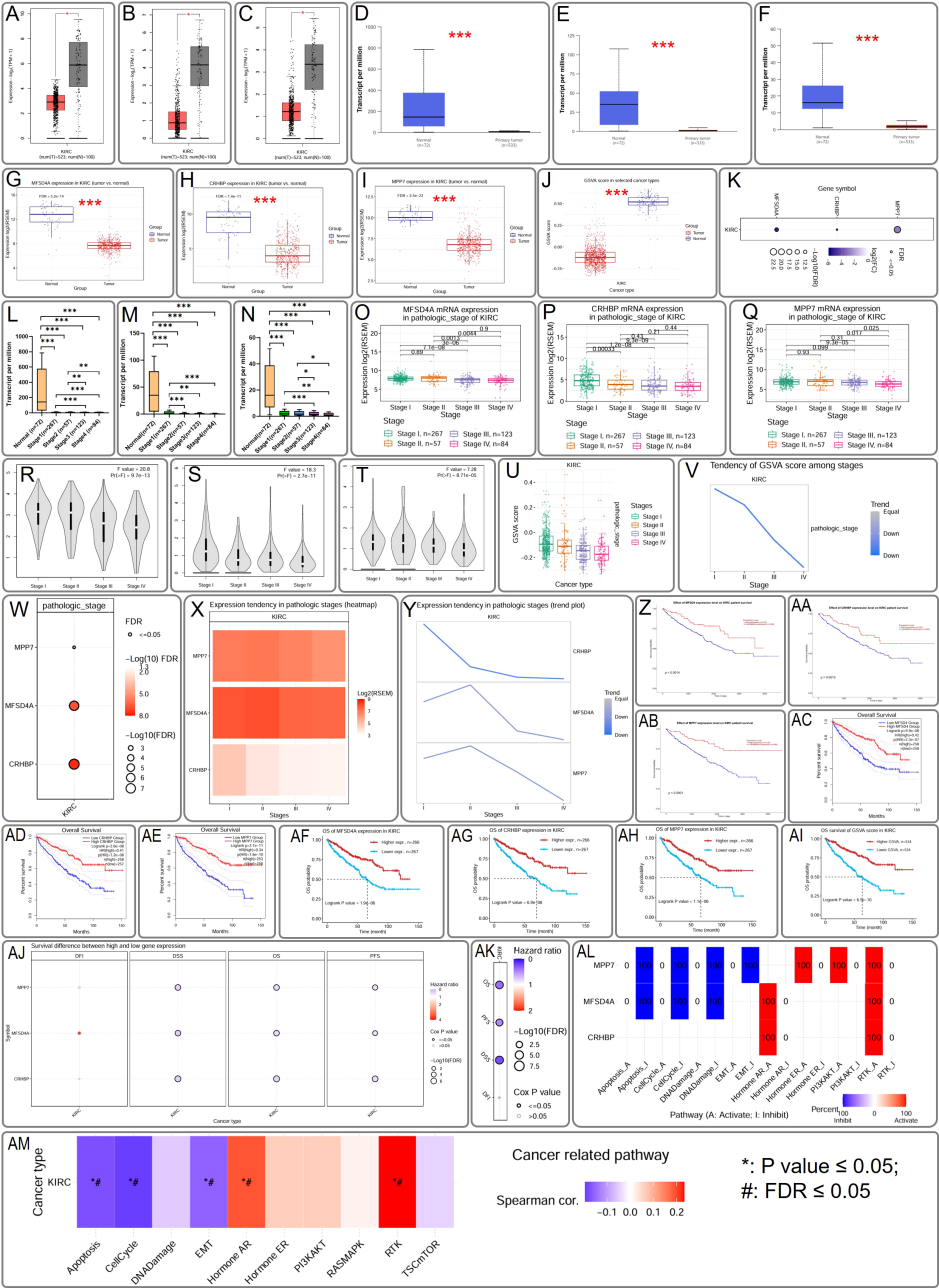
Impact of MFSD4, CRHBP, and MPP7 in Gepia and UALCAN on clinical prognosis and expression in different tissue types. MFSD4 **(A)**, CRHBP **(B)**, MPP7 **(C)** mRNA expression in tumor and normal tissues in Gepia. MFSD4 **(D)**, CRHBP **(E)**, MPP7 **(F)** mRNA expression in tumor and normal tissues in UALCAN. MFSD4 **(G)**, CRHBP **(H)**, MPP7 **(I)**, GSVA **(J)** mRNA expression in tumor and normal tissues in GSCA. **(K)** Some basic parameters of the differential expression of MFSD4, CRHB, and MPP7 in GSCA in tumor and normal tissues. MFSD4 **(L)**, CRHBP **(M)**, MPP7 **(N)** mRNA expression in tumor and normal tissues at different stages in UALCAN. MFSD4 **(O)**, CRHBP **(P)**, MPP7 **(Q)** mRNA expression in tumor and normal tissues at different stages in GSCA. MFSD4 **(R)**, CRHBP **(S)**, MPP7 **(T)** mRNA expression in tumor and normal tissues at different stages in Gepia. The size of the GSVA score in tumor tissues at different stages **(U)**, and the change of GSVA score at different stages **(V)**. Some basic parameters of the differential expression of MFSD4, CRHB, and MPP7, in GSCA at different pathological stages **(W)**, heatmap of the expression trend **(X)**, line graph of the expression trend **(Y)**. The effect of mRNA expression of MFSD4 **(Z)**, CRHBP **(AA)**, and MPP7 **(AB)** in UALCAN on the prognosis [OS (Overall Survival)]. The effect of mRNA expression of MFSD4 **(AC)**, CRHBP **(AD)**, and MPP7 **(AE)** in Gepia on the prognosis (OS). Effects of high and low mRNA expression of MFSD4 **(AF)**, CRHBP **(AG)**, MPP7 **(AH)**, and GSVA score **(AI)** on prognosis (OS) in GSCA. Effect of high and low mRNA expression of MFSD4, CRHBP, MPP7 in GSCA on prognosis [DFI (Disease-Free Interval), DSS (Disease-Specific Survival), OS, PFS (Progression-Free Survival)] **(AJ)**. Effect of GSVA score on prognosis (DFI, DS, OS, PFS) **(AK)**. MFSD4, CRHBP, and MPP7 in GSCA in KIRC for pathway regulation, A indicates activation, and I indicates inhibition **(AL)**. GSVA score in KIRC for pathway regulation, A indicates activation, and I indicates inhibition **(AM)**.

All three databases, Gepia, UALCAN, and GSCA, show that MFSD4, CRHBP, and MPP7 are under-expressed in KIRC (**Figures 3A–K**), with a gradual decrease from stage 1 to 4 (**Figures 3L–Y**), and have a significant impact on prognosis (**Figures 3Z–AK**. **Figures 3Z–AB** based on UALCAN. HR (High MFSD4 Group (n=133) vs Low/Medium MFSD4 Group (n=398)) = Unreported, P value = 0.0014 in **Figure 3Z**. HR (High CRHBP Group (n=132) vs Low/Medium CRHBP Group (n=399)) = Unreported, P value = 0.0015 in **Figure 3AA**. HR (High MPP7 Group (n=133) vs Low/Medium MPP7 Group (n=398)) = Unreported, P value < 0.0001 in **Figure 3AB**. **Figures 3AC–AE** based on Gepia 2. OS (Overall Survival) HR (High MFSD4 Group (n=258) vs Low MFSD4 Group (n=258)) = 0.42, P value = 2.3e-07 in **Figure 3AC**. OS HR (High CRHBP Group (n=258) vs Low CRHBP Group (n=257)) = 0.41, P value = 7.2e-08 in **Figure 3AD**. OS HR (High MPP7 Group (n=253) vs Low MPP7 Group (n=256)) = 0.34, P value = 1.6e-10 in **Figure 3AE**. **Figures 3AF–AI** based on GSCA. OS HR (High MFSD4A Group (n=266) vs Low MFSD4 Group (n=267)) = 0.48, P value = 1.9e-06 in **Figure 3AF**. OS HR (High CRHBP Group (n=266) vs Low CRHBP Group (n=267)) = 0.43, P value = 6.9e-08 in **Figure 3AG**. OS HR (High MPP7 Group (n=266) vs Low MPP7 Group (n=267)) = 0.47, P value = 1.1e-06 in **Figure 3AH**. **Figure 3AI** based on GSCA GSVA. OS HR (High GSVA Group (n=534) vs Low GSVA Group (n=534)) = 0.38, P value = 6.5e-10). UALCAN, Gepia2, and GSCA together indicated HR of 0.42, 0.41, 0.34, 0.48, 0.43, 0.47, 0.38, i.e., an interquartile range of HR = 0.42 [0.395, 0.45] (**Figures 3J–K**). All three genes acted as activators of the RTK (receptor tyrosine kinase) pathway (**Figure 3AL**), and the GSVA of the three genes showed inhibitory effects on three pathways, Apoptosis, CellCycle, and EMT, and activation of the Hormone AR, RTK pathway (**Figure 3AM**).

### 3.3 Real-world expression, CNV, Methylation, and prognosis

**Figure 4:**
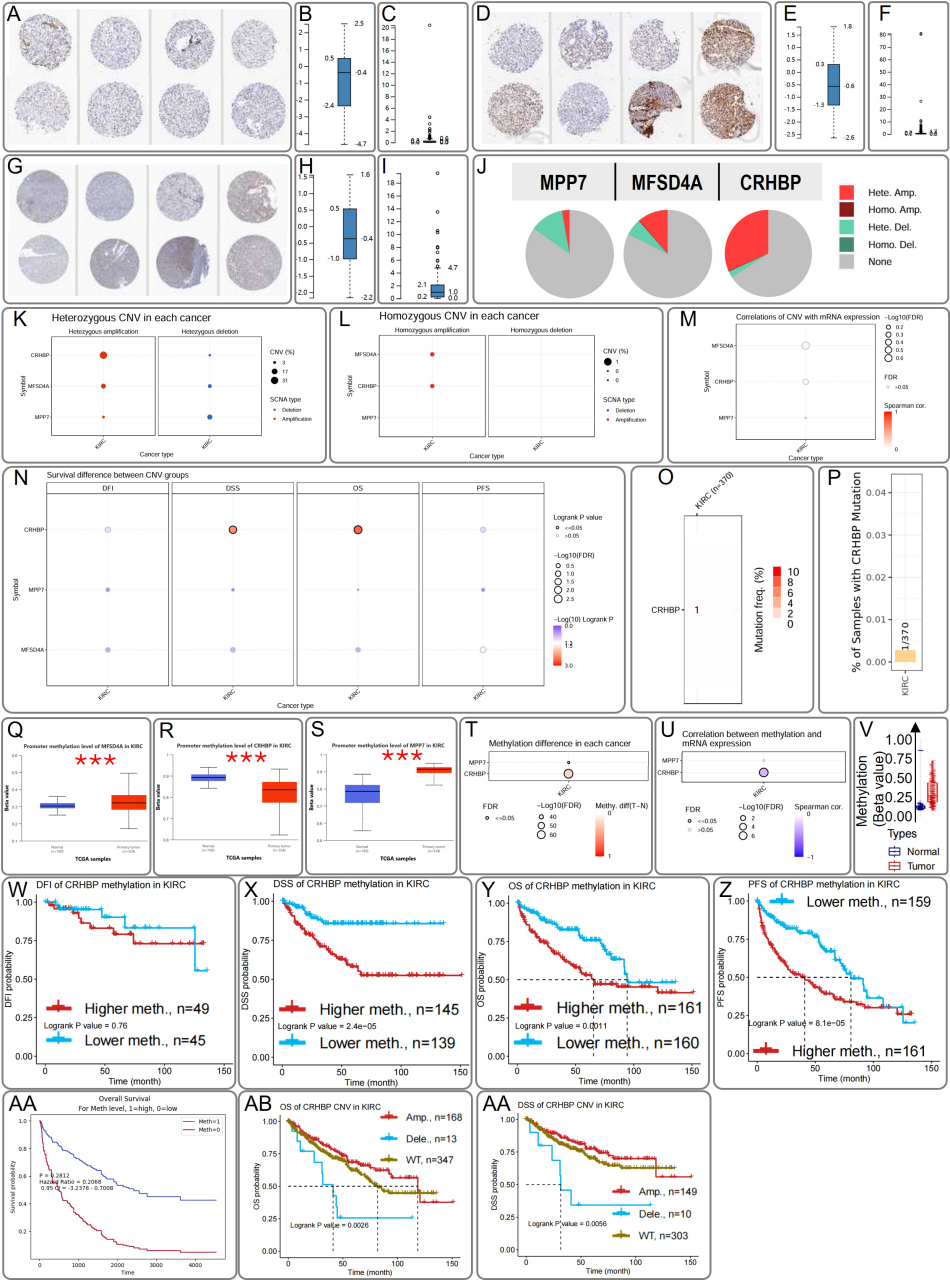
Multidimensional data for three biomarkers. Immunohistochemical results of MFSD4 in Kidney Cancer Adenocarcinoma NOS **(A)**, true protein expression of MFSD4 **(B)**, true mRNA expression of MFSD4 **(C)**. Immunohistochemical results of CRHBP **(D)**, true protein expression **(E)**, and true mRNA expression **(F)**. Immunohistochemical results of MPP7 **(G)**, true protein expression of MPP7 **(H)**, and true mRNA expression of MPP7 **(I)**. Pie charts of the percentage of the number of samples for the five cases of CNV (Copy Number Variation) of the three biomarkers **(J)**. Heterozygous CNV of three biomarkers in KIRC **(K)**. Homozygous CNV of three biomarkers in KIRC **(L)**. Correlation between CNV and mRNA expression of the three biomarkers **(M)**. Differences between the four prognostic indicators of the CNV groups of the three biomarkers **(N)**. Mutation probability of the three biomarkers in the GSCA database **(O)**. Mutation probability **(P)** for the three biomarkers in the TIMER database. MFSD4 **(Q)**, CRHBP **(R)**, MPP7 **(S)** promoter methylation levels. Differential methylation of CRHBP and MPP7 in KIRC tissue and normal tissue **(T)**. Correlation between methylation level of CRHBP, MPP7, and mRNA expression **(U)**. Comparison of methylation levels of CRHBP, red in KIRC and blue in Normal tissue **(V)**. Kaplan-Meier curves depicting the association of CRHBP methylation levels with clinical outcomes in KIRC, including disease-free interval (DFI, **W**), disease-specific survival (DSS, **X**), overall survival (OS, **Y**), and progression-free survival (PFS, **Z**). **(AA)** MethHC 2.0 database validated the impact of CRHBP methylation levels on KIRC prognosis. Red indicates low methylation levels, and blue indicates high methylation levels.

The real-world expression of MPP7 proteins in **Figures 4A, B**, mRNAs in **Figure 4C**; for MFSD4A proteins in **Figures 4D, E**, mRNAs in **Figure 4F**; for CRHBP proteins in **Figures 4G–H**, mRNAs in **Figure 4I**. The protein content: CRHBP (0.4, [-1.0, 0.5]) > MPP7 (0.4, [-2.4, 0.5]) > MFSD4A (-0.6, [-1.3, -0.6]) can be found. Meanwhile, the same trend was observed for mRNA (CRHBP (1.0, [0.2, 2.1]) > MPP7 (0.7, [0.0, 1.7]) > MFSD4A (0.2, [0.0, 0.6]). Full, uncropped images can be found in **Supplementary Material 2**). The proportions of various CNV changes for the three genes in the KIRC samples documented in the GSCA database are shown in **Figure 4J**. The proportion of Hetezygous amplification was CRHBP > MFSD4A > MPP7 (**Figure 4K**), the proportion of Hetezygous deletion was MPP7 > MFSD4A > CRHBP (**Figure 4K**), and the proportion of Homozygous amplification and Homozygous deletion was both minimal (**Figure 4L**). The correlation of all three genes with CNV showed FDR ≥ 0.05 (**Figure 4M**), and the CNV group with CRHBP reflected significant differences in the prognostic indicators DSS and OS (**Figure 4N**). Of the 370 KIRC samples documented in the GSCA database, only 1 case showed a mutation in CRHBP (**Figure 4O**); the same was true in the TIMER database (**Figure 4P**). In the UALCAN database, MFSD4A promoter methylation levels were significantly higher in tumour tissue (**Figure 4Q**), CRHBP was significantly lower (**Figure 4R**), and MPP7 was significantly higher (**Figure 4S**). In the GSCA database, with missing data for MFSD4A, CRHBP, and MPP7 were indeed differentially expressed in tumour tissues (**Figure 4T**, FDR ≤ 0.05), and only CRHBP showed a significant correlation between methylation levels and mRNA expression levels (**Figure 4U**). On the contrary, CRHBP presents high methylation levels of KIRC in the GSCA database (**Figure 4V**). As for CRHBP methylation levels and prognosis: DFI (log_rank_p=0.76, cox_p=0.76, HR = 1.18, **Figure 4W**), DSS (log_rank_p=2.44E-05, cox_p=6.36E-05, HR = 3.14, **Figure 4X**), OS (log_rank_p=1.14E-03, cox_p=1.40E-03, HR = 1.93, **Figure 4Y**), PFS (log_rank_p=8.06E-05, cox_p=1.09E-04, HR = 1.98, **Figure 4Z**). HR point estimates for methylation and prognosis were 3.14, 1.93, 1.98; hence, HR for methylation (High methylation vs Low methylation) = 1.98 [1.93, 3.14]. The MethHC 2.0 database validation showed that CRHBP methylation levels had no significant effect on KIRC prognosis, HR = 0.2068 [-3.278, 0.7008], P value = 0.2812 (**Figure 4AA**). These results show that CRHBP is a biomarker with a tangible effect on prognosis, confirmed by multiple data.

### 3.4 Immune infiltration algorithm result

**Figure 5:**
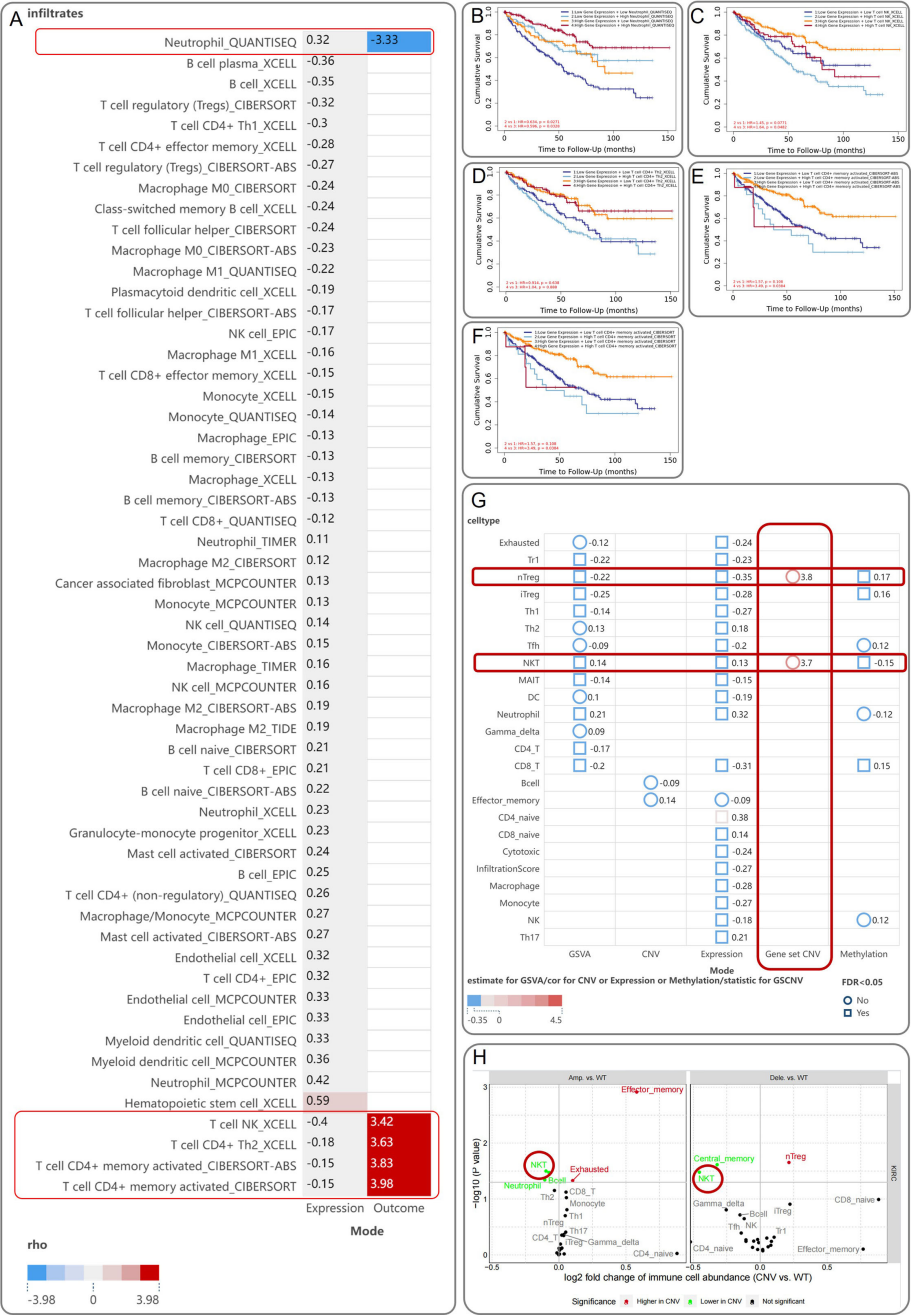
Associations between CRHBP-related immune infiltration and gene expression, clinical prognosis, GSVA score, and methylation levels in KIRC. **(A)** Visualization of immune infiltration results with adjusted P-value < 0.05 from the TIMER database. On the x-axis (left to right), the first column shows significant correlations between immune infiltration and CRHBP expression, and the second column shows significant correlations between immune infiltration and clinical outcome. The y-axis represents different immune cell types, where the prefix before “_” indicates the immune cell type and the suffix after “_” indicates the immune infiltration deconvolution algorithm used. RHO values represent correlation coefficients: higher values (redder) indicate stronger positive correlations, lower values (bluer) indicate stronger negative correlations, and white indicates no correlation. Regions highlighted with red boxes indicate results that show strong correlations in both expression and clinical outcome analyses. **(B)** Combined prognostic impact of CRHBP expression and neutrophil infiltration levels predicted by the QUANTISEQ algorithm. **(C)** Combined prognostic impact of CRHBP expression and natural killer T (NKT) cell infiltration levels predicted by the XCELL algorithm. **(D)** Combined prognostic impact of CRHBP expression and CD4^+^ helper T cell type 2 (Th2) infiltration levels predicted by the XCELL algorithm. **(E)** Combined prognostic impact of CRHBP expression and activated CD4^+^ memory T cell infiltration levels predicted by the CIBERSORT-ABS algorithm. **(F)** Combined prognostic impact of CRHBP expression and activated CD4^+^ memory T cell infiltration levels predicted by the CIBERSORT algorithm. **(G)** Visualization of correlations between immune infiltration results (P < 0.05) and four omics modalities from the GSCA database. The “estimate” value represents the magnitude of correlation for GSVA score, copy number variation (CNV), gene expression, and methylation levels: higher values (redder) indicate stronger positive correlations, lower values (bluer) indicate stronger negative correlations, and white indicates no correlation. Square markers indicate FDR < 0.05, and circular markers indicate FDR > 0.05. Horizontal red boxes highlight results that show strong correlations across all four omics modalities (GSVA, expression, gene set CNV, and methylation). Vertical red boxes highlight results that show significant correlations specifically in the gene set CNV analysis. **(H)** Differences in immune cell infiltration levels between different CNV states (amplification/deletion) and wild-type (WT) in KIRC. The x-axis shows log2-transformed fold changes in immune cell infiltration levels, and the y-axis shows -log10(P-value). The left column represents Amplification vs. WT, and the right column represents Deletion vs. WT. Red indicates higher abundance in the CNV group, and green indicates lower abundance in the CNV group. Immune cell types common to both CNV states are circled in red.

**Figure 6:**
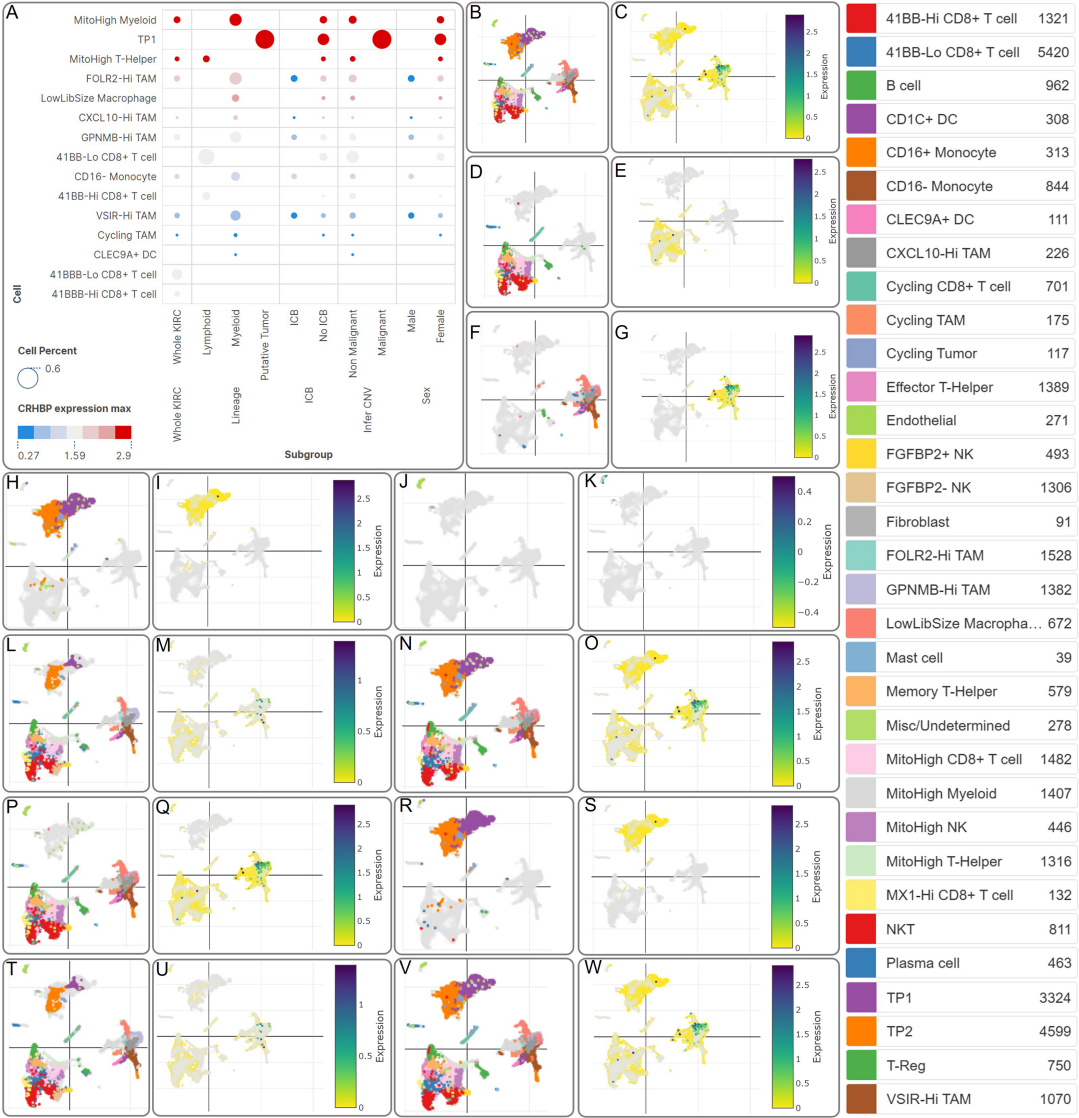
The true expression of CRHBP in various immune cells in a single-cell dataset. Correspondence between colors and immune cells, as well as CRHBP expression, was recorded in **Supplementary Table 4**. **(A)** CRHBP expression in 15 types of cells in the kidney tissues of KIRC. **(B)** Planimetric distribution of various immune cells in Whole KIRC. **(C)** Expression of CRHBP in various immune cells in Whole KIRC, where yellowish color indicates lower expression and darker color indicates higher expression. **(D)** Planar distribution of various immune cells when Lineage=Lymphoid. **(E)** Expression of CRHBP in various immune cells at Lineage=Lymphoid, where yellow indicates lower expression and black indicates higher expression. **(F)** Planar distribution of various immune cells when Lineage=Myeloid. **(G)** Expression of CRHBP in various immune cells at Lineage=Myeloid, where yellow indicates lower expression and black indicates higher expression. **(H)** Planar distribution of various immune cells when Lineage=Putative Tumor. **(I)** Expression of CRHBP in various immune cells at Lineage=Putative Tumor, where yellowish color indicates lower expression and blackish color indicates higher expression. **(J)** Planar distribution of various immune cells when Lineage=Normal Tissue. **(K)** Expression of CRHBP in various immune cells at Lineage=Normal Tissue, where yellowish color indicates lower expression and darker color indicates higher expression. **(L)** Planar distribution of various immune cells when exposed to ICB. **(M)** Expression of CRHBP in various immune cells upon exposure to ICB, where yellow indicates lower expression and black indicates higher expression. **(N)** Planar distribution of various immune cells when not exposed to ICB. **(O)** Expression of CRHBP in various immune cells when not exposed to ICB, yellowish color indicates lower expression, blackish color indicates higher expression. **(P)** Planar distribution of various immune cells when Non-Malignant. **(Q)** Expression of CRHBP in various immune cells at the time of Non-Malignant, where yellowish color indicates lower expression and blackish color indicates higher expression. **(R)** Planar distribution of various immune cells at the time of malignancy. **(S)** Expression of CRHBP in various immune cells at the time of malignant, yellow color indicates lower expression, and black color indicates higher expression. **(T)** Planar distribution of various immune cells in Males. **(U)** Expression of CRHBP in various types of immune cells at the time of Male, where yellow indicates lower expression and black indicates higher expression. **(V)** Plot of various immune cells in Females. **(W)** Expression of CRHBP in various immune cells at the time of female, yellow color indicates lower expression, and black color indicates higher expression.

Screening of the immune infiltration results of the TIMER database for adjusted P<0.05 fractions revealed five types of immune cells with significant correlations with both gene expression and prognosis: Neutrophil, Hematopoietic stem cell, T cell NK, T cell CD4+ Th2, T cell CD4+, T cell CD4+, and T cell CD4+ memory activated (**Figure 5A**). The effects of high and low levels of these five immune cells on overall survival are presented in **Figures 5B**-**6F**. Under conditions of low gene expression, a high neutrophil state showed better prognosis compared to a low neutrophil state (HR = 0.634, P = 0.0271, **Figure 5B**); under conditions of high gene expression, a high neutrophil state demonstrated better prognosis compared to a low neutrophil state (HR = 0.596, P = 0.0328, **Figure 5B**), a high NKT cell state exhibited worse prognosis compared to a low NKT cell state (HR = 1.64, P = 0.0482, **Figure 5C**), a high CD4+ memory activated T cell state showed worse prognosis compared to a low CD4+ memory activated T cell state (HR = 3.49, P = 0.0384, **Figure 5E**), and the adverse risk effect of a high CD4+ memory activated T cell state remained consistent across both CIBERSORT and CIBERSORT_ABS methods (**Figures 5E, F**). The two immune cells, NKT and nTreg, in the GSCA database exemplify significant associations with the more multidimensional (GSVA, Expression, Gene set CNV, Methylation) data (**Figure 5G**, raw results of immune infiltration of TIMER and GSCA are in **Supplementary Table 2**). The degree of infiltration of nTreg was roughly deletion group > amplification group > wild type group (WT, **Figure 5H**), and the degree of infiltration of NKT was roughly wild type group (WT) > amplification group > deletion group (**Figure 5I**) in different gene set CNV groups.

### 3.6 Immune cells based on scRNA

The results of all subgroup analyses of CRHBP in single-cell data (raw information of single-cell dataset in **Supplementary Table 3**, raw immune cell proportion and gene expression results in **Supplementary Table 4**) are visualised in **Figures 6A–W**; where, in MitoHigh Myeloid, TP1, and MitoHigh T-Helper were all highly expressed; conversely, they were all lowly expressed in VSIR-Hi TAM, Cycling TAM, and CLEC9A+ DC. It should also be noted that in FOLR2-Hi TAM and CXCL10-Hi TAM cells, the expression was lower when exposed to ICB than when unexposed, and lower in males than in females (**Figure 6A**).

### 3.7 Drug sensitivity and gene-chemical interaction network

**Figure 7:**
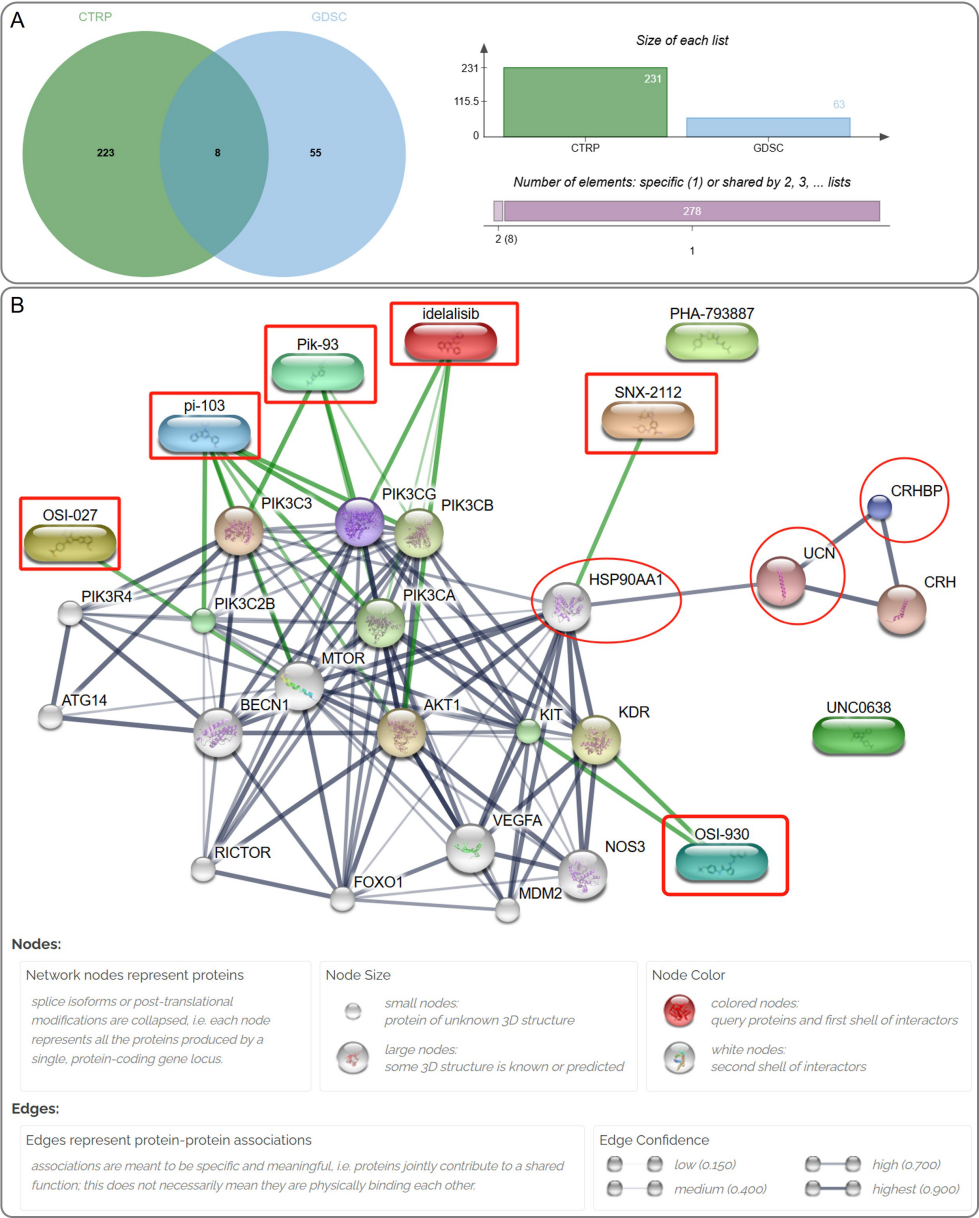
Intersection results and possible interactions of sensitive drugs predicted by CRHBP in both CTRP and GDSC databases. **(A)** Intersection results of CRHBP predicted sensitivity drugs in both CTRP and GDSC databases for 8 drugs (CAL-101, OSI-027, OSI-930, PHA-793887, PI-103, PIK-93, SNX-2112, UNC0638). **(B)** Network diagram of the interaction of the 8 sensitizing drugs with CRHBP.

Based on the CRHBP-sensitive drugs analyzed in the CTRP and GDSC databases of the GSCA online server, 8 drugs were obtained after screening for P<0.05, taking the intersection (CAL-101, OSI-027, OSI-930, PHA-793887, PI-103, PIK-93, SNX-2112, UNC0638). The Raw data in **Supplementary Table 5**, **Figure 7A**. Six drugs among eight (CAL-101 or Idelalisib, OSI-027, OSI-930, PI-103, PIK-93, SNX-2112) were found to have an indirect effect on CRHBP after drug-protein interaction analyses in the STITCH database (**Figure 7B**). PPI networks suggest that CRHBP closely related proteins include 20 types: UCN, CRH, HSP90AA1, PIK3CB, PIK3CG, PIK3C3, PIK3R4, PIK3C2B, PIK3CA, ATG14, MTOR, BECN1, AKT1, KIT, KDR, RICTOR, FOXO1, VEGFA, NOS3, MDM2 (**Figure 7B**).

### 3.8 Two-sample mendelian randomization of genes and immune cells

**Figure 8:**
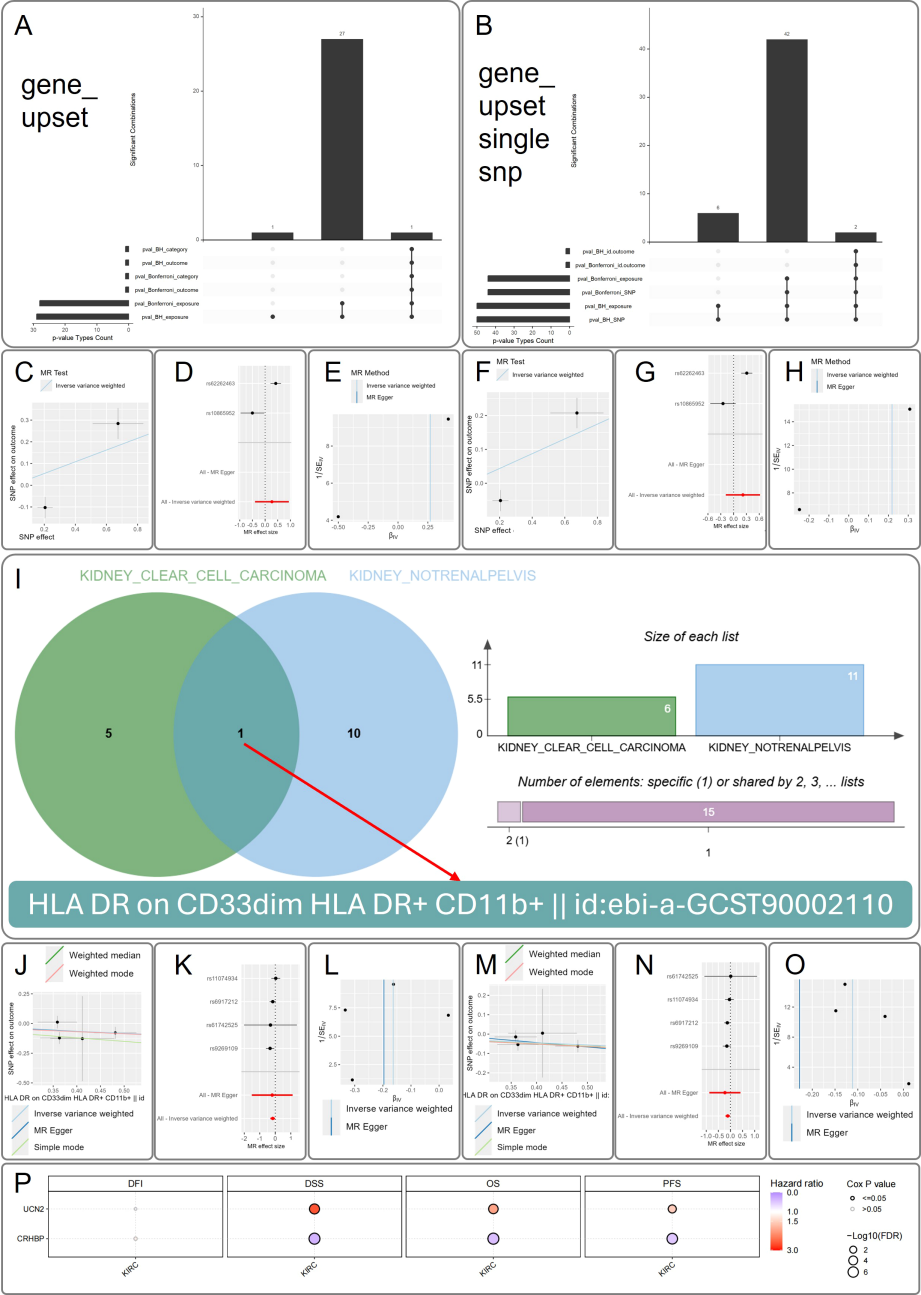
TSMR intersection results. **(A)** Intersection results of gene-level primary results. Rows represent different multiple correction methods (row numbers indicate the number of results included in each method), and columns represent intersection details (column numbers indicate the number of results included in each intersection). The number of dots in a column indicates the number of multiple correction methods in which the intersection exists. **(B)** Intersection results of gene-level single-SNP analysis. **(C-E)** TSMR result of UCN2 (rs62262463 was in Testis) in finngen_R10_C3_KIDNEY_CLEAR_CELL_CARCINOMA_EXALLC. **(F-H)** TSMR result of UCN2 in finngen_R10_C3_KIDNEY_NOTRENALPELVIS_EXALLC. **(I)** The intersection of immune cells with P values less than 0.05 after BH and Bonferroni correction in two KIRC queues. The intersecting immune cells are HLA DR on CD33dim HLA DR+ CD11b+ || id:ebi-a-GCST90002110. **(J-L)** TSMR result of HLA DR on CD33dim HLA DR+ CD11b+ in finngen_R10_C3_KIDNEY_CLEAR_CELL_CARCINOMA_EXALLC. **(M-O)** TSMR result of HLA DR on CD33dim HLA DR+ CD11b+ in finngen_R10_C3_KIDNEY_NOTRENALPELVIS_EXALLC.

**Figure 9:**
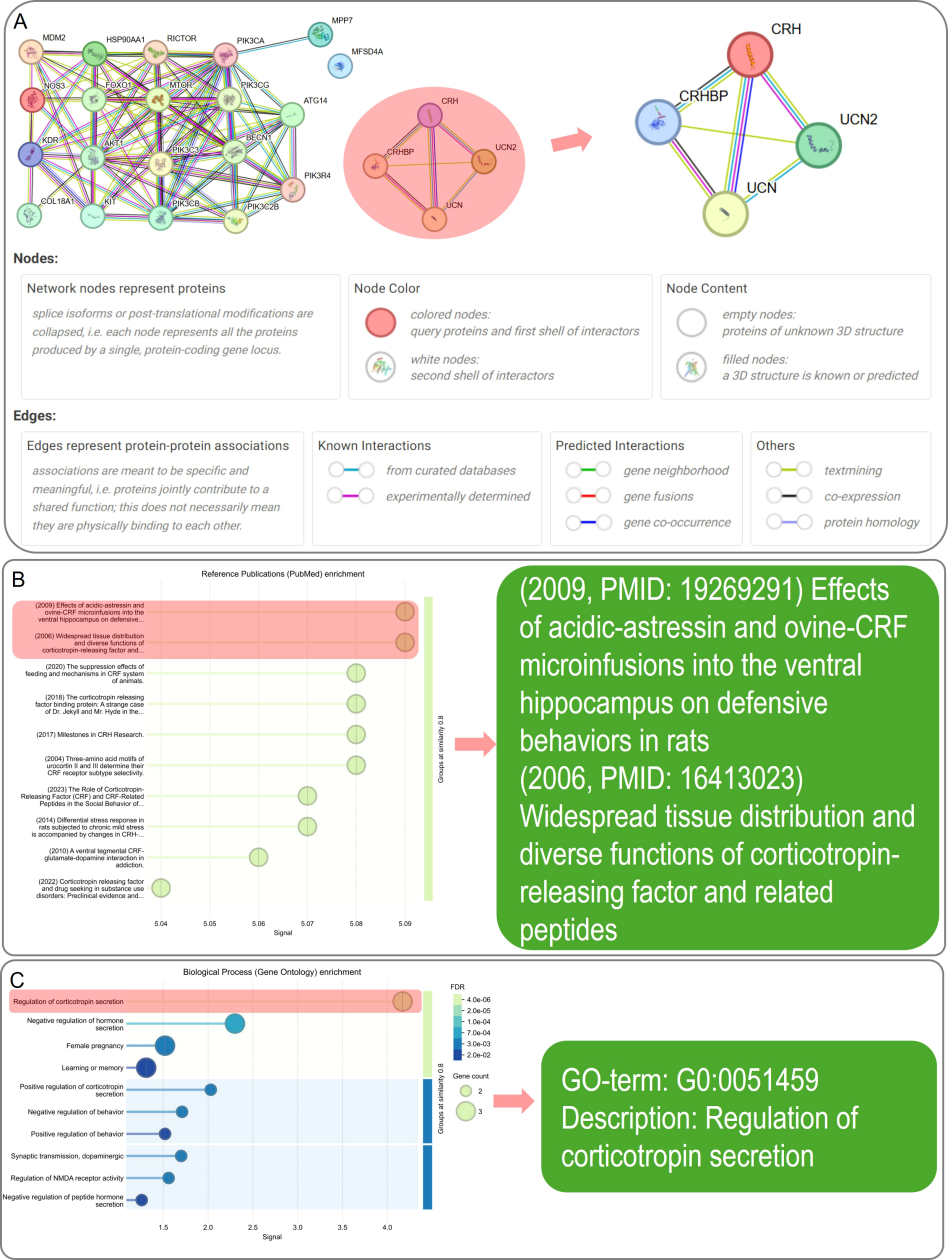
Protein-protein interaction and enrichment analysis. **(A)** Protein-protein interaction network. Each circle represents a protein, and connecting lines indicate interactions. CRH, UCN2, UCN, and CRHBP form a functionally clustered group and were selected for subsequent enrichment analysis. **(B)** Literature enrichment results for CRH, UCN2, UCN, and CRHBP. The two references with the highest signal values are highlighted with red boxes. **(C)** Gene Ontology (GO) biological process enrichment results for CRH, UCN2, UCN, and CRHBP. The GO term with the highest signal value is highlighted with a red box.

Proteins identified through UALCAN, Gepia, and STITCH totaled 23 (MFSD4, CRHBP, MPP7, UCN, CRH, HSP90AA1, PIK3CB, PIK3CG, PIK3C3, PIK3R4, PIK3C2B, PIK3CA, ATG14, MTOR, BECN1, AKT1, KIT, KDR, RICTOR, FOXO1, VEGFA, NOS3, MDM2). Their corresponding genes were analyzed in GTEx eQTL and two KIRC cohorts. For TSMR primary results (**Figure 8A**, original data in **Supplementary Table 6**), after Bonferroni and BH corrections, one significant gene (UCN2_Testis, SNP = rs62262463) remained. For single-SNP results (**Figure 8B**, original data in **Supplementary Table 7**), two significant results (UCN2_Testis in finngen_R10_C3_KIDNEY_CLEAR_CELL_CARCINOMA_EXALLC/finngen_R10_C3_KIDNEY_NOTRENALPELVIS_EXALLC, all intersections in **Supplementary Table 8**. Detailed visualizations of TSMR for the two KIRC cohorts were presented in **Figures 8C**-**9E** and **Figures 8F**-**9H**, respectively). Both gene main result and single-SNP results indicated a risk effect for UCN2_Testis: OR (finngen_R10_C3_KIDNEY_CLEAR_CELL_CARCINOMA_EXALLC) = 1.52405624953891 [1.238671577, 1.875192339], P = 6.79445E-05; OR (finngen_R10_C3_KIDNEY_NOTRENALPELVIS_EXALLC) = 1.360501649 [1.194500751, 1.549571849], P = 3.53471E-06.

For immune cell analyses, 6/11 types of immune cells met preliminary thresholds ((Adjusted) P value < 0.05 and nsnp > 3) in finngen_R10_C3_KIDNEY_CLEAR_CELL_CARCINOMA_EXALLC/finngen_R10_C3_KIDNEY_NOTRENALPELVIS_EXALLC. Both cohorts intersect with one type of immune cell: HLA DR on CD33dim HLA DR+ CD11b+ || id:ebi-a-GCST90002110 (**Figure 8I**). In finngen_R10_C3_KIDNEY_CLEAR_CELL_CARCINOMA_EXALLC: OR (Inverse variance weighted) = 0.849301444 [0.731059311, 0.986668157], P value = 0.032722764, pval_BH = 0.032722764, pval_Bonferroni = 0.032722764 (Detailed visualizations of TSMR was presented in **Figures 8J–****9L**, respectively). In finngen_R10_C3_KIDNEY_NOTRENALPELVIS_EXALLC: OR (Inverse variance weighted) = 0.894468162 [0.817702372, 0.978440714], P value = 0.014847729, pval_BH = 0.014847729, pval_Bonferroni = 0.014847729; OR (Weighted median) = 0.879186835 [0.792157314, 0.975777762], P value = 0.015475194, pval_BH = 0.015475194, pval_Bonferroni = 0.015475194 (Detailed visualizations of TSMR was presented in **Figures 8M**-**9O**, respectively).

### 3.9 Protein-protein interactions and enrichment analysis result

Proteins identified through UALCAN, Gepia, STITCH, and TSMR totaled 24 (MFSD4, CRHBP, MPP7, UCN2, UCN, CRH, HSP90AA1, PIK3CB, PIK3CG, PIK3C3, PIK3R4, PIK3C2B, PIK3CA, ATG14, MTOR, BECN1, AKT1, KIT, KDR, RICTOR, FOXO1, VEGFA, NOS3, MDM2). UCN2 and CRHBP were hub genes/proteins. STRING-based PPI analysis revealed that UCN2, the risk protein identified by TSMR, interacted only with three other proteins (CRHBP, UCN, and CRH; **Figure 9A**). Enrichment analysis of CRHBP, UCN2, UCN, and CRH in STRING highlighted two major literature references: “(2009, PMID: 19269291) Effects of acidic-astressin and ovine-CRF microinfusions into the ventral hippocampus on defensive behaviors in rats” and “(2006, PMID: 16413023) Widespread tissue distribution and diverse functions of corticotropin-releasing factor and related peptides” (**Figure 9B**). Gene Ontology (GO) enrichment identified “GO-term: G0:0051459 Regulation of corticotropin secretion” as the most significant pathway (**Figure 9C**; original enrichment data in **Supplementary Table 12**).

### 3.10 Molecular docking and interaction analysis result

**Figure 10:**
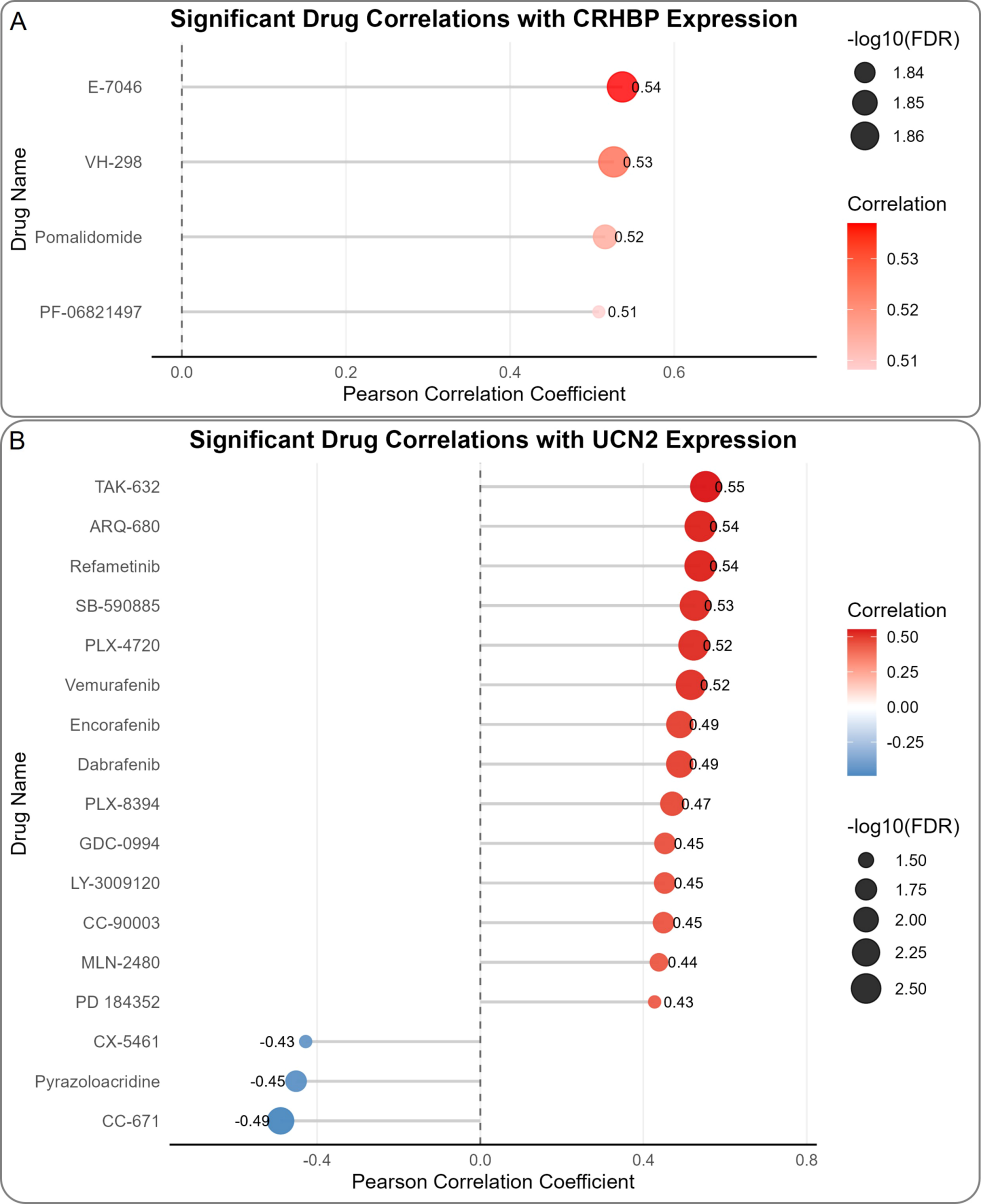
Candidate drug screening based on CellMiner. **(A)** Highly correlated drugs with CRHBP, with circle size correlated with -log10(FDR), darker colors indicating stronger correlation, the x-axis representing correlation, and the y-axis representing different drugs. **(B)** Highly correlated drugs with UCN2, where the size of the circles is correlated with -log10(FDR), and darker colors indicate stronger correlations. Red indicates positive correlation, and blue indicates negative correlation. The x-axis represents correlation, and the y-axis represents different drugs.

Based on CellMiner screening, four drugs were found to be highly correlated with CRHBP (**Figure 10A**), E-7046 (FDA_status = Clinical trial, Correlation = 0.54, FDR = 1.35E-02), VH-298 (FDA_status = Clinical trial, Correlation = 0.53, FDR = 1.35E-02), Pomalidomide (FDA_status = FDA approved, Correlation = 0.52, FDR = 1.42E-02), PF-06821497 (FDA_status = Clinical trial, Correlation = 0.51, FDR = 1.47E-02). Based on CellMiner, 17 drugs were screened that are highly correlated with UCN2 (**Figure 10B**), TAK-632 (FDA_status = Clinical trial, Correlation = 0.55, FDR = 2.59E-03), ARQ-680 (FDA_status = Clinical trial, Correlation = 0.54, FDR = 2.59E-03), Refametinib (FDA_status = Clinical trial, Correlation = 0.54, FDR = 2.59E-03), CX-5461 (FDA_status = Clinical trial, Correlation = -0.43, FDR = 3.39E-02), Pyrazoloacridine (FDA_status = Clinical trial, Correlation = -0.45, FDR = 1.96E-02), CC-671 (FDA_status = Clinical trial, Correlation = -0.49, FDR = 7.13E-03).

**Figure 11:**
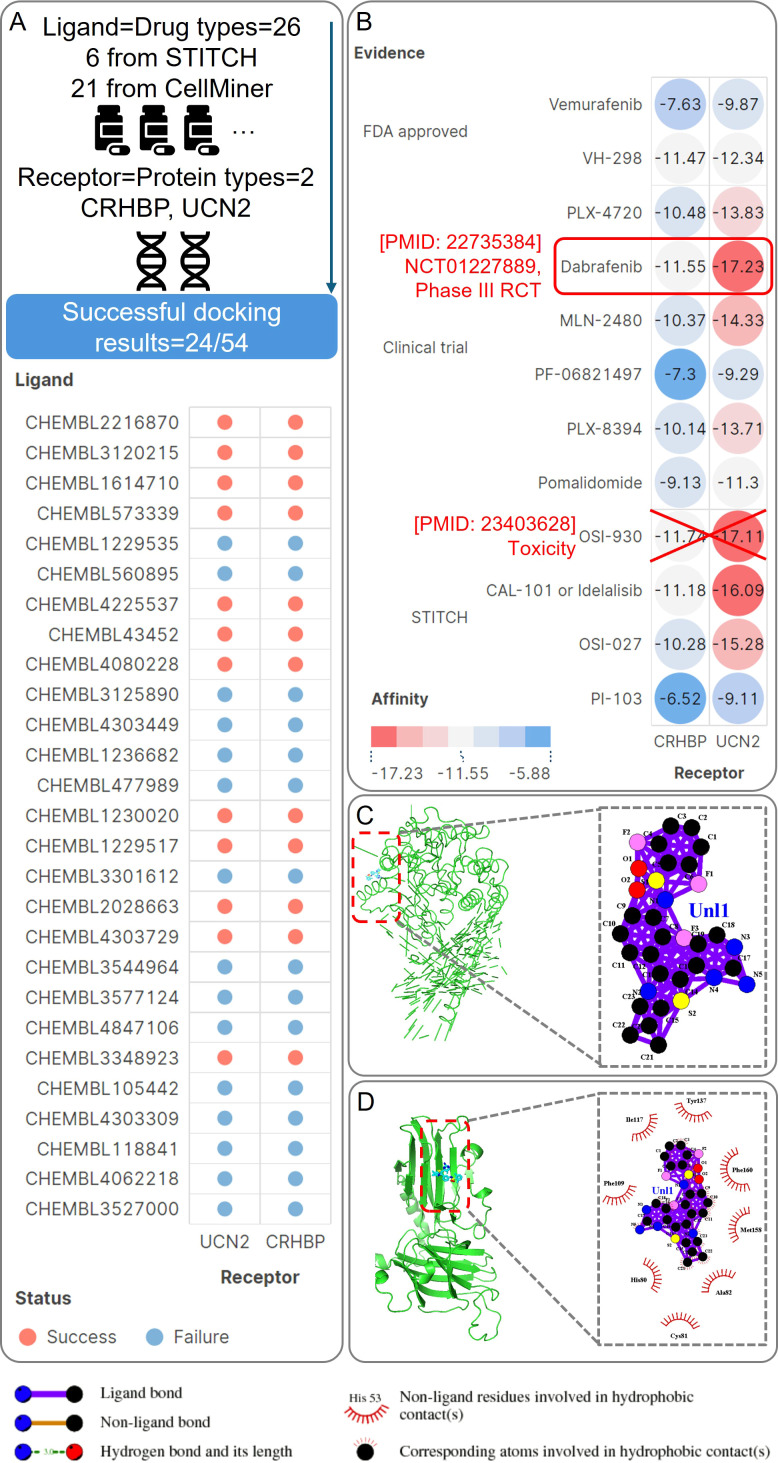
Molecular docking results. **(A)** Details of the success or failure of docking between two proteins and 27 drugs, with a total of 54 results. Red indicates successful docking; blue indicates failed docking. **(B)** Heat map visualization of the affinity of CRHBP and UCN2 with 12 drugs, with colors ranging from red to white to blue representing affinity from strong to weak. **(C)** The docking 3D structure (left) and interaction visualization (right) of dabrafenib with UCN2. **(D)** The docking 3D structure (left) and interaction visualization (right) of dabrafenib with CRHBP. The legend for the molecular docking interface analysis visualization is located at the bottom.

The 6 drugs identified by STITCH and the 21 drugs identified by CellMiner, totaling 27 drugs, were subjected to molecular docking with CRHBP and UCN2, respectively. Among the 54 docking records, only 24 yielded successful results, meaning that CRHBP and UCN2 successfully docked with 12 drugs (**Figure 11A**). Among the 12 drugs, 2 are FDA-approved, 6 are in clinical trials, and 4 are sensitivity drugs obtained through STITCH (**Figure 11B**). Two drugs have particularly low affinity: dabrafenib (affinity with CRHBP = -11.55, affinity with UCN2 = -17.23) and OSI-930 (affinity with CRHBP=-11.74, affinity with UCN2=-17.11), but OSI-930 was excluded due to its toxicity [PMID: 23403628]; hence, dabrafenib was considered the most promising candidate drug [PMID: 22735384]. The docking 3D structure and interaction visualization of dabrafenib with UCN2 are shown in **Figure 11C** (no hydrogen bonds detected), while the docking 3D structure and interaction visualization of dabrafenib with CRHBP are shown in **Figure 11D** (8 hydrogen bonds detected).

### 3.11 Hub protein expression under different stages and immune stimulation responses

**Figure 12:**
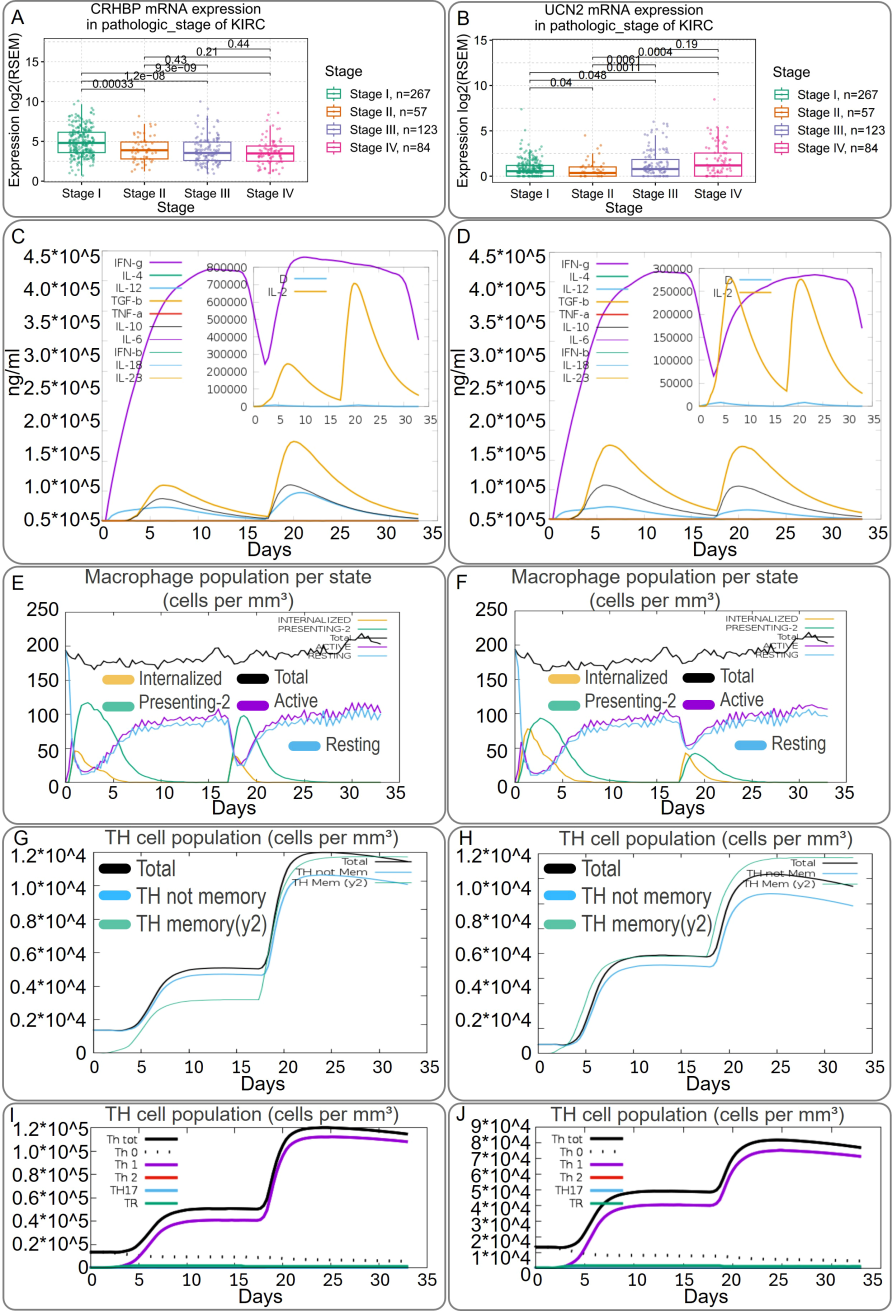
Hub gene expression under different stages and immune stimulation responses. The box plots for CRHBP **(A)** and UCN2 **(B)** expression across different stages show stage I in green, stage II in orange, stage III in purple, and stage IV in pink, with the X-axis representing disease stages and the Y-axis indicating expression levels. The changes in cytokines induced by CRHBP **(C)** and UCN2 **(D)** are depicted with the X-axis showing days and the Y-axis indicating cytokine concentration (ng/ml), where different colors represent distinct cytokine types. The dynamics of macrophage subsets induced by CRHBP **(E)** and UCN2 **(F)** are illustrated with the X-axis for days and the Y-axis for cell concentration (cells per mm^3^), using different colors for various activation states. The alterations in T Helper **(TH)** cell memory status induced by CRHBP **(G)** and UCN2 **(H)** feature the X-axis as days and the Y-axis as cell concentration (cells per mm^3^), with distinct colors denoting different memory states. Finally, the changes in TH cell subtypes induced by CRHBP **(I)** and UCN2 **(J)** are shown with the X-axis indicating days and the Y-axis reflecting cell concentration (cells per mm^3^), where colors differentiate between specific TH cell subsets.

Based on the GSCA database, CRHBP expression exhibits a stepwise decreasing trend as the disease stage progresses (**Figure 12A**), while UCN2 expression similarly shows a gradual incline with advancing disease stage (**Figure 12B**). As epitope peptides, CRHBP induces high levels of IFN-γ (4.0–4.5 × 10^5^ ng/ml) at 10–15 days and 20–30 days (**Figure 12C**), and elevated IL-2 (2 × 10^5^ ng/ml at day 7 and 7 × 10^5^ ng/ml at day 21, **Figure 12C**); UCN2 induces high levels of IFN-γ (4.0 × 10^5^ ng/ml) during 10–15 days and 25–30 days (**Figure 12D**), along with elevated IL-2 (2.7 × 10^5^ ng/ml) at day 7 and day 21 (**Figure 12D**). Both CRHBP and UCN2 maintain active and resting macrophages at approximately 100 cells per mm^3^ after day 30 (**Figures 12E, F**). Additionally, both peptides stabilize TH memory and TH non-memory cells around 1.0 × 10^4^ after day 25 (**Figures 12G, H**), with UCN2 inducing a higher number of TH memory cells compared to the total TH cells (**Figure 12H**). For specific TH subtypes, CRHBP and UCN2 induce high levels of total TH and Th1 cells at day 23, and CRHBP elicits a greater TH cell count than UCN2 (1.1 × 10^5^ > 8 × 10^4^, **Figures 12I, J**).

**Figure 13:**
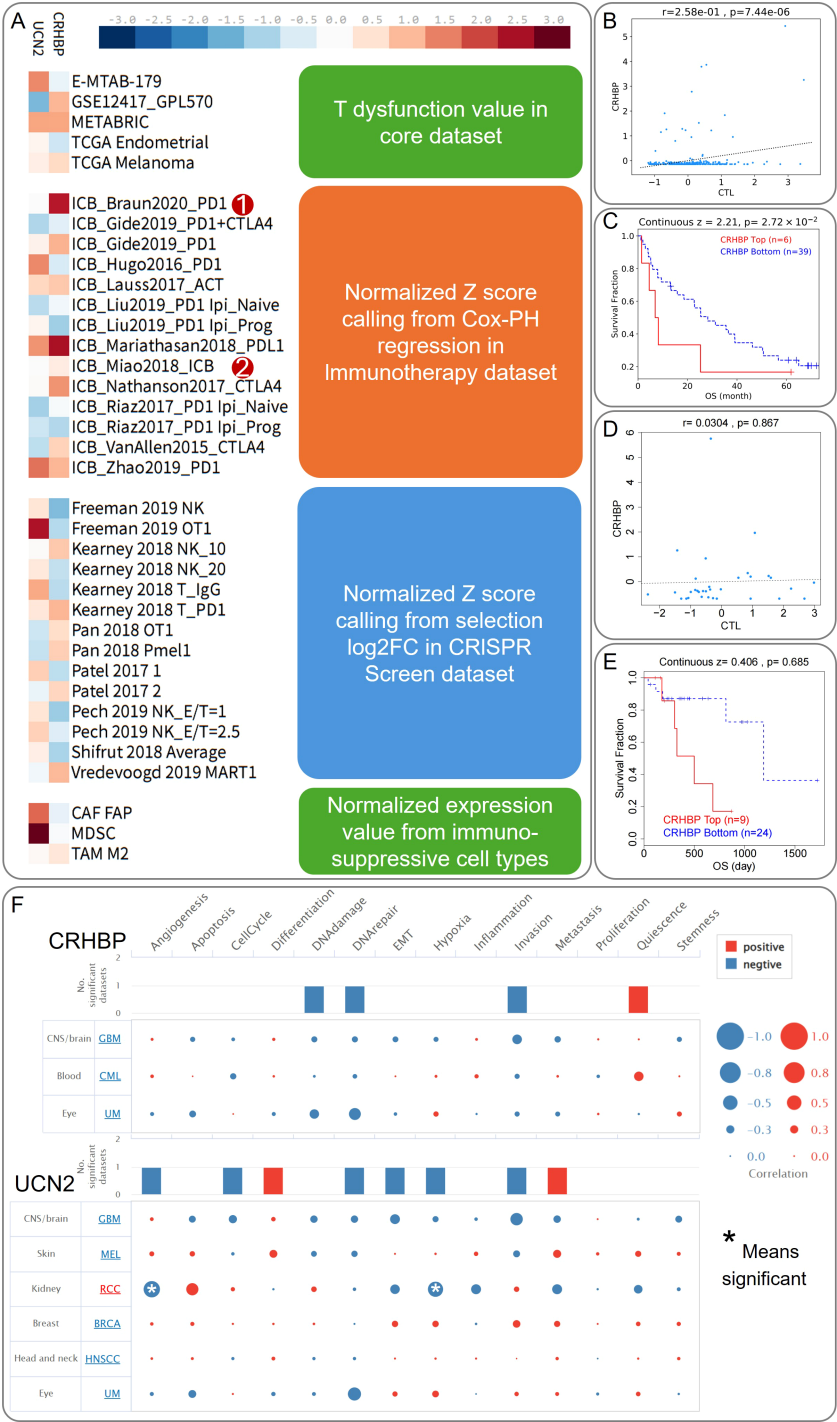
The association between hub genes and patient treatment response and their functional status in tumor cells. **(A)** TIDE results include four datasets: (1) T dysfunction value in core dataset, containing 5 results and marked in blue; (2) Normalized Z score calling from Cox-PH regression in immunotherapy dataset, containing 14 results and marked in orange; (3) Normalized Z score calling from selection log2FC in CRISPR Screen dataset, containing 14 results and marked in blue; and (4) Normalized expression value from immuno-suppressive cell types, containing 3 results and marked in green. In the left heatmap, color depth indicates the absolute value of Z Score (white for Z Score = 0, blue for Z Score < 0, red for Z Score > 0), with red circles and numbers highlighting studies closely related to KIRC—Study 1 (ICB_Braun2020_PD1) and Study 2 (ICB_Miao2018_ICB); **(B)** Scatter plot visualizing the correlation between CRHBP and Cytotoxic T Lymphocytes (CTLs) in Study 1; **(C)** Kaplan-Meier curve for prognosis based on CRHBP expression in Study 1, where the top CRHBP cohort (red solid line, n=6) represents the top 15% of patients with highest CRHBP expression and the bottom cohort (blue dashed line, n=39) represents the bottom 85% with lowest expression, with Z score roughly equivalent to HR; **(D)** Scatter plot for CRHBP-CTLs correlation in Study 2; **(E)** Kaplan-Meier curve for Study 2, with the top CRHBP cohort (red solid line, n=9) and bottom cohort (blue dashed line, n=24), similarly using Z score as HR proxy; **(F)** Heatmap showing correlations of genes (CRHBP and UCN2) across 14 states in various cancers, where bar height denotes the number of significant datasets, color indicates direction (blue: negative correlation/suppression, red: positive correlation/activation), circle size reflects correlation strength (larger for stronger correlations), and asterisks (*) mark statistically significant associations.

The TIDE database indicates that CRHBP and UCN2 are closely associated across four datasets: (1) T dysfunction value in core dataset, containing 5 results; (2) Normalized Z score calling from Cox-PH regression in immunotherapy dataset, containing 14 results; (3) Normalized Z score calling from selection log2FC in CRISPR Screen dataset, containing 14 results; and (4) Normalized expression value from immuno-suppressive cell types, containing 3 results (**Figure 13A**). Among these, only CRHBP shows significant associations in KIRC within the “Normalized Z score calling from Cox-PH regression in immunotherapy dataset,” with two notable results: Z score for ICB_Braun2020_PD1 = 2.346291 and Z score for ICB_Miao2018_ICB = 0.323296722986 (original data in **Supplementary Table 14**). In the first study (ICB_Braun2020_PD1), CRHBP’s correlation with Cytotoxic T Lymphocytes (CTLs) revealed r = 2.58E^-01^ and P value = 7.44E^-06^ (**Figure 13B**). The Kaplan-Meier curve demonstrated a reversal effect in KIRC patients treated with PD1 inhibitors in Braun et al. (2020), where the low-CRHBP-expression group exhibited significantly better prognosis than the high-expression group (Z score = 2.21 > 1, P value = 2.72×10^-2^ < 0.05, **Figure 13C**). In the second study (ICB_Miao2018_ICB), CRHBP’s correlation with CTLs similarly showed r = 0.0304 and P value = 0.867 (**Figure 13D**), but the KM curve indicated no significant prognostic difference between low- and high-CRHBP-expression groups in Miao et al. (2018) under ICB treatment (Z score = 0.406 < 1, P value = 0.685 > 0.05, **Figure 13E**). To further explain this therapeutic discrepancy, CancerSEA database results from single-cell RNA sequencing revealed no specific tumor cell regulation of CRHBP in KIRC; however, UCN2 exhibited significant negative correlations with angiogenesis (r = -0.72, P = 0.017) and hypoxia (r = -0.664, P = 0.031) in KIRC tumor cells, indicating that UCN2 suppresses angiogenesis and hypoxia in KIRC (**Figure 13F**).

### 3.12 In silico knockout result

**Figure 14:**
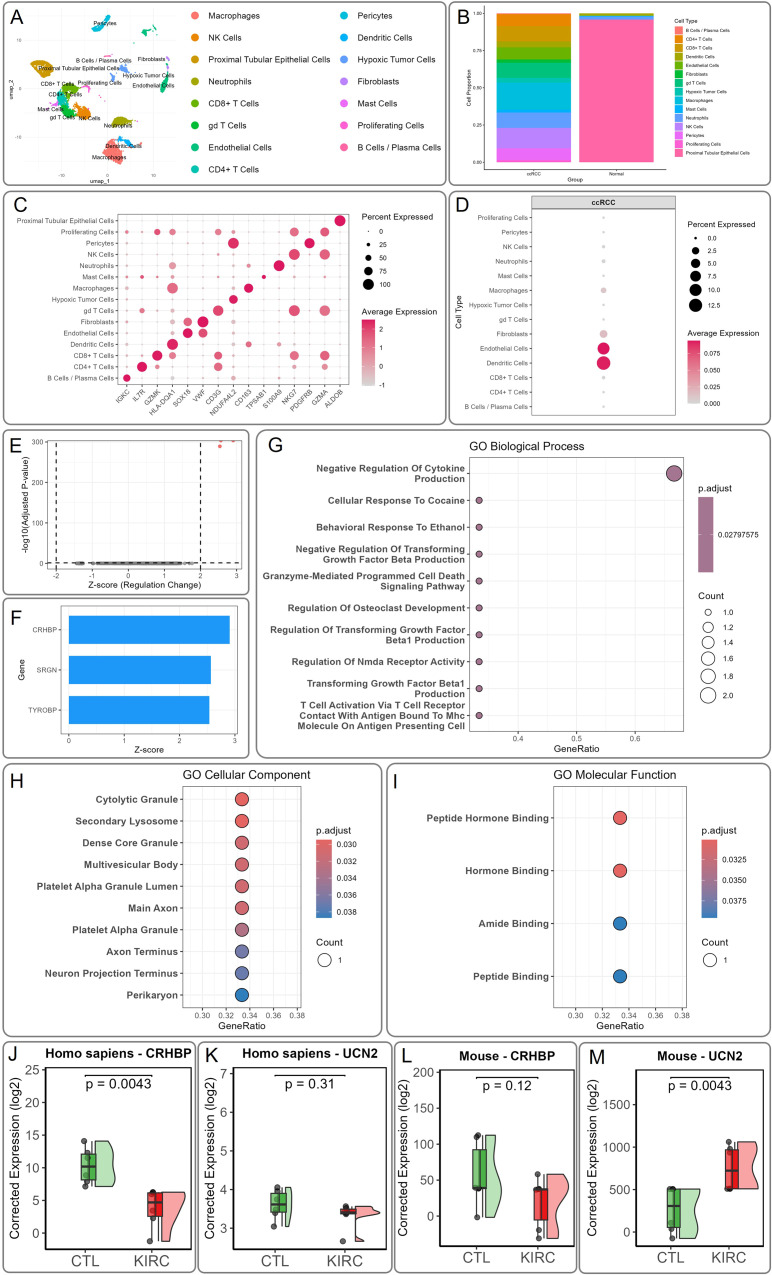
Virtual knockout and multi-species PCR validation. **(A)** UMAP dimensionality reduction plot showing 15 cell subtypes annotated after data quality control and filtering, with different colors representing distinct cell types. **(B)** Stacked bar chart comparing the proportional distribution of various cell types in ccRCC tissues and normal tissues, where different colors correspond to different cell types. **(C)** Dot plot for validation of cell marker genes. The horizontal axis represents marker genes, and the vertical axis indicates cell types. The dot size denotes the percentage of cells expressing the gene, and the dot color reflects the average expression level. **(D)** Dot plot of CRHBP expression in ccRCC tissues. The dot size represents the percentage of gene-expressing cells, and the dot color indicates the average gene expression level. **(E)** Volcano plot of differentially regulated genes after CRHBP virtual knockout. The horizontal axis is Z-score (fold change), and the vertical axis is -log10(adjusted P value). Red dots represent genes with significant expression differences. **(F)** Bar plot of significantly differentially regulated genes following CRHBP virtual knockout. The horizontal axis is Z-score (fold change), and the vertical axis lists the names of differentially expressed genes. **(G)** Bubble plot of GO Biological Process (BP) enrichment analysis for differential genes. The horizontal axis represents GeneRatio (the proportion of input genes in pathway background genes). The dot color corresponds to adjusted P value, and the dot size is positively correlated with the number of enriched genes. **(H)** GO Cellular Component (CC) enrichment analysis of differential genes. **(I)** GO Molecular Function (MF) enrichment analysis of differential genes. **(J)** Raincloud plot of CRHBP expression validated by PCR in human samples. Green indicates the control group (CTL), red indicates the KIRC group, and the vertical axis represents the relative gene expression level. **(K)** Raincloud plot of UCN2 expression validated by PCR in human samples. Green represents the CTL group, red represents the KIRC group, and the vertical axis shows the relative gene expression level. **(L)** Raincloud plot of CRHBP expression validated by PCR in mouse samples. Green denotes the CTL group, red denotes the KIRC group, and the vertical axis indicates the relative gene expression level. **(M)** Raincloud plot of UCN2 expression validated by PCR in mouse samples. Green represents the CTL group, red represents the KIRC group, and the vertical axis shows the relative gene expression level. UMAP, Uniform Manifold Approximation and Projection; GO, Gene Ontology; ccRCC, clear cell renal cell carcinoma; CTL, control group; KIRC, kidney renal clear cell carcinoma.

After data cleaning, the GSE152938 dataset was annotated into 15 cell types (**Figure 14A**). Clear cell renal cell carcinoma (ccRCC), equivalent to kidney renal clear cell carcinoma (KIRC), harbored higher proportions of various immune cell subtypes compared with normal tissues (**Figure 14B**). Manual cell annotation was conducted based on published literature, and marker validation showed an ideal diagonal distribution, verifying the credibility of the annotation results (**Figure 14C**). In ccRCC, CRHBP was highly expressed in endothelial cells and dendritic cells (**Figure 14D**). In silico knockout of CRHBP resulted in only two differentially expressed genes (DEGs), namely SRGN and TYROBP (**Figures 14E, F**). GO enrichment analysis of CRHBP together with the two DEGs revealed significant changes in the biological process pathway negative regulation of cytokine production (**Figure 14G**). For GO cellular component terms, the enriched items were mainly cytolytic granule, secondary lysosome, and dense core granule (**Figure 14H**). For GO molecular function terms, the main enriched functions included peptide hormone binding and hormone binding (**Figure 14I**).

### 3.13 RT qPCR

In Homo sapiens, quantitative PCR analysis revealed a statistically significant decrease of CRHBP expression in KIRC samples compared to controls (P = 0.0043; **Figure 14J**), whereas UCN2 expression showed no significant differential trend in KIRC (P = 0.31; **Figure 14K**). Conversely, in mouse, CRHBP expression demonstrated no significant alteration in KIRC (P = 0.12; **Figure 14L**), while UCN2 exhibited a marked increase in KIRC tissues (P = 0.0043; **Figure 14M**).

## 4 Discussion

Our comprehensive analysis of KIRC identified MFSD4, CRHBP, and MPP7 as important DEGs and MDSGs, which play a key role in the activation of the RTK pathway (46). This study provides a multidimensional view of the KIRC genomic and immunological landscape using a variety of bioinformatics tools, including GEPIA 2, UALCAN, and TIMER. The findings suggest that CRHBP in particular can serve as a prognostic biomarker, with its expression level closely correlating with methylation patterns and clinical prognosis.

### 4.1 CRHBP’s role in immune modulation

The identified DEGs and MDSGs were not only underexpressed in KIRC, but also the expression level gradually decreased from stage 1 to 4, suggesting that they may play a role in disease progression. TIMER analysis showed that CRHBP was positively correlated with NKT cells and inversely correlated with methylation level, which further confirmed the relationship between CRHBP and improved prognosis. Single-cell sequencing data confirmed the high expression of CRHBP in specific immune cell types, such as MitoHigh Myeloid, TP1, and MitoHigh T-Helper. Meanwhile, the expression in FOLR2-Hi TAM and CXCL10-Hi TAM was significantly affected by ICB and sex. This suggests a complex interaction between gene expression, immune cell composition, and treatment response. Drug sensitivity analyses pointed to potential therapeutic avenues, with several drugs showing indirect interactions with CRHBP. The identification of these interactions through the STITCH database provides a basis for exploring the role of these drugs in KIRC therapy. The role of the RTK pathway activated by the identified DEGs and MDSGs is important as it is associated with cell growth, survival, and drug resistance, all of which are key factors in KIRC therapy. Furthermore, correlation analyses with immune cell infiltration provided by the TIMER and GSCA databases revealed immunological aspects of KIRC. The findings suggest that the tumour microenvironment is a dynamic landscape in which immune cells (e.g., NKT cells) play a crucial role in disease progression and therapeutic response. The effects of gender and ICB exposure on CRHBP expression and immune cell composition add another layer of complexity to the immunology of the disease. In addition to our study, a study by Wonbeak Yoo et al. (4) identified CRHBP as a novel multiple cancer biomarker associated with better prognosis and antitumour resistance. They found that the downregulation of CRHBP in most tumors was attributed to the upregulation of four miRNAs and was positively correlated with the number of CD8+ T cells, macrophages, and cancer-associated fibroblasts (CAFs). Their cell line experiments revealed that overexpression of CRHBP significantly inhibited cell proliferation in LUAD, LIHC, and KIRC cell lines, whereas inhibition of cell mobility was only found in KIRC and HCC cells (4). Their experimental results also corroborate that CRHBP is indeed capable of inhibiting tumour cell proliferation and migration in KIRC (4). Additionally, a study by Chen, Bangjie et al. revealed that CRHBP expression is significantly reduced in most tumor types and correlates with survival prognosis, immune checkpoint (ICP) markers, tumor mutational burden (TMB), and microsatellite instability (MSI) (47). The anticancer role of CRHBP in hepatocellular carcinoma (LIHC) was further confirmed through Western blotting, EdU staining, JC-1 staining, Transwell assays, and wound healing experiments (47). Future studies should integrate existing methodologies and findings to further elucidate the role of CRHBP in modulating the immune system within KIRC. The regulatory impact of UCN2 on CRHBP and its subsequent effects on immune responses also warrant further exploration.

### 4.2 UCN2-CRHBP functional axis

Possible Mechanisms of the UCN2-CRHBP Functional Axis: (1) Current evidence suggests a collaborative role between UCN2 and CRHBP under the influence of stress and hormones. Xu et al. identified the dynamic expression of the CRH/urocortin-receptor-binding protein (UCN-R-BP) system in the macaque corpus luteum, where it is modulated by luteinizing hormone (LH) during the menstrual cycle (48). This regulatory axis extends beyond the kidney. Stress adaptation mechanisms further highlight this axis, such as Kolasa et al. investigated the impact of chronic mild stress (CMS) on molecular stress markers. Rats subjected to 2-week CMS were stratified into three groups: stress-susceptible group (SSG), stress-non-reactive/resilient group (SNRG), and stress-invert-reactive group (SIRG). SSG exhibited elevated plasma UCN2, whereas SNRG and SIRG showed opposing behavioral effects but similar gene expression profiles (e.g., decreased pituitary Crh, Ucn2, and Ucn3expression with elevated plasma ACTH). This indicates a conserved feedback mechanism in stress responses (49). These findings imply that KIRC—acting as an endogenous stressor—may modulate the UCN2-CRHBP axis in a patient-specific manner, thereby influencing immune status. (2) Evolutionary conservation underscores its functional significance. Endsin et al. isolated cDNAs encoding three CRH family members, two CRHRs, and one CRHBP from Petromyzon marinus. Phylogenetic analysis revealed that two CRH peptides align with the CRH/urotensin-1 (UI) lineage, while another is an ortholog of urocortin 3 (Ucn3), suggesting close evolutionary ties between CRHBP and UCN2 (50). (3) The tissue-specific expression pattern further validates our hypothesis. Squillacioti et al. detected UCN, CRHR1, and CRHR2 (but not CRHBP) in equine thyroid tissue. UCN immunoreactivity (-IR) localized to follicular cells, CRHR2-IR to C-cells, and CRHR1-IR to vasculature, suggesting a UCN-centric paracrine system regulating thyroid physiology (51). Although direct evidence in testicular UCN2-KIRC interactions is lacking, the collective data confirm that hormone-tissue specificity modulates the UCN2-CRHBP axis. (4) Tezval et al. explicitly demonstrated that “cc-RCC is characterized by a significant loss of CRHBP mRNA expression, which correlates with more aggressive tumor phenotypes. Depletion of CRHBP protein further indicates its involvement in renal carcinogenesis as part of the UCN system” (52). Future studies will explore testicular UCN2 expression in KIRC immunity and prognosis, building on this mechanistic foundation.

The TIDE database indicates that the prognostic benefits associated with high CRHBP expression can be influenced by immune checkpoint blockade (ICB) therapy, but this benefit is not consistently stable, as evidenced by significant survival advantages observed in the ICB_Braun2020_PD1 cohort (**Figures 13A, C**) but absent in the ICB_Miao2018_ICB cohort (**Figures 13A, E**). Furthermore, results from single-cell RNA (scRNA) analyses correlating gene expression with 14 tumor cell states revealed that UCN2 exerts inhibitory effects on tumor angiogenesis and hypoxia (**Figure 13F**). This finding not only complements the tissue-specific risk effects of UCN2 observed in the TSMR dataset (**Figures 8A–H**) but also lays the groundwork for understanding UCN2’s mechanistic role in KIRC and renal tissue. Concurrently, this evidence strongly supports the notion that CRHBP alone cannot fully explain its impact on KIRC prognosis, therapeutic response, methylation levels, and cellular effects—such as contradictory methylation patterns across databases and inconsistent associations between NKT cell infiltration and prognosis. Future extensive experiments are required to validate the regulatory role of the CRHBP-UCN2 axis in KIRC, particularly concerning UCN2’s effects on tumor cells themselves and the tumor microenvironment, including tumor-infiltrating immune cells (**Figures 8I–O**).

### 4.3 Tetralateral assessment method for the drugability of target proteins

We screened candidate drugs based on correlations between network pharmacology (utilizing online servers such as CTRP, GDSC, and STITCH), gene expression levels, and FDA-approved drugs or those in clinical trials (via the CellMiner database), followed by molecular docking to further refine drugs that can tightly bind to target proteins; additionally, we treated the target protein as a polypeptide fragment to observe its potential to induce immune responses—this four-step process encompassing network pharmacology, clinical correlation analysis, molecular docking, and immune simulation for drug accessibility analysis of target proteins is termed TAM-DTP.

#### 4.3.1 OSI-930

OSI 930, a compound from MedChemExpress, has been extensively studied for its multifaceted roles in various biological contexts. Primarily, OSI 930 has been utilized as a test compound to evaluate its interaction with erlotinib and its potential as an aldehyde oxidase (AO) substrate (53). *In vitro* assays have demonstrated its significance in understanding the blood-to-plasma ratio and free fraction in human plasma, providing critical insights into the drug-drug interaction (DDI) potential of erlotinib with AO substrates (53). This information is vital for assessing clinical risks associated with DDIs and for validating physiologically based pharmacokinetic (PBPK) models. Additionally, OSI 930 has been implicated in exploring resistance mechanisms in KRAS-mutant lung cancer cells, particularly concerning PP2A modulation (54). This research underscores the necessity for a comprehensive understanding of kinase inhibitors and their interactions with phosphatases, suggesting potential avenues for targeted therapy combinations to overcome drug resistance (54). Furthermore, OSI 930 has been investigated for its effects on circadian oscillations and metabolic rhythms in mice. The compound’s ability to enhance CaMKIIδ activity has been shown to improve circadian amplitude, aligning with the study’s goal of identifying interventions for circadian-related disorders (55). Unfortunately, however, the study by Yap, Timothy A et al. (56) showed that OSI 930 has high toxicity, and since the publication of this literature in 2013, there have been no subsequent reports of Phase II/III studies, and it has not been approved by any regulatory agency. Combining its toxicity characteristics and the alternative use of similar drugs (such as sunitinib), it can be inferred that its development has been terminated; hence, it has been excluded as a candidate drug.

#### 4.3.2 Dabrafenib

A Phase III randomized controlled trial conducted by Hauschild, Axel et al. demonstrated that dabrafenib significantly improved progression-free survival (PFS) compared to dacarbazine in patients with BRAF(V600E) mutation-positive melanoma, with a median PFS of 5.1 months for dabrafenib (n=187) versus 2.7 months for dacarbazine (n=63), yielding a hazard ratio (HR) of 0.30 (95% CI 0.18–0.51; p<0.0001) (57). Treatment-related adverse events of grade 2 or higher occurred in 53% (100/187) of patients receiving dabrafenib and 44% (26/59) of those receiving dacarbazine (57). Concurrently, our molecular docking results indicate that dabrafenib exhibits binding affinities of -11.55 kcal/mol with CRHBP and -17.23 kcal/mol with UCN2 (**Figure 11B**). Although no literature currently reports the therapeutic efficacy of dabrafenib for KIRC, our findings support future experimental investigations into dabrafenib’s potential therapeutic role in KIRC.

#### 4.3.3 As a peptide marker

Our findings also demonstrated that two proteins were able to induce high levels of IFN-γ, IL-2, active macrophages, memory Th cells, and Th1 cells at 35 days after three immunizations (**Figure 12**), indicating that the two proteins we screened (UCN2 and CRHBP) indeed elicit robust immune responses under in silico simulation conditions. Meanwhile, CRHBP expression decreased with advancing disease stage (**Figure 12A**), while UCN2 expression increased with stage progression (**Figure 12B**); from a progression perspective, this opposing trend not only suggests that UCN2 may potentially regulate CRHBP but also highlights the distinct immune responses induced by each protein, thereby prompting us to consider future therapeutic strategies where UCN2 and CRHBP could be applied separately as agonists and inhibitors to form a dual therapeutic approach for KIRC. Additionally, both proteins could serve as qualified tumor antigens for developing epitope peptide vaccines, aiming to activate the endogenous immune responses of KIRC patients for therapeutic purposes through tumor vaccination.

### 4.4 Strengths and limitations

The strength of our study lies in its integrated approach, which combines genomic, transcriptomic, and immunological data to paint a full picture of KIRC. The use of advanced analytical tools and the incorporation of single-cell sequencing data gave us a nuanced understanding of immune cell-specific gene expression patterns. However, there are some shortcomings in this study.

#### 4.4.1 Design deficient

Our reliance on bioinformatics tools means that our findings are relevant and need to be verified through experiments. The universality of our findings to other patient groups and the impact of other clinical variables on the study results remain to be determined. At the same time, the results of NKT cell immune infiltration in TIMER and GSCA are contradictory (the correlation between CRHBP expression levels and NKT cell content is negatively correlated in TIMER2.0 (**Figure 5A**) (r = -0.4), but the correlation between CRHBP expression levels and NKT cell content is positively correlated in GSCA (**Figure 5G**) (r = 0.13)), indicating that there may be underlying factors interfering with the impact of NKT cells on gene expression or clinical prognosis, such as the complexity of the immune microenvironment in different patients.

#### 4.4.2 Discrepancies exist in the promoter methylation levels of CRHBP in KIRC across the GSCA and UALCAN databases

UALCAN indicates hypomethylation of CRHBP in KIRC, whereas GSCA reports hypermethylation. Results from MethHC 2.0—a third database focusing on methylation—suggest that the differential methylation status of CRHBP in KIRC has limited prognostic impact (HR = 0.2068 [-3.278, 0.7008], P = 0.2812; **Figure 4A**). We hypothesize that these inconsistencies may arise from differences in sample inclusion criteria and methodologies for batch-effect removal across databases. To resolve such contradictions, future studies should: 1. Define unified promoter regions for methylation analysis of specific genes in distinct cancer types. 2. Systematically compare the accuracy of different batch-effect correction methods. 3. Establish standardized protocols to ensure consistency in methylation profiling. Adopting these measures will enhance the reliability and comparability of epigenetic studies in oncology.

#### 4.4.3 Triangulation verification method using the IIA-scRNA-TSMR (Immune Infiltration Algorithm scRNA TSMR)

Although we innovated the immune cell TVM using IIA-scRNA-TSMR, the immune cells found were not entirely consistent, and ultimately, we could only conclude that T cells were hub cells. The integration of bulk-tissue algorithms (Gepia2/GSCA), scRNA-seq, and causal inference (TSMR) mitigates limitations inherent to any single method. scRNA-seq identifies context-specific cell states (e.g., TP1 subset), while TSMR prioritizes causal immune phenotypes (6/11 cell types passing MR thresholds), enabling functional prioritization beyond correlation. Bulk-level findings (e.g., CD4+ memory T activation and CD4+ Th2 via Gepia2) align with single-cell data (MitoHigh T-Helper), confirming T-cell-driven immune responses despite heterogeneity.

#### 4.4.4 Limitations

##### 4.4.4.1 Technical discrepancies still require explanation

Inherent methodological biases lead to inconsistent cell type identification, resulting in deconvolution algorithms (Gepia2/GSCA) reporting “neutrophils/NKT” based on marker genes, while scRNA highlights clustering based on mitochondrial activity (MitoHigh subpopulations). TSMR identifies broad protective populations (CD33dim HLA DR+), while scRNA resolves more refined metabolic states.

##### 4.4.4.2 Data integration challenges

Consistency analysis requires specialized statistical frameworks (e.g., effect size standardization) to resolve conflicts, such as the fact that only one immune cell type overlaps between different cohorts (TSMR shows n=6 in cohort 1 and n=11 in cohort 2). Batch effects across batch/single-cell/genomic datasets also need to be addressed, which is critical for resolving contradictions in immune infiltration algorithm results, methylation level discrepancies, and other issues.

##### 4.4.4.3 Causal inference limitations in TSMR

Level pleiotropy may confound MR results, even after SNP filtering (nsnp > 3) and multiple pleiotropy tests and sensitivity tests. Moreover, protective effects (e.g., HLA DR+ CD11b+) still require experimental validation to rule out tumor education artifacts (58).

##### 4.4.4.4 The fundamental discrepancy in cellular origin poses significant challenges

(1) Reference profile mismatch – TSMR analysis relies on 731 immune cell signatures derived from peripheral blood, while IIA and scRNA analyses are based on transcriptional profiles from tumor tissues, creating a disconnect between circulating immune references and tissue-specific tumor microenvironment (TME) contexts; (2) Activation state blindness – peripheral blood signatures fail to capture tissue-resident immune cell states (e.g., tumor-educated macrophages or exhausted T cells) leading to misinterpretation of causal relationships identified by TSMR; (3) Developmental context gap – blood-derived signatures cannot reflect local differentiation trajectories within tumors (like tumor-associated neutrophil polarization or T-cell anergy), potentially misattributing causal effects to circulating precursors rather than tissue-adapted effectors. This limitation might compromise the biological plausibility of inferred causal chains, as peripheral blood immunomes may not faithfully represent the functional states or abundances of tumor-infiltrating immune subsets, risking erroneous conclusions about mechanistic drivers within the TME (58).

##### 4.4.4.5 Tumor subclonal heterogeneity or functional polarization

(1) Spatial-temporal dynamics – distinct tumor subclones drive localized immune microenvironments (e.g., immunosuppressive niches in hypoxic ccB regions) that evade detection by bulk-level methods like TSMR or IIA; (2) Context-dependent plasticity – polarized immune states (like N2 neutrophils) exhibit reversible functional switching upon microenvironmental cues (TGF-β/IFN-γ gradients (59, 60)), causing transient causal signals that violate MR’s temporal stability assumption; (3) Masked effector functions – antagonistic subpopulations (e.g., cytotoxic ccA-infiltrating CD8+ T cells vs. regulatory T cells in ccB) may cancel out net causal effects in aggregated analyses, artificially flattening TVM-detected associations despite biologically significant localized activity. These spatially structured and dynamically shifting immune-tumor interactions fundamentally challenge the static, population-averaged causal models underpinning TSMR-TVMs, necessitating spatially resolved single-cell multi-omics to deconvolute context-specific causality.

## 5 Conclusions

The role of CRHBP in KIRC cannot be overlooked, and its effects are unstable under the influence of ICB, potentially related to the regulation of the immune microenvironment, immune infiltration levels, and UCN2. Future research should focus on (1) validating the mechanism of the CRHBP-UCN2 axis in KIRC, (2) conducting an in-depth analysis of the impact of T cells, particularly HLA DR on CD33dim HLA DR+ CD11b+ cells, on KIRC, (3) validate the efficacy of dabrafenib in KIRC.

## Data Availability

The original contributions presented in the study are included in the article/**Supplementary Material**. Further inquiries can be directed to the corresponding authors.

## Supplementary material

Supplementary Table 1 DEG and MSDG original data.

Supplementary Table 2 TIMER and GSVA original data.

Supplementary Table 3 Single cell portal searching original data.

Supplementary Table 4 Single cell portal original data.

Supplementary Table 5 Drug sensitivity analysis.

Supplementary Table 6 Raw results of genetic TSMR primary results.

Supplementary Table 7 Raw results of genetic single-SNP results.

Supplementary Table 8 Raw results of all intersections.

Supplementary Table 9 Raw results of immune cell TSMR primary results.

Supplementary Table 10 Raw results of immune cell single-SNP results.

Supplementary Table 11 Raw results of immune cell Leave-one-out results.

Supplementary Table 12 Original enrichment data.

Supplementary Table 13 Detailed docking results.

Supplementary Table 14 TIDE original data.

Supplementary Material 1 Quantitative real-time PCR (qRT-PCR) detailed information.

Supplementary Material 2 Original protein expression figure.
