# Supplementary material for: Multi-omics analysis of kidney renal cell carcinoma in silico with preliminary in vivo validation
Source: Front Immunol. 2026 Jun 8;17:1732965. doi: 10.3389/fimmu.2026.1732965 (PMC13284111; doi:10.3389/fimmu.2026.1732965)
Supplement: Supplementary Material 1 — Quantitative real-time PCR (qRT-PCR) detailed information. [file DataSheet1.docx]

Quantitative real-time PCR (qRT-PCR)

Total RNA was isolated with TRIzol reagent (Invitrogen, Carlsbad, CA) according to the manufacturer’s instructions. The mRNA levels were determined by qRT-PCR analysis on an Applied Biosystems 7500 system (Applied Biosystems, Foster City, CA). The list of primers is presented in Table.

Primary renal tubular epithelial cells from mice were isolated as previously described [1]. Human Kidney 2, HK2, was purchased from Wuhan Procell System. (Wuhan, China) and cultured with MEM medium supplemented with 10% fetal bovine serum.

Human Kidney Carcinoma Cell Line A-498, A-498 cell, was purchased from Wuhan Procell System. (Wuhan, China) and cultured with MEM medium supplemented with 10% fetal bovine serum.

Renca was purchased from Wuhan Procell System. (Wuhan, China) and cultured with RPMI-1640+10% FBS +0.1 mM NEAA +1 mM Sodium Pyruvate +2 mM L-glutamine.

| Primer | 5’ to 3’ |
| --- | --- |
| CRHBP Hom-F1 | CACACCAGCATCGAAACTGC |
| CRHBP Hom-R1 | TGAAGACCATTTACGTGTCCCA |
| CRHBP Mus-F1 | ATGTCACCGAACTTCAAACTCC |
| CRHBP Mus-R1 | TTCTTGCACCTCTAGGTAGCG |
| UCN2 Hom-F1 | CGCGCATTGTCCTATCGCT |
| UCN2 Hom-R1 | CGGGCTTGCTCCAGTAAGA |
| UCN2 Mus-F1 | TTCGTGGTCCTGATGTTGGAT |
| UCN2 Mus-R1 | GTCACAGAGCTAGGAGTTGTCT |

|  | HK2 | A-498 |
| --- | --- | --- |
| CRHBP-hom | 8.588176401 | 3.821335548 |
| CRHBP-hom | 8.220293894 | 2.139827887 |
| CRHBP-hom | 9.44582699 | 4.375930171 |
| UCN2- hom | 2.615292137 | 2.359192809 |
| UCN2- hom | 2.566272081 | 1.950934537 |
| UCN2- hom | 2.132807925 | 2.381322516 |

|  | PTC | Renca |
| --- | --- | --- |
| CRHBP-mus | 0.762683446 | 0.567428444 |
| CRHBP- mus | 0.971363266 | 0.051844243 |
| CRHBP- mus | 1.349814236 | 0.417042405 |
| UCN2- mus | 1.125932985 | 2.361230412 |
| UCN2- mus | 0.789360165 | 2.464301223 |
| UCN2- mus | 1.125154734 | 3.225192106 |

[1] Fu H, Zhou D, Zhu H, Liao J, Lin L, Hong X, et al. Matrix metalloproteinase-7 protects against acute kidney injury by priming renal tubules for survival and regeneration. Kidney Int. 2019; 95: 1167-80.

First qPCR results

|  | HK2 | A-498 |
| --- | --- | --- |
| CRHBP-hom | 1.024470931 | 0.241465587 |
| CRHBP-hom | 1.479657338 | 0.094830636 |
| CRHBP-hom | 0.659688947 | 0.224084204 |
| UCN2- hom | 1.100020679 | 0.91804685 |
| UCN2- hom | 0.974890111 | 0.943464268 |
| UCN2- hom | 0.932488502 | 0.933349407 |

|  | PTC | Renca |
| --- | --- | --- |
| CRHBP-mus | 70.96701793 | 2.628493645 |
| CRHBP- mus | 16.57479242 | 45.12502941 |
| CRHBP- mus | 69.72343363 | 8.147784808 |
| UCN2- mus | 204.258709 | 685.3571353 |
| UCN2- mus | 257.3387329 | 745.5684028 |
| UCN2- mus | 291.5465284 | 706.1615561 |
