# Supplementary material for: Multi-omics analysis of kidney renal cell carcinoma in silico with preliminary in vivo validation
Source: Front Immunol. 2026 Jun 8;17:1732965. doi: 10.3389/fimmu.2026.1732965 (PMC13284111; doi:10.3389/fimmu.2026.1732965)

Figure 5A:

<https://www.proteinatlas.org/ENSG00000174514-MFSD4A/cancer/renal+cancer#cptac_renal_cell_carcinoma>


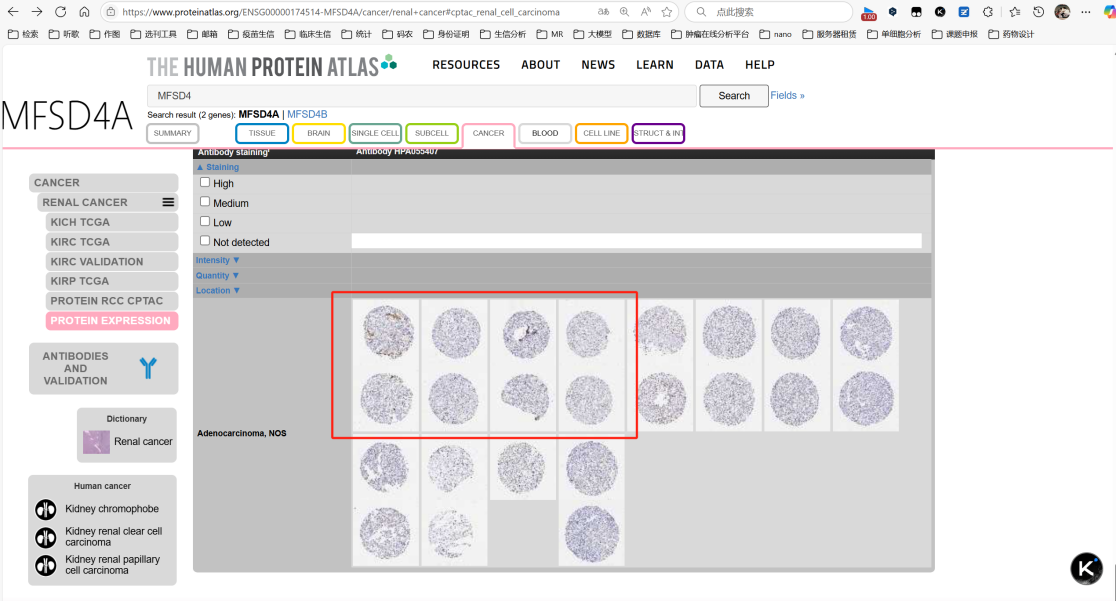


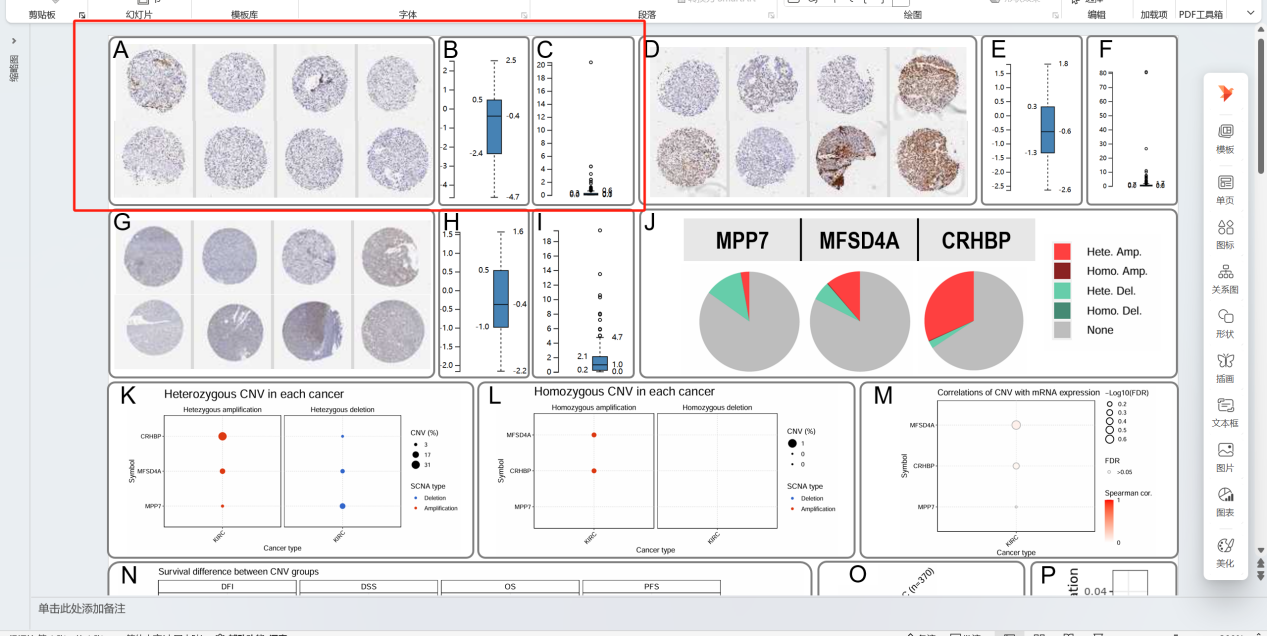


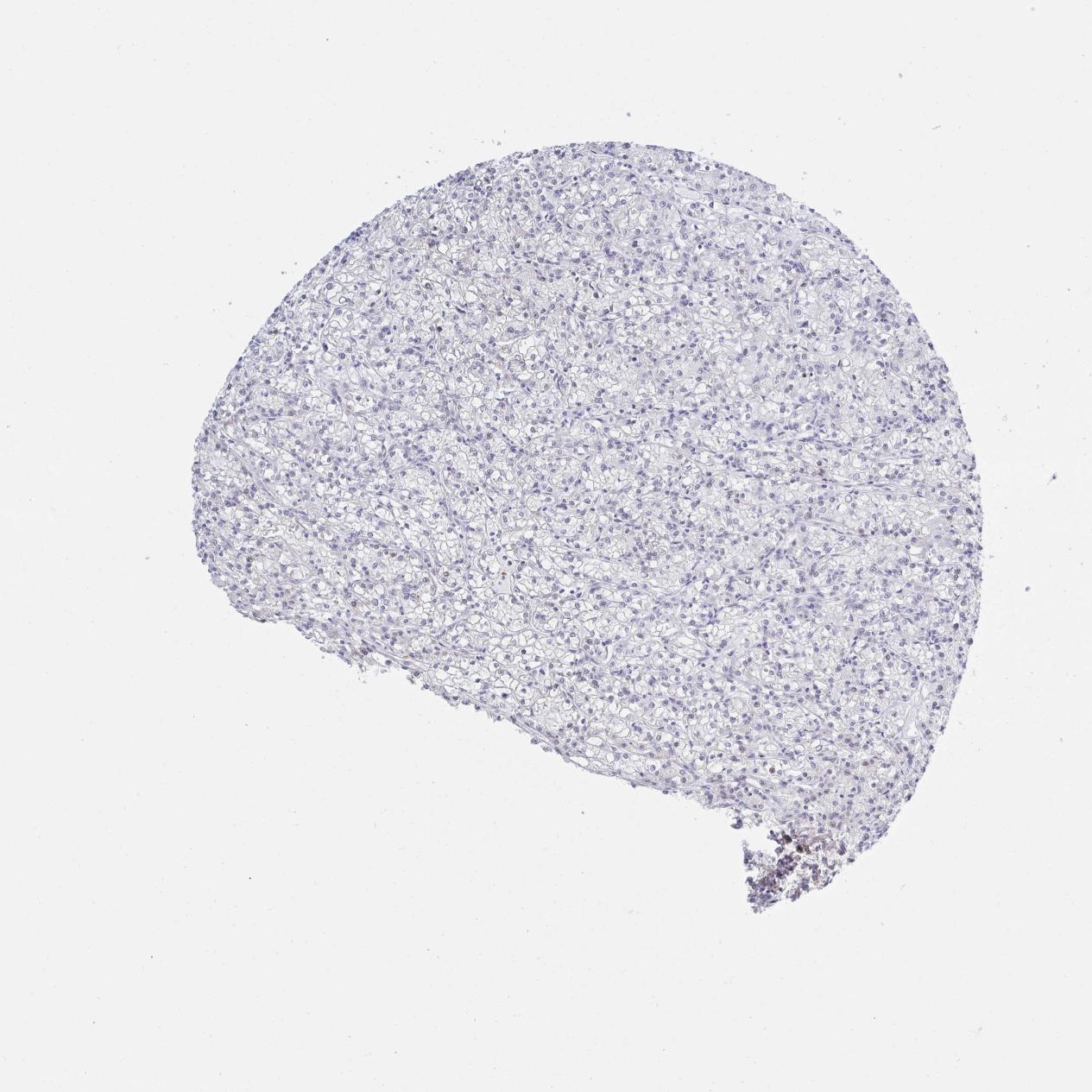

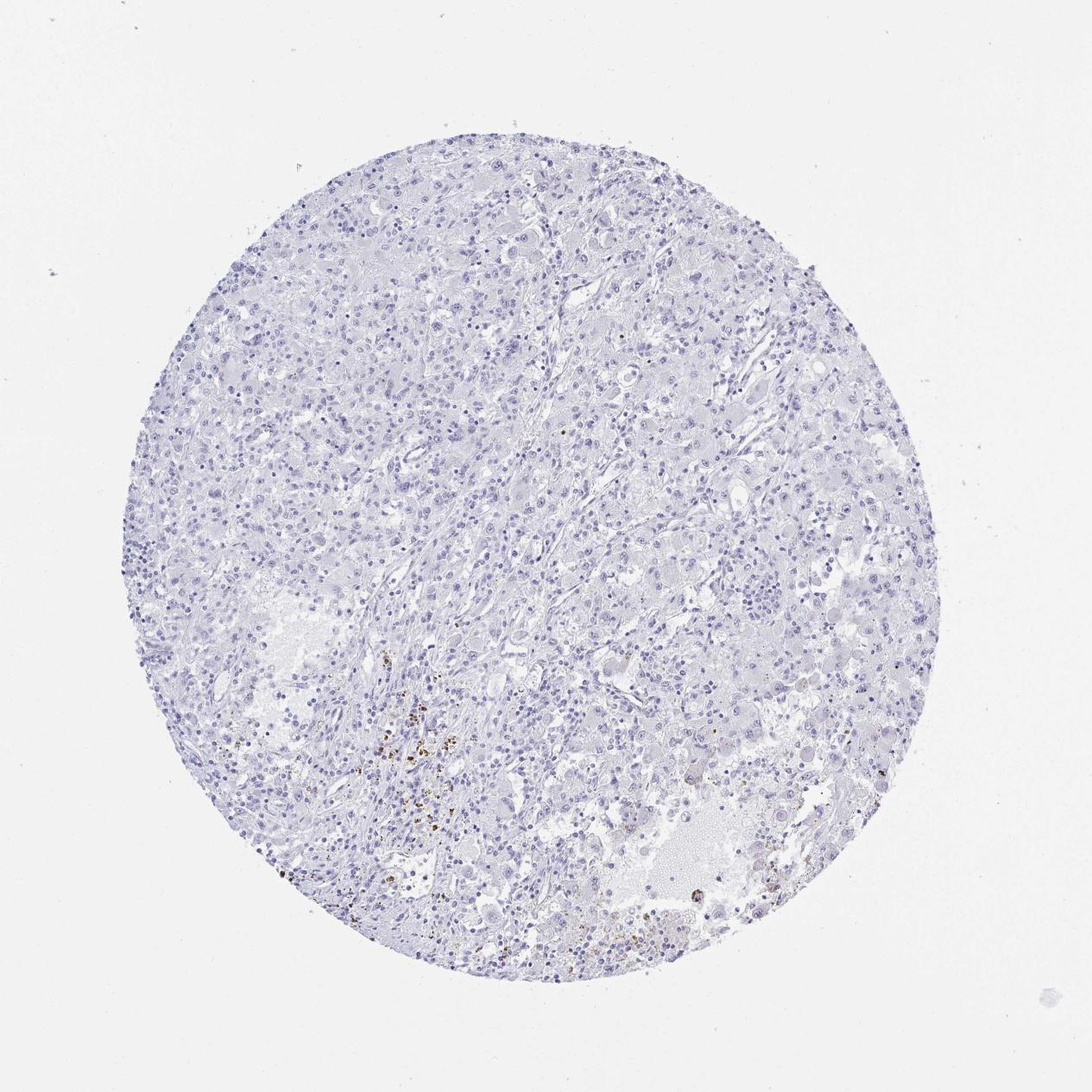

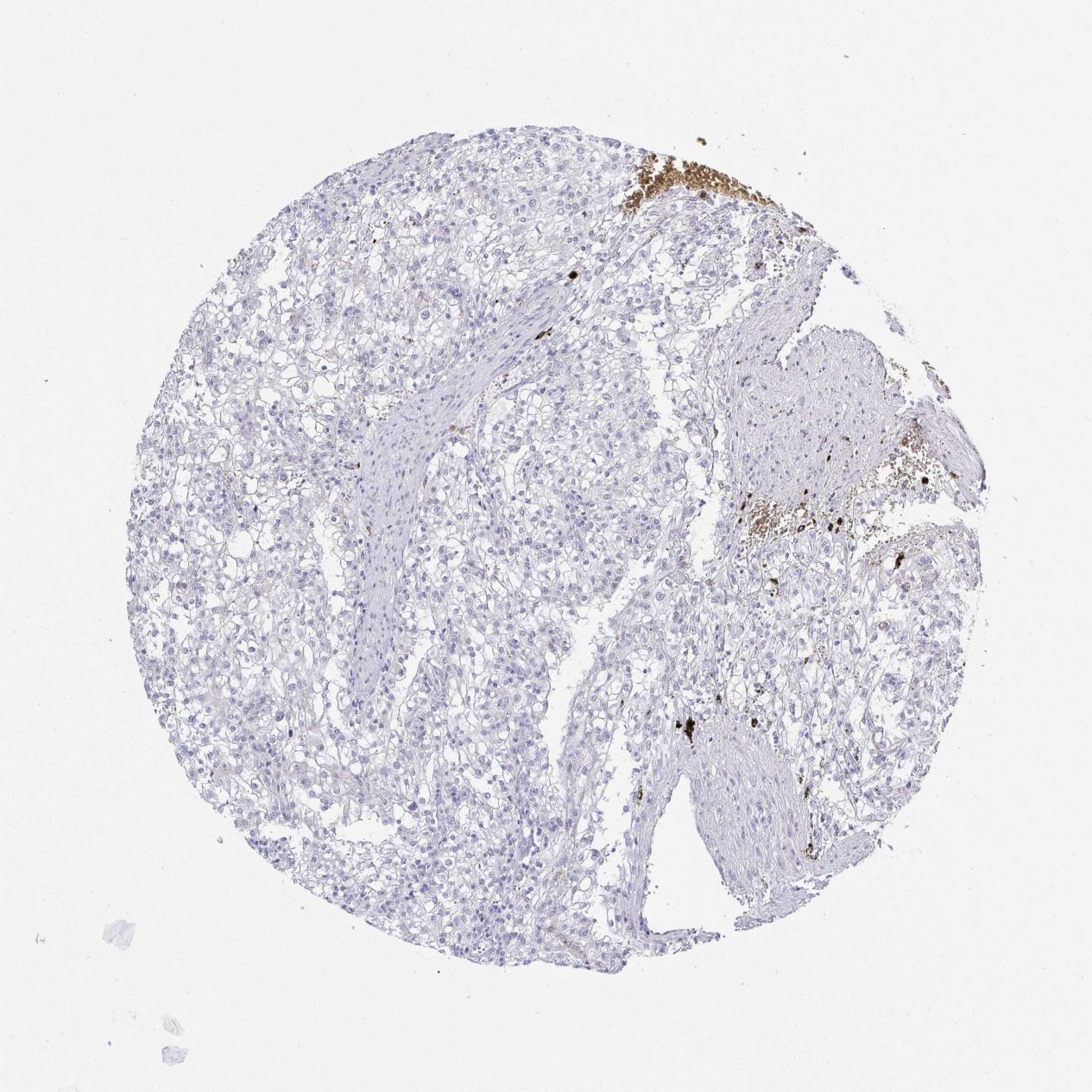

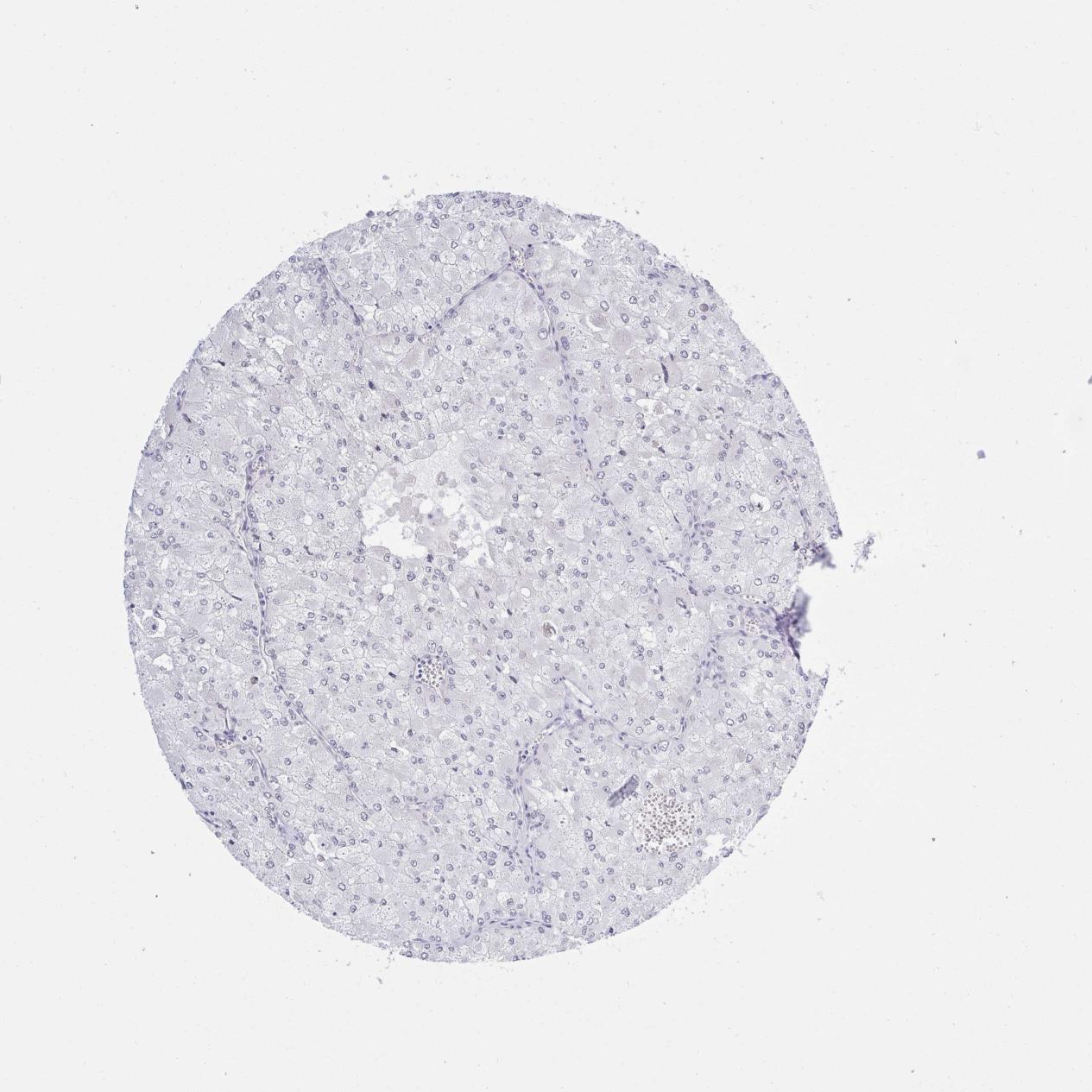

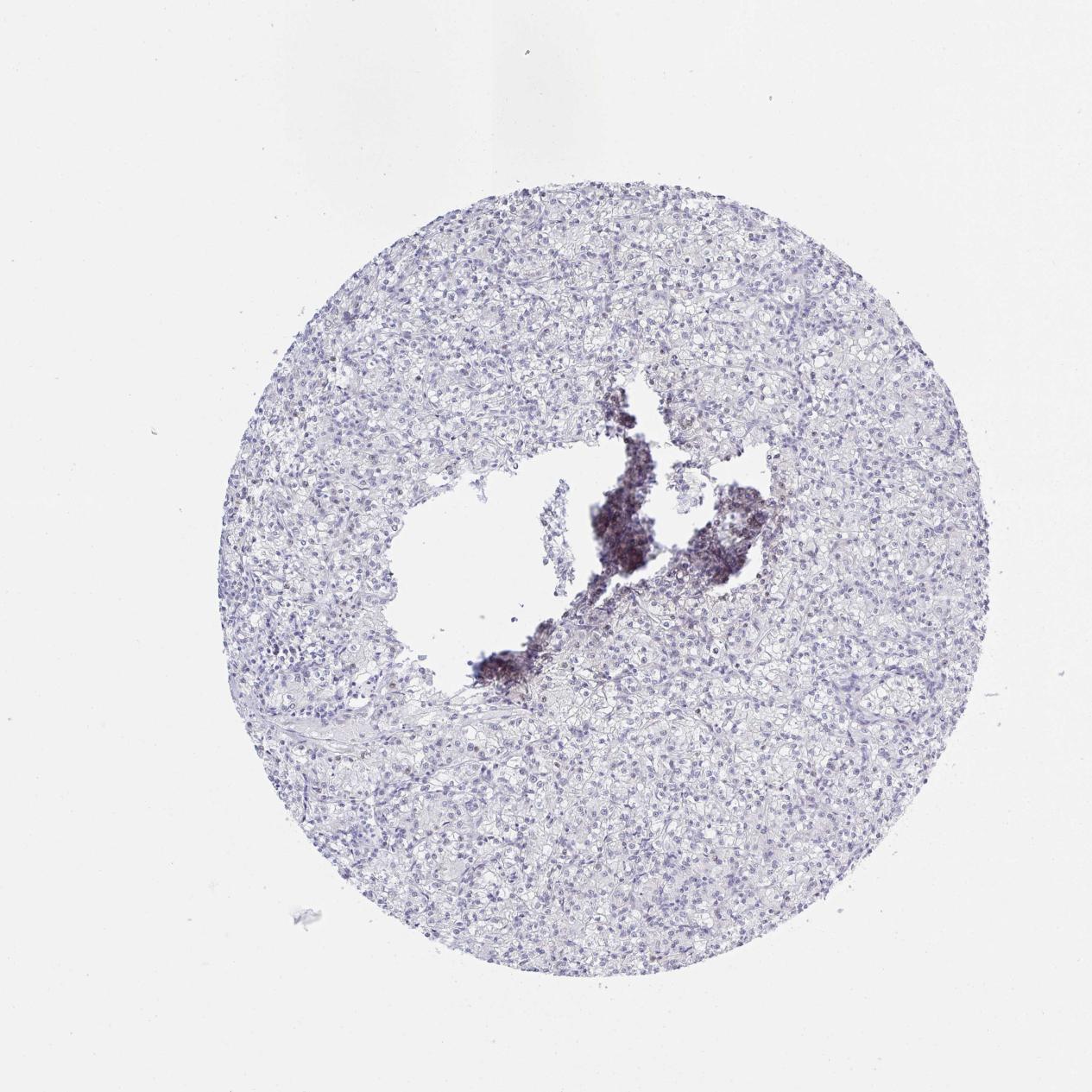

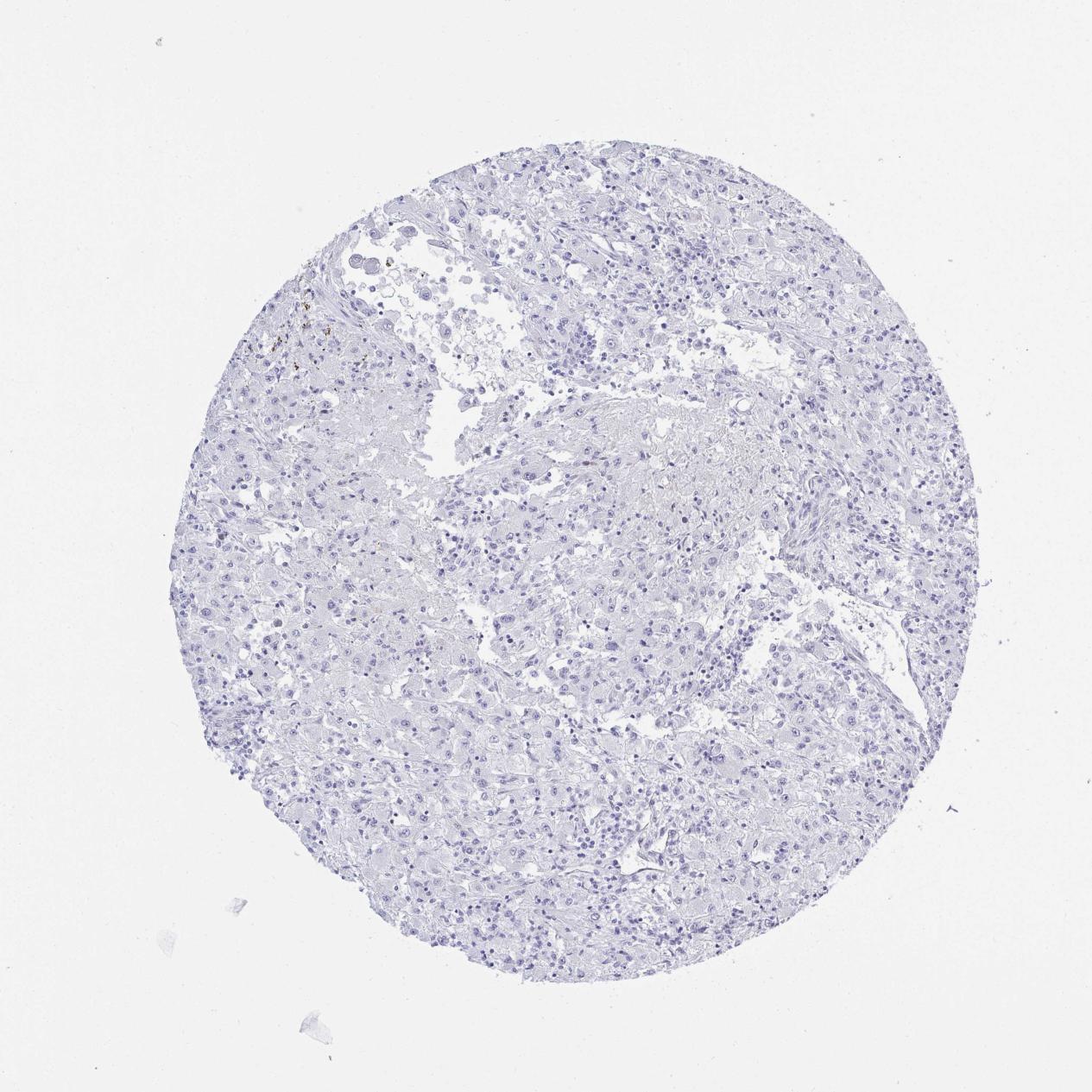

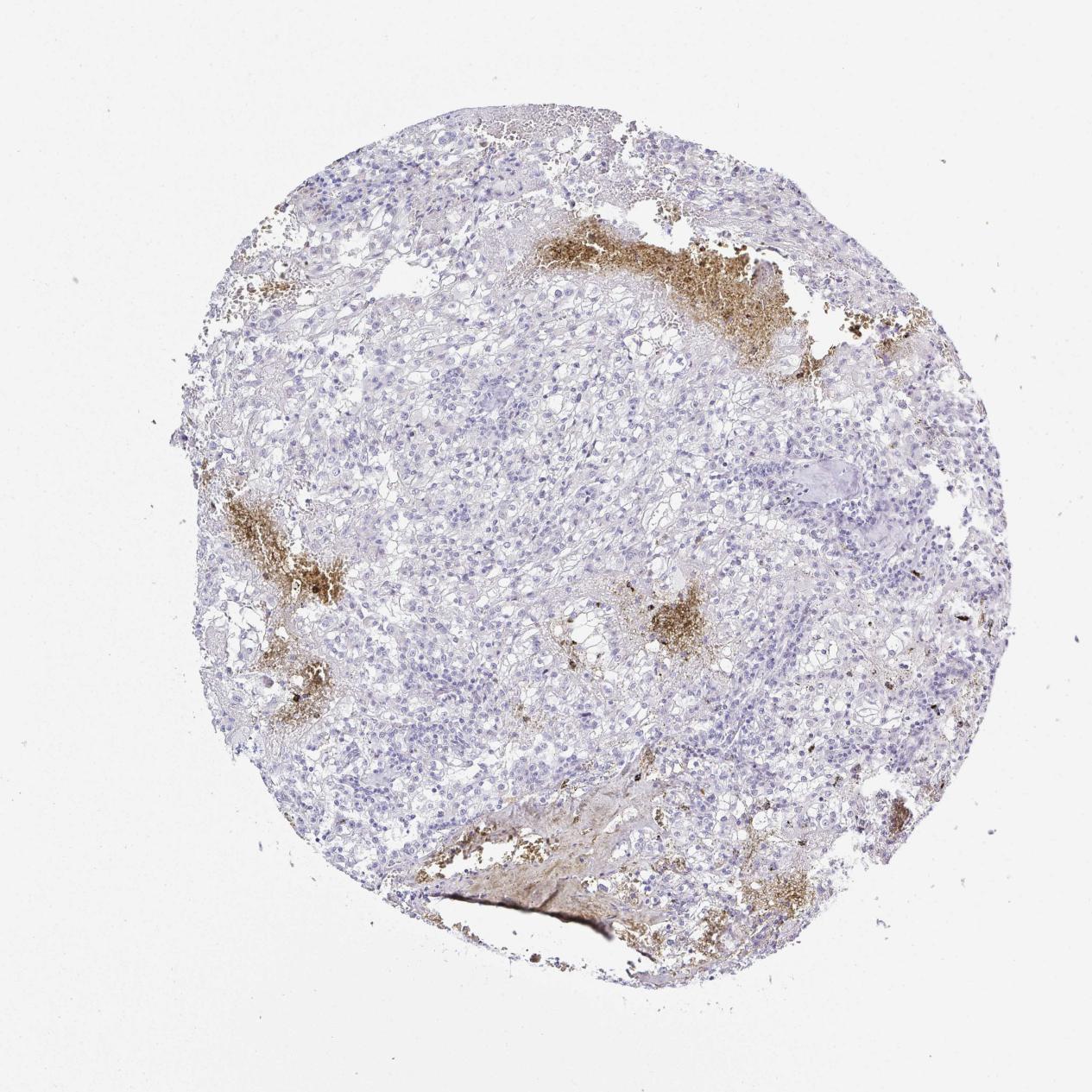

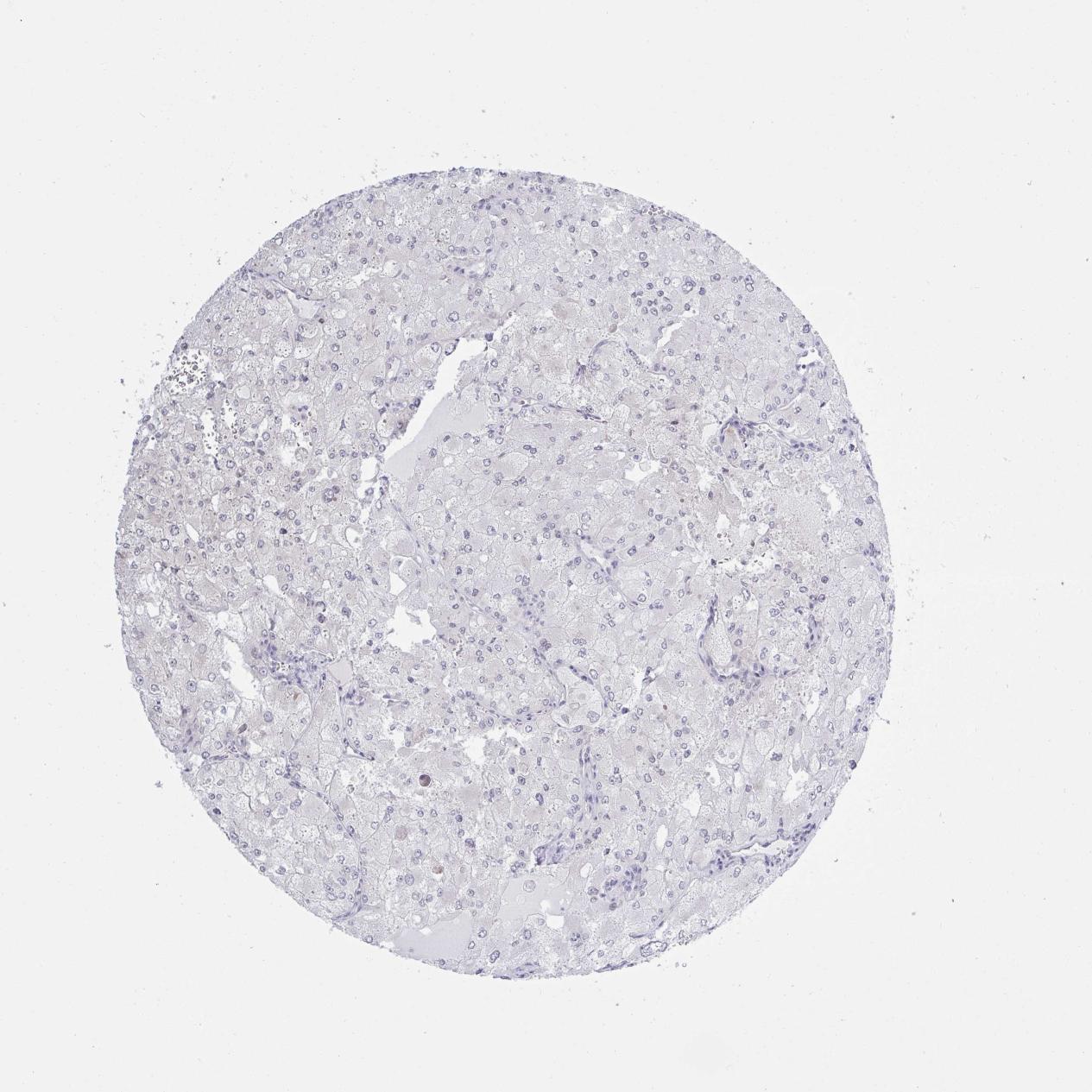


Figure 5D:

<https://www.proteinatlas.org/ENSG00000145708-CRHBP/cancer/renal+cancer#KICH_TCGA>


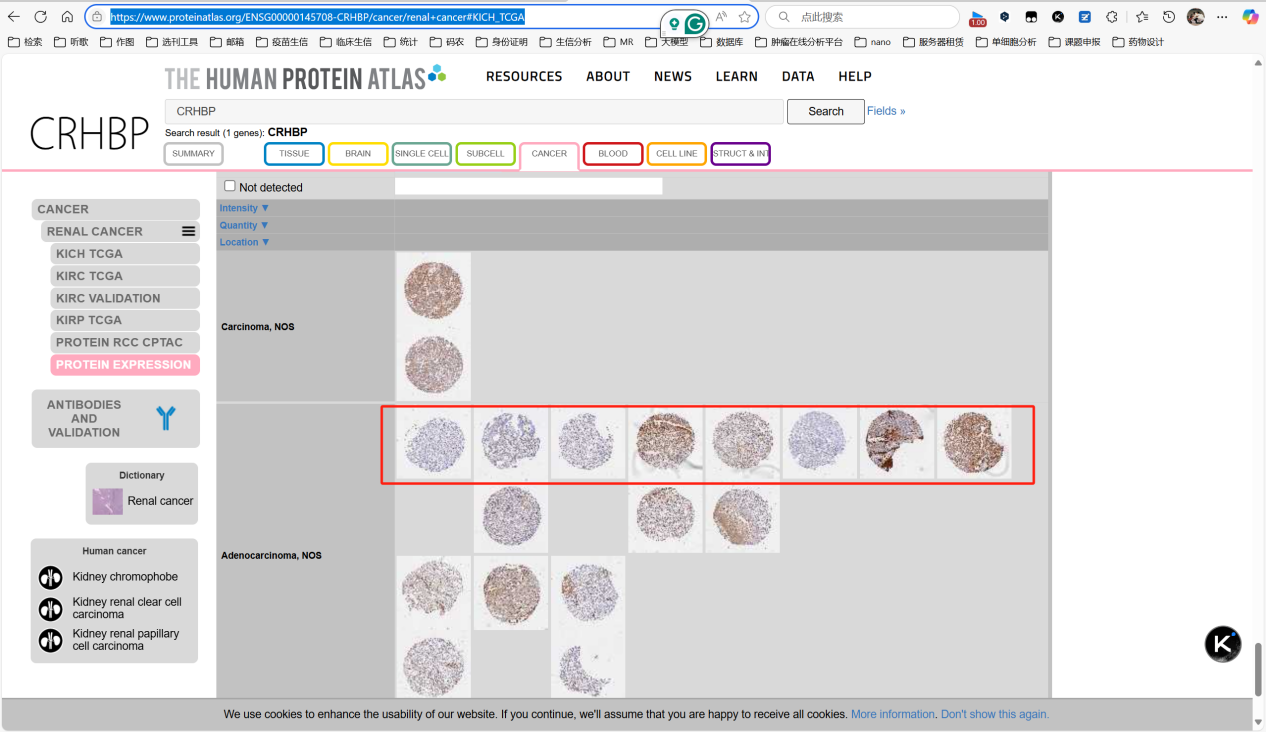


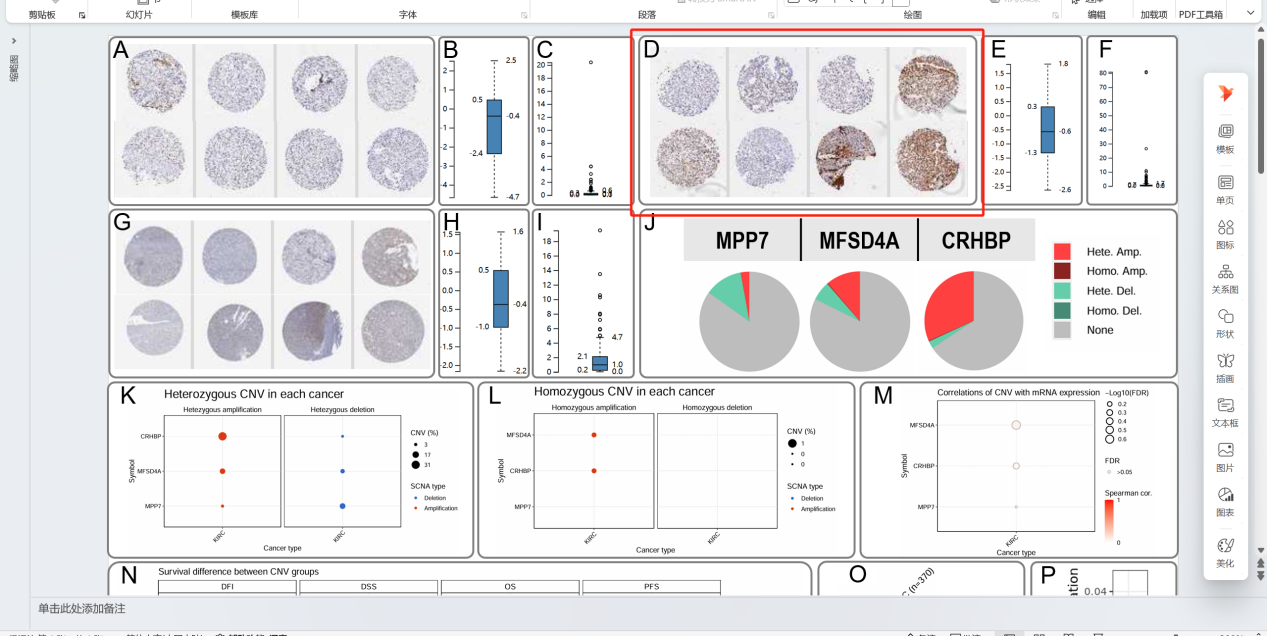


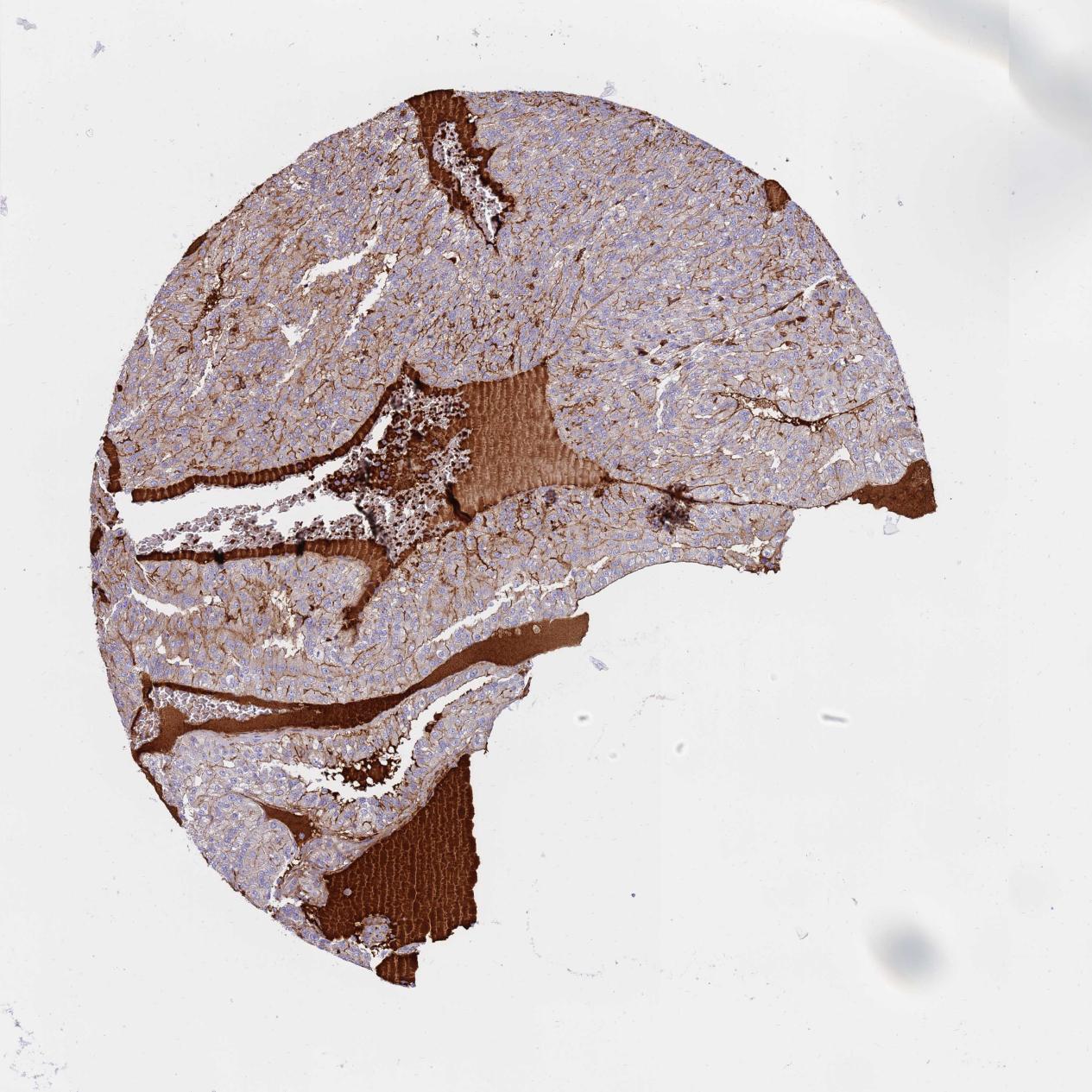

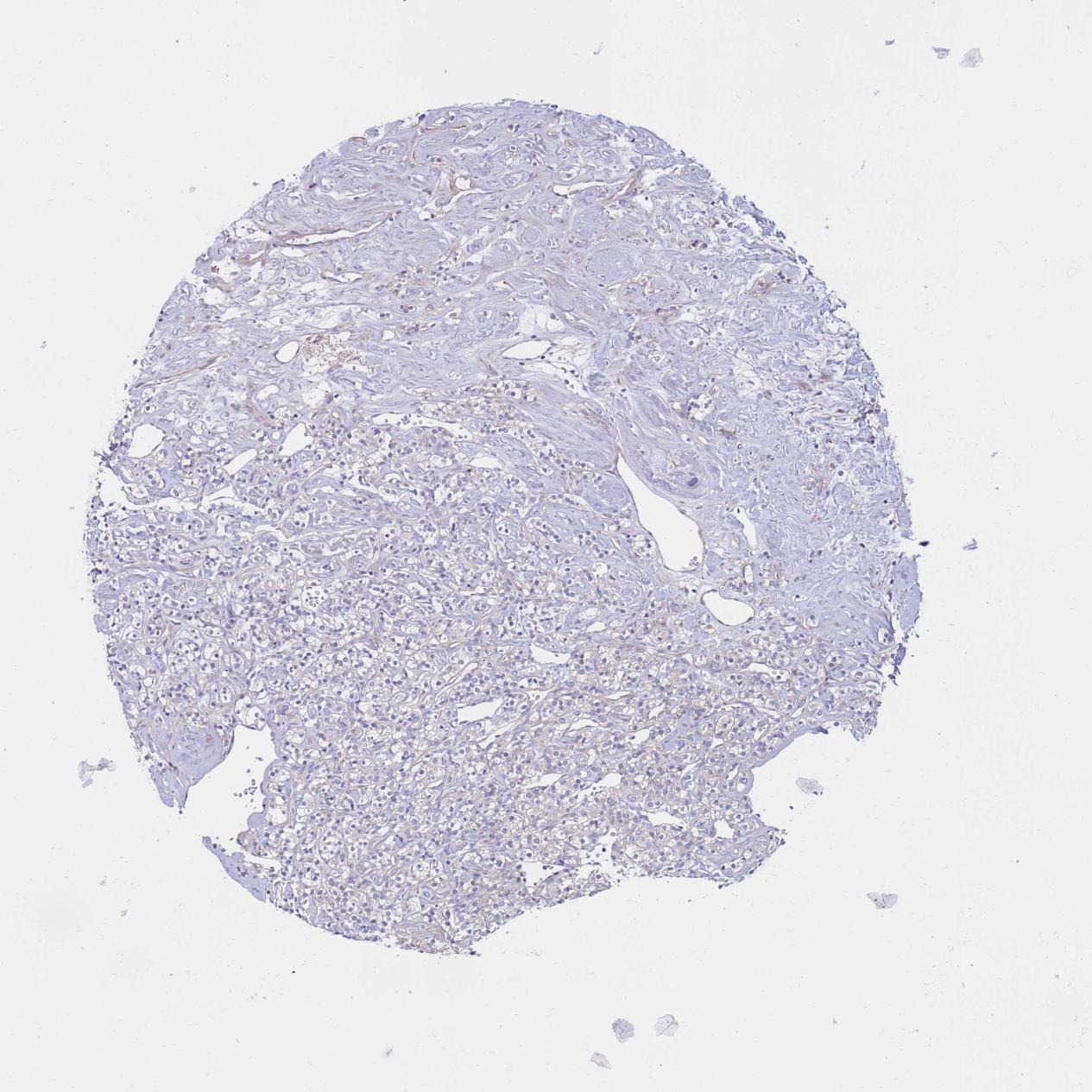

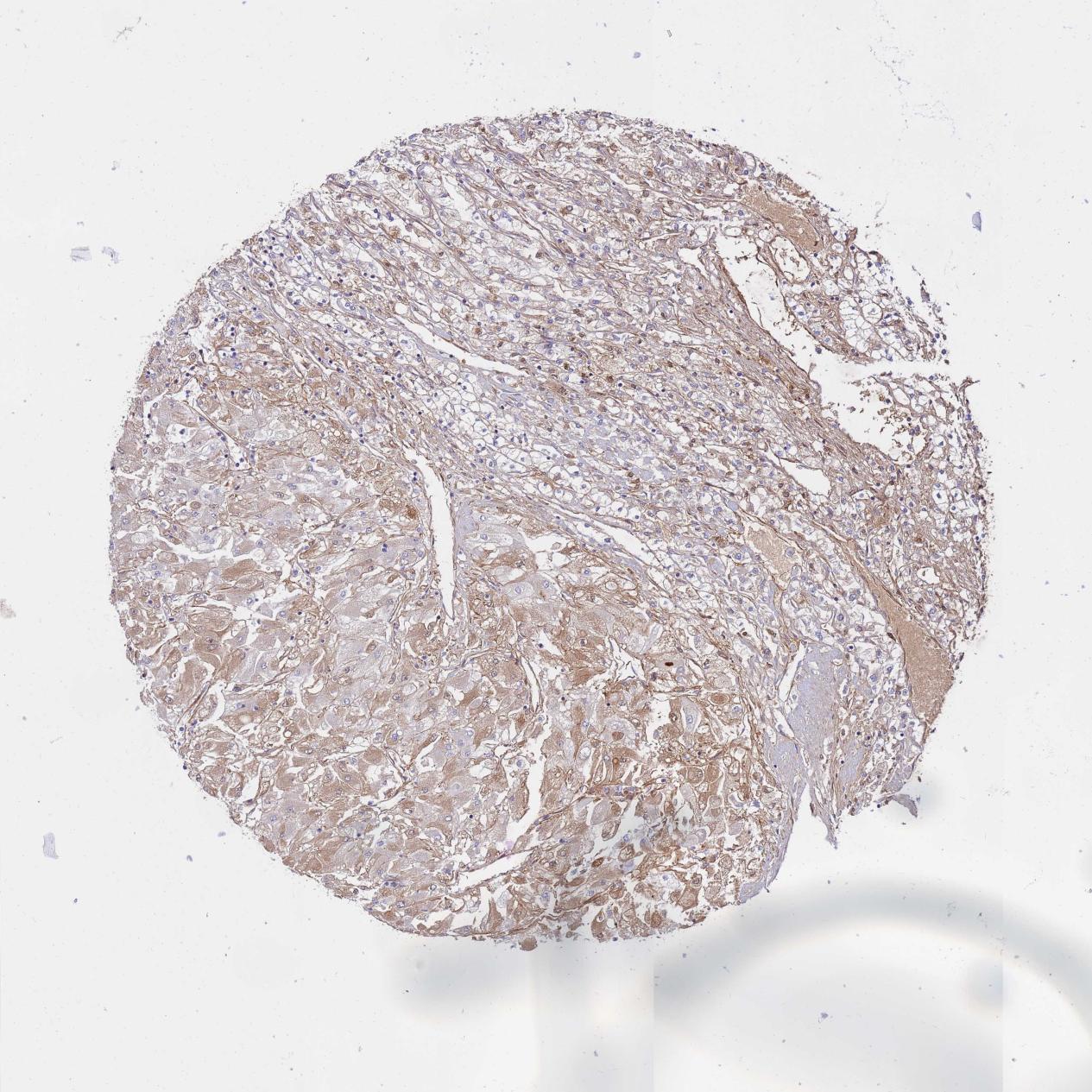

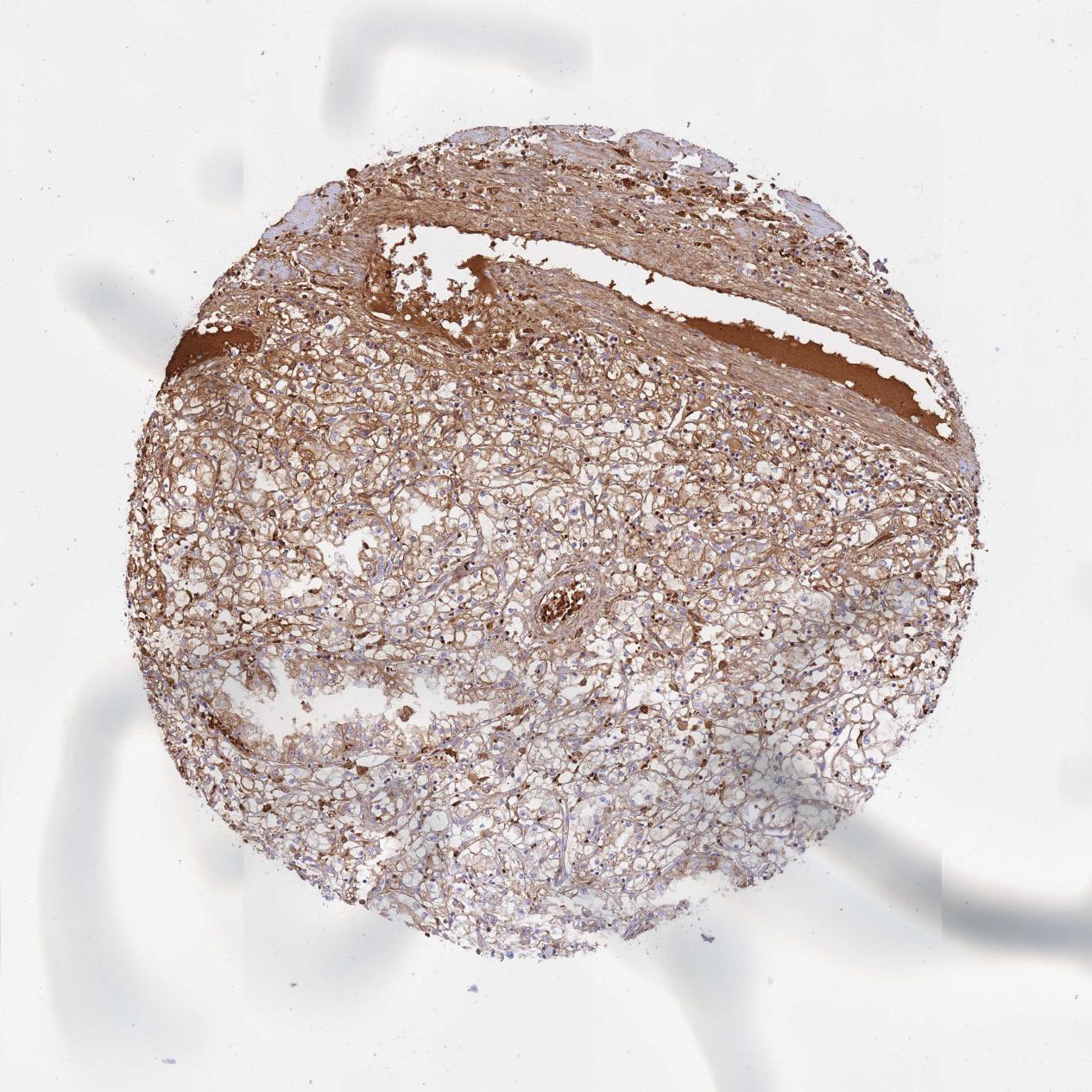

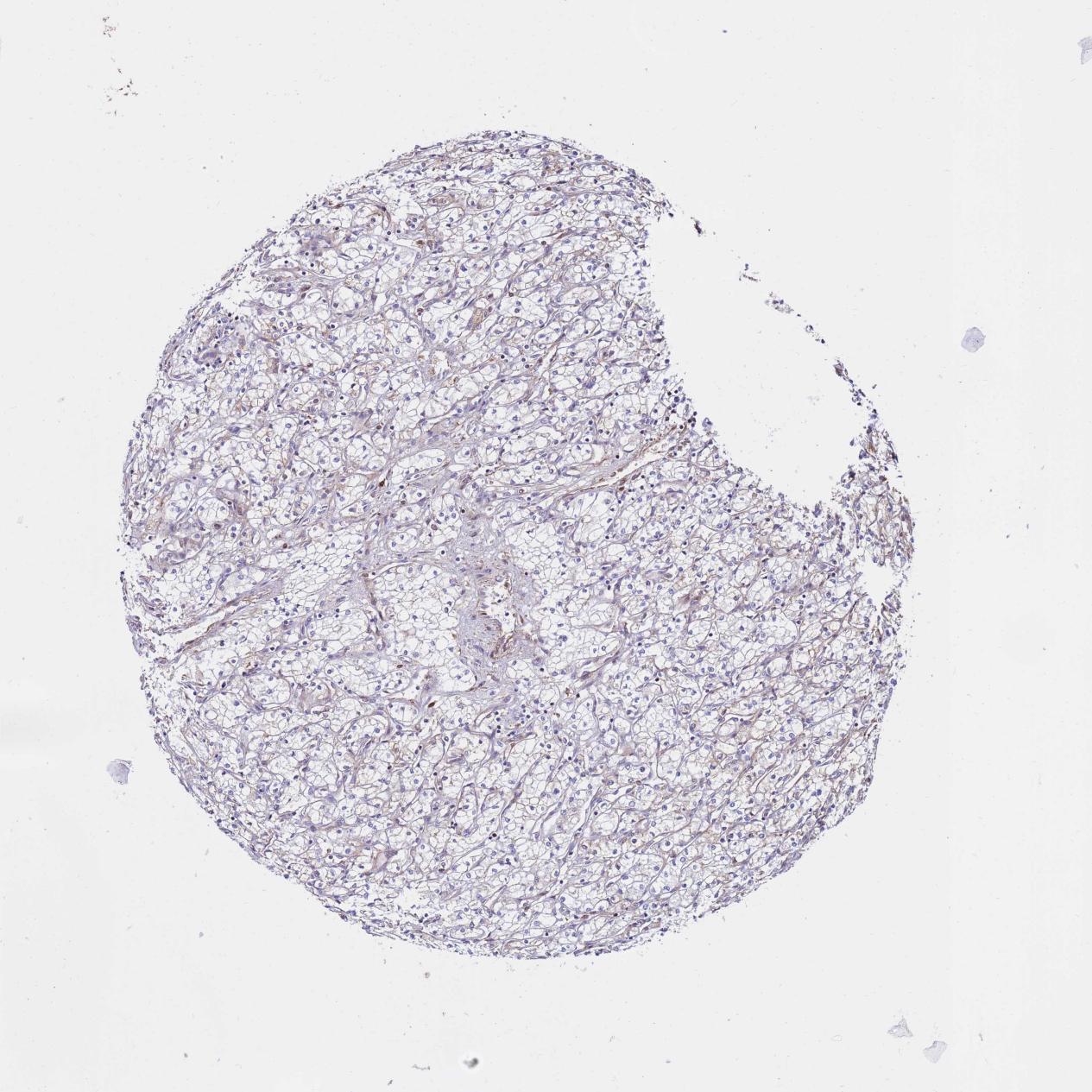

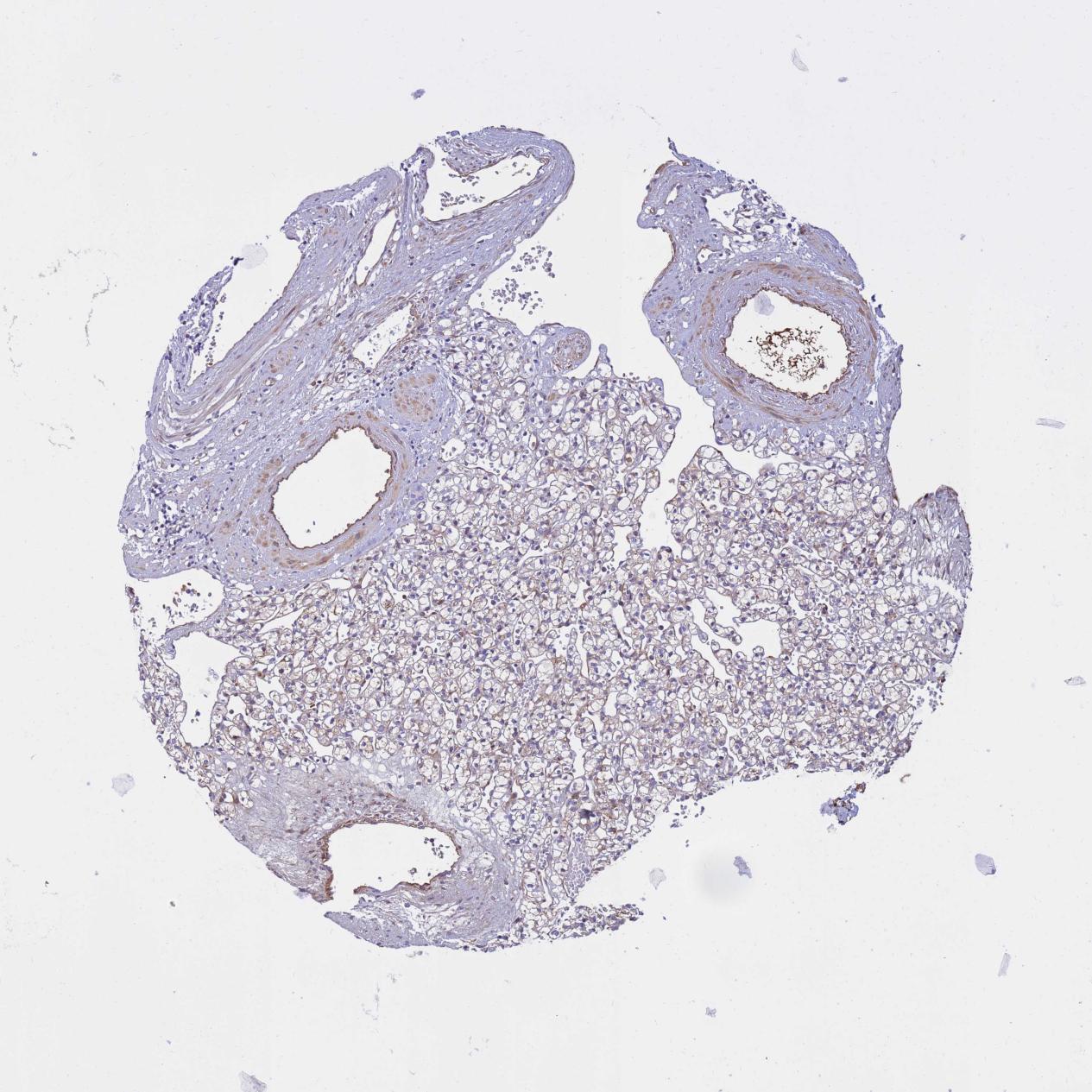

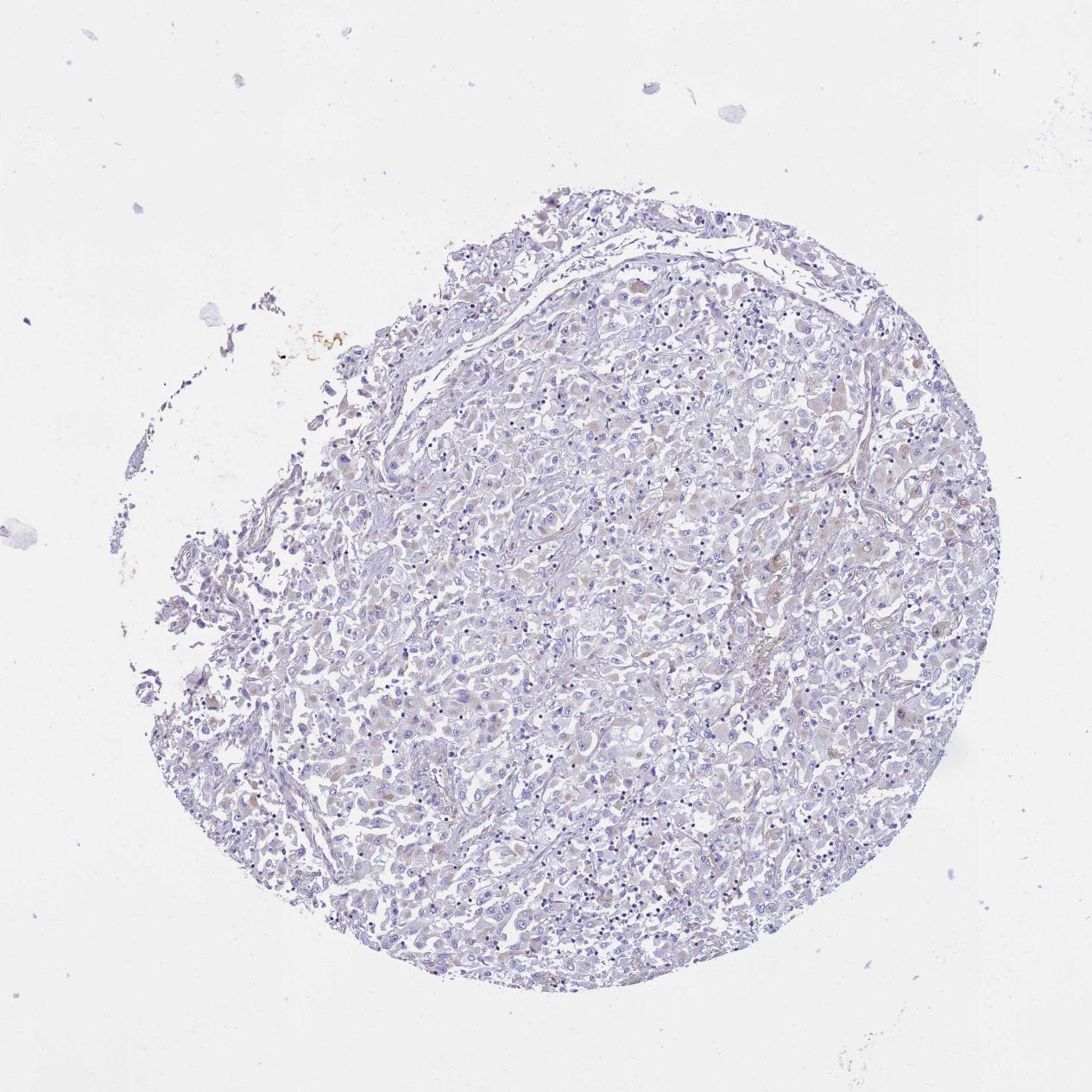

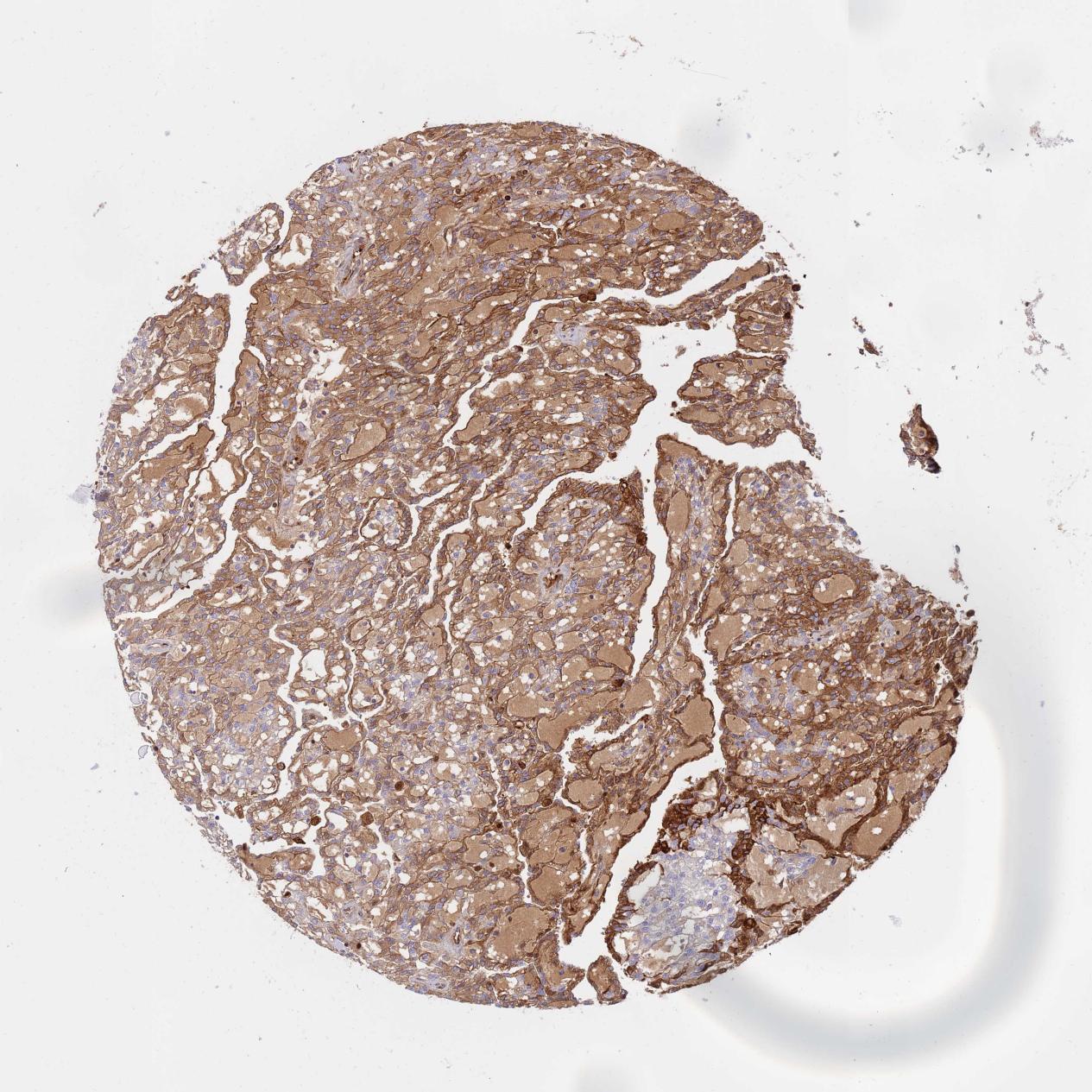


Figure 5G:

<https://www.proteinatlas.org/ENSG00000150054-MPP7/cancer/renal+cancer#cptac_renal_cell_carcinoma>


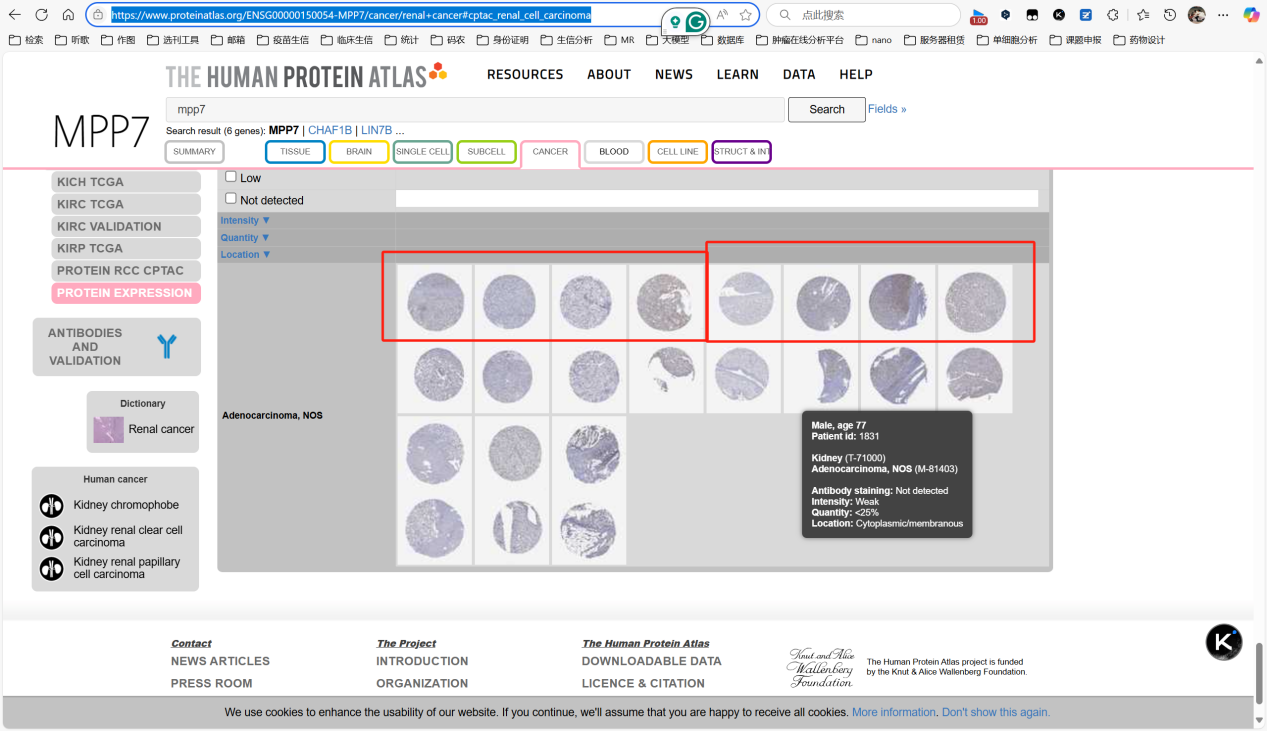


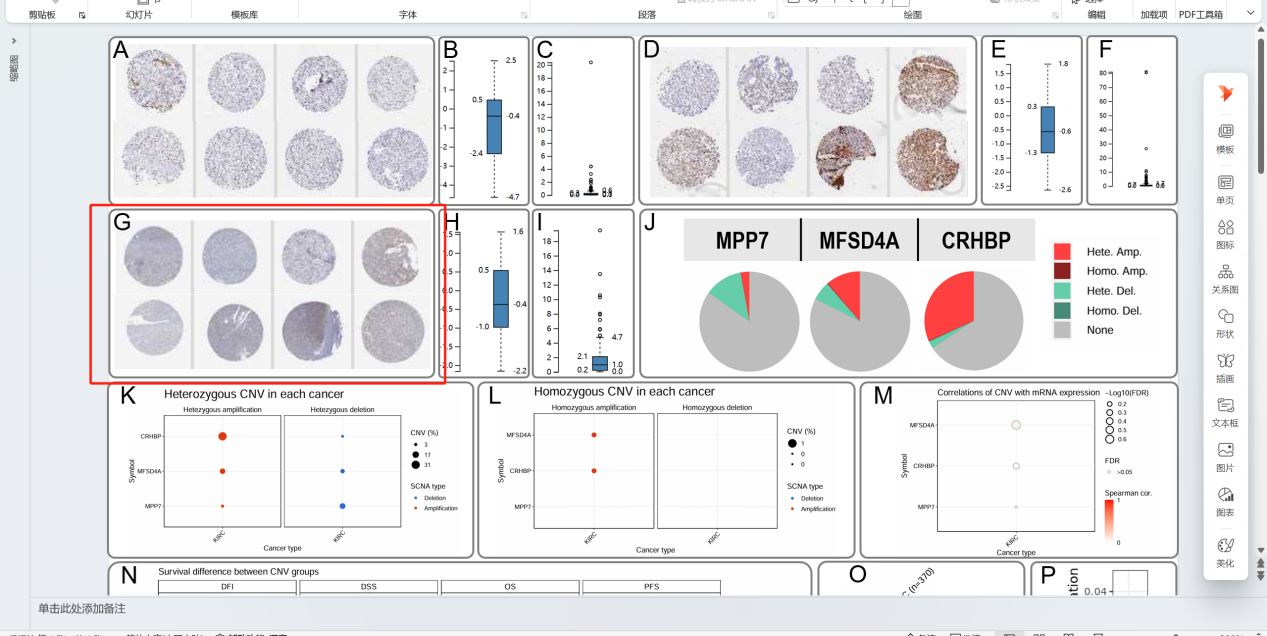


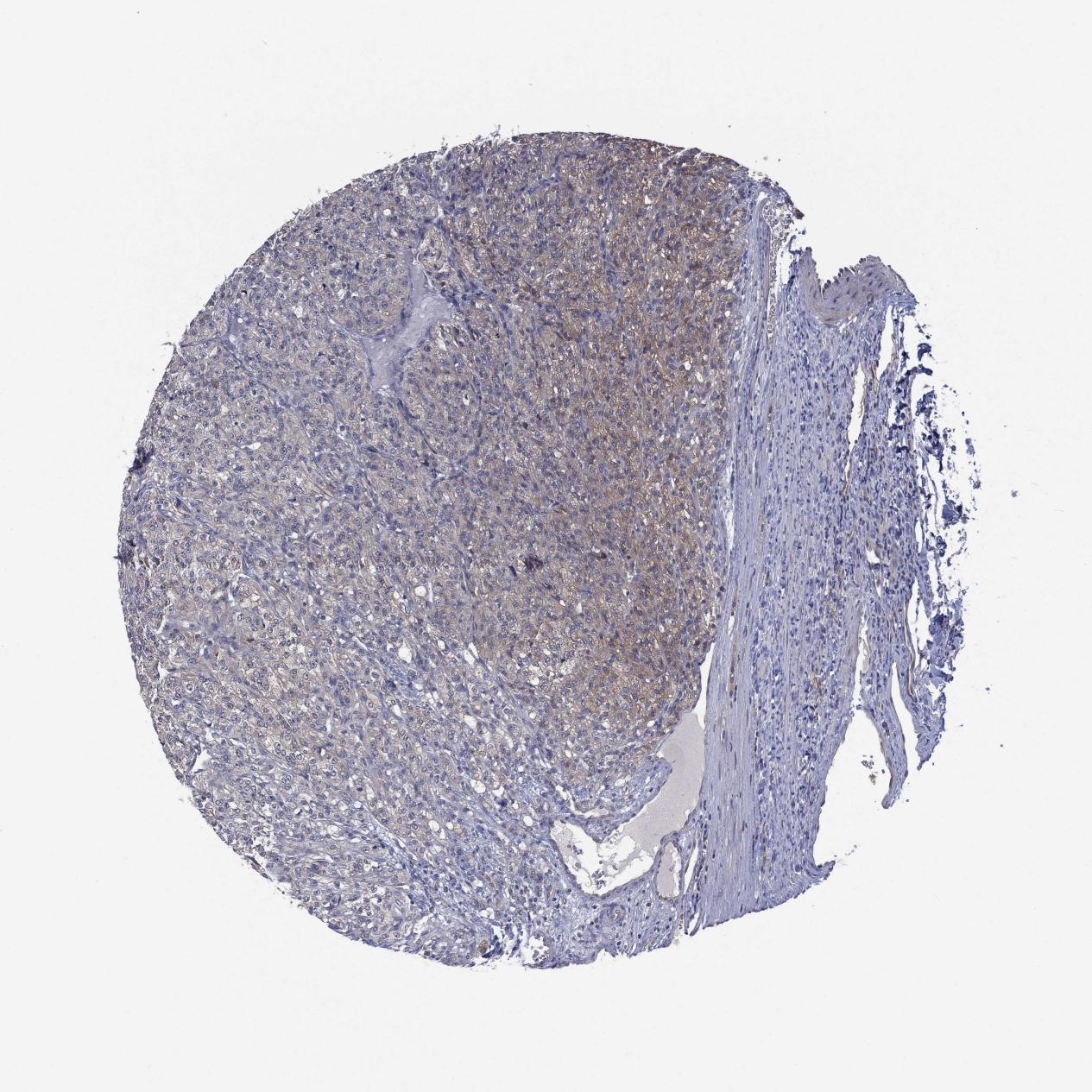

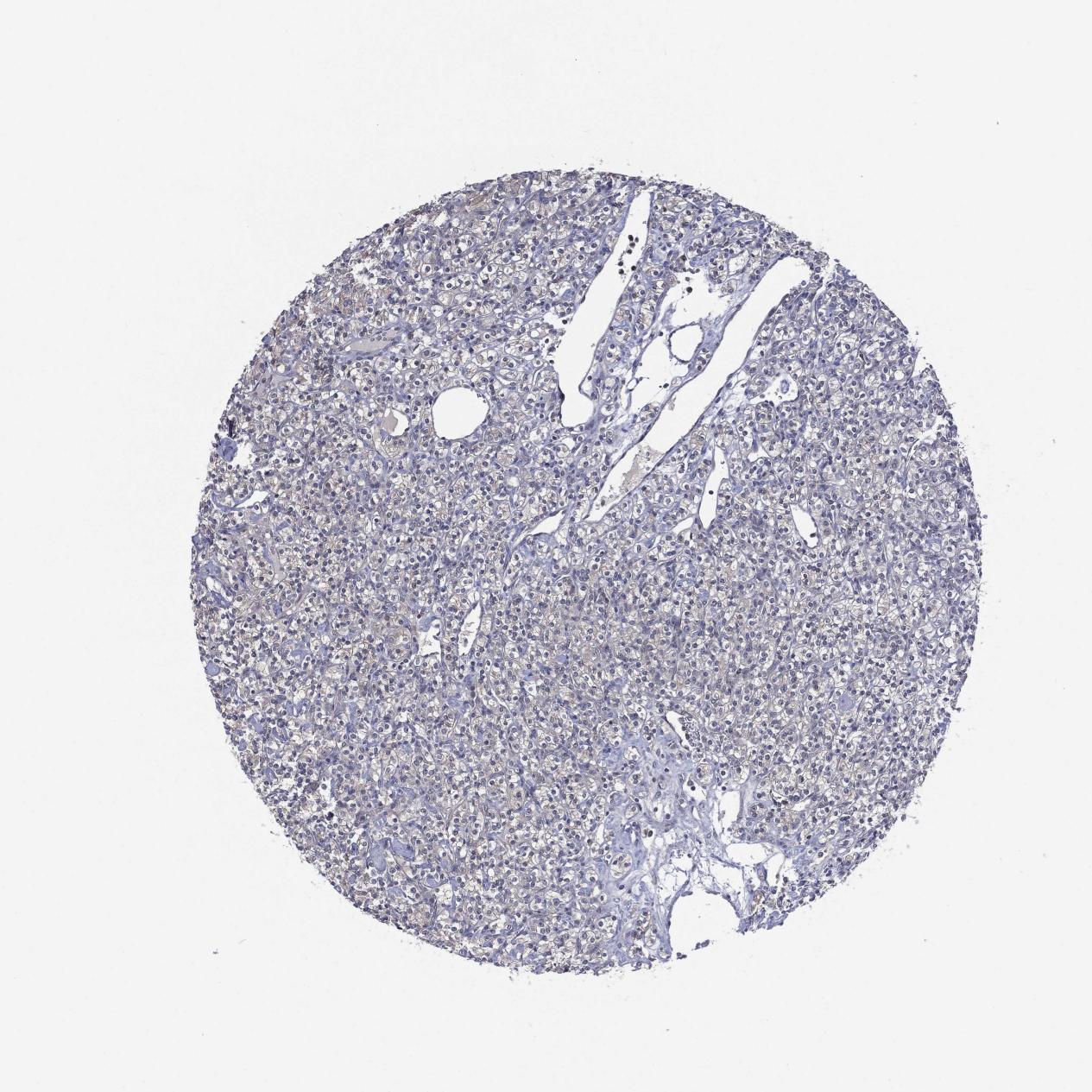

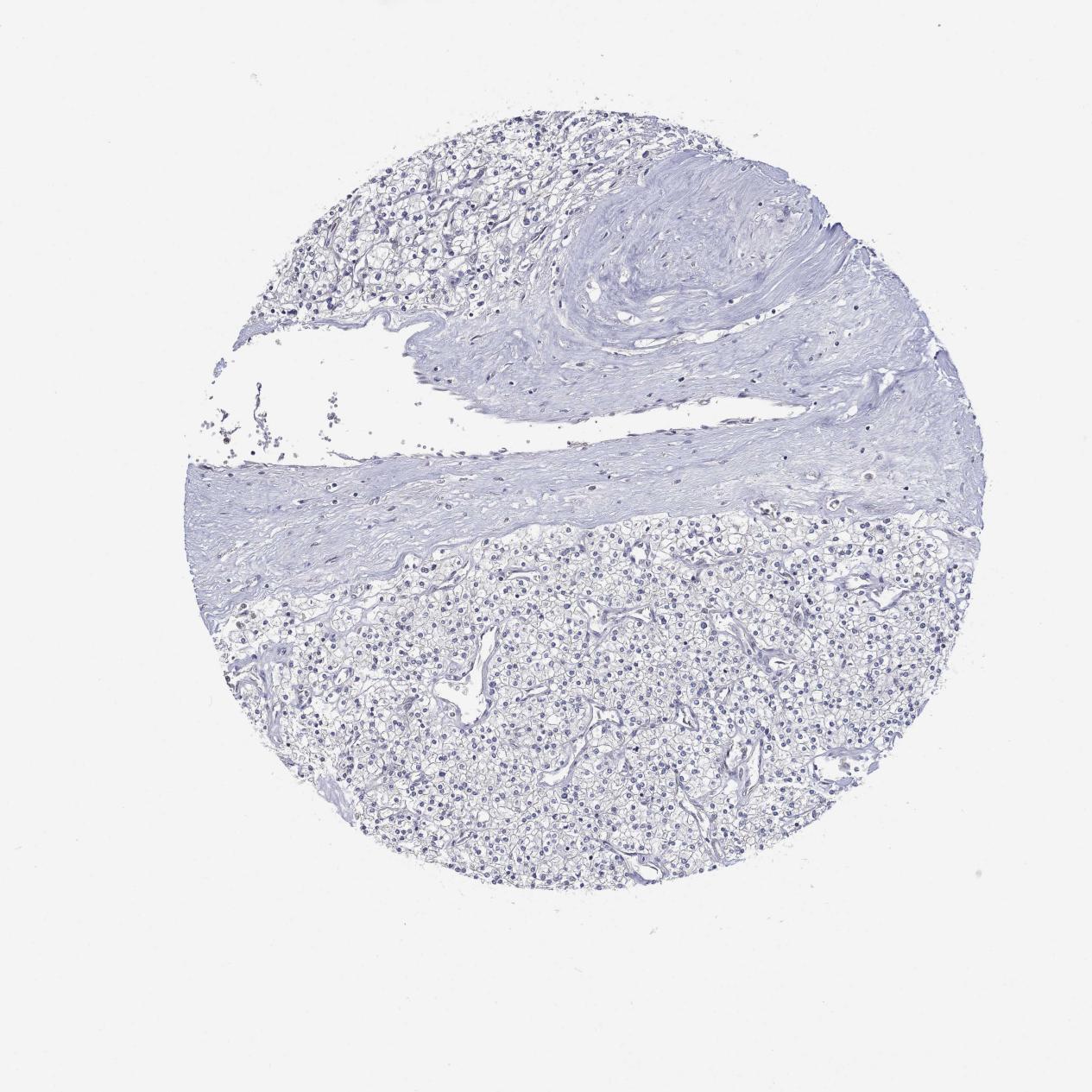

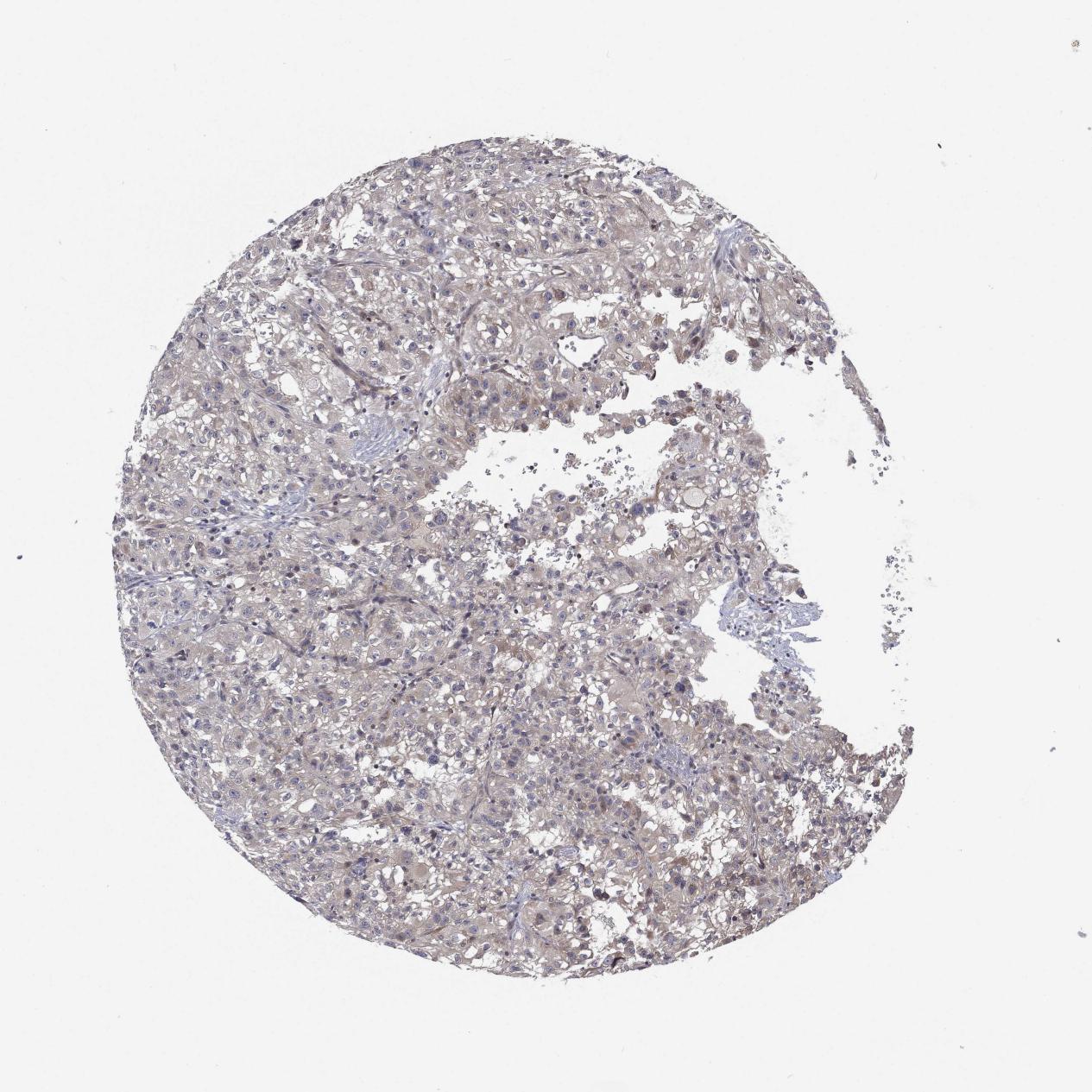

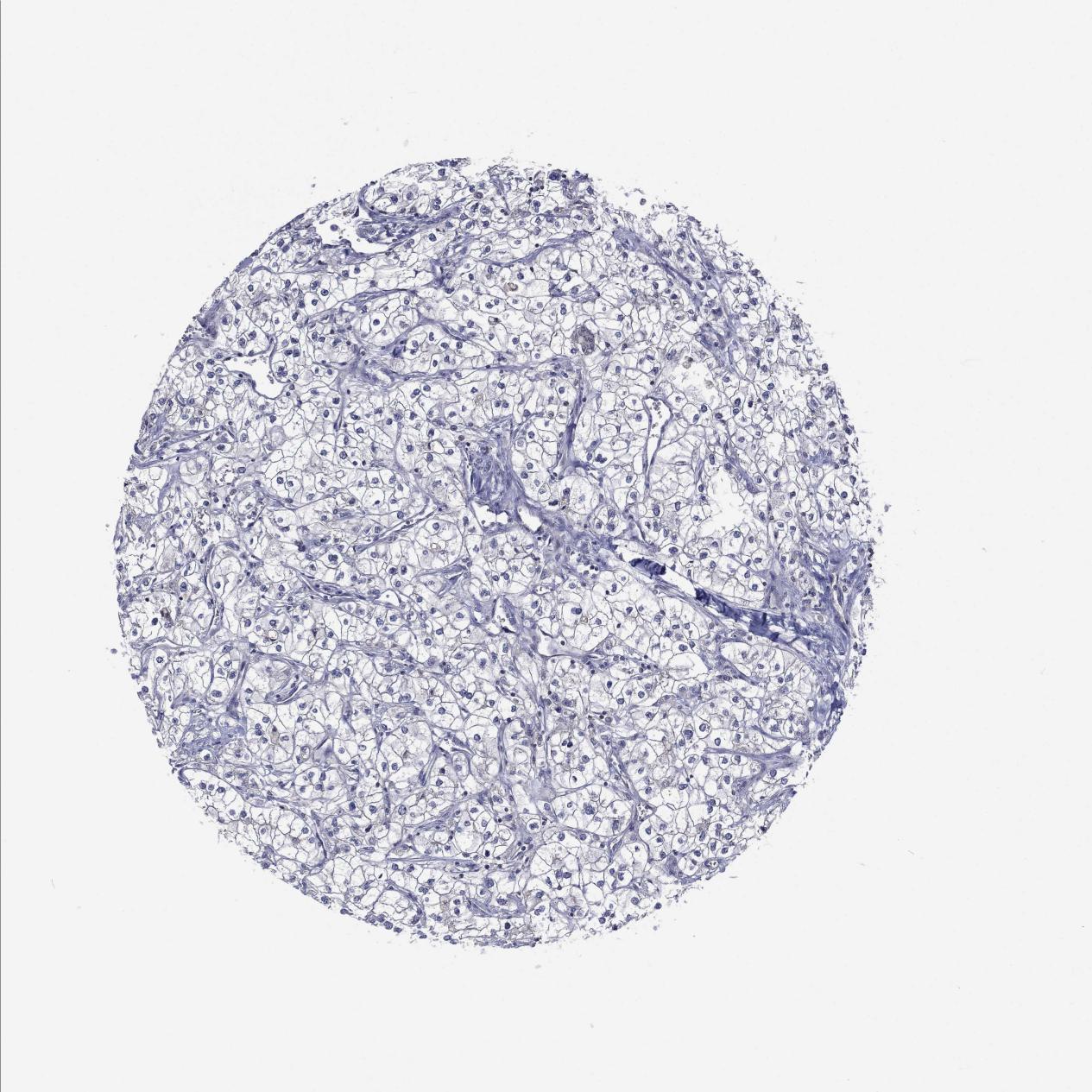

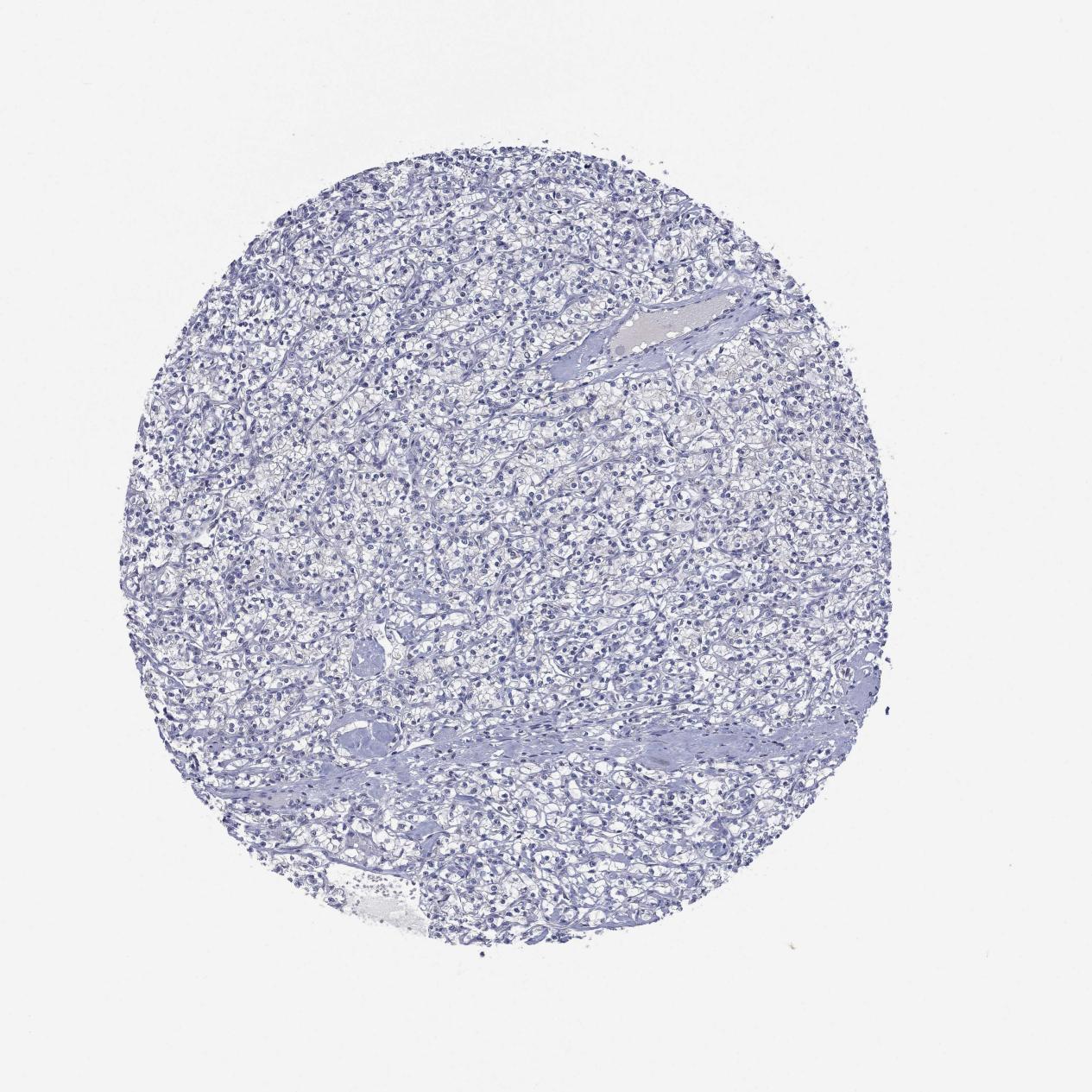

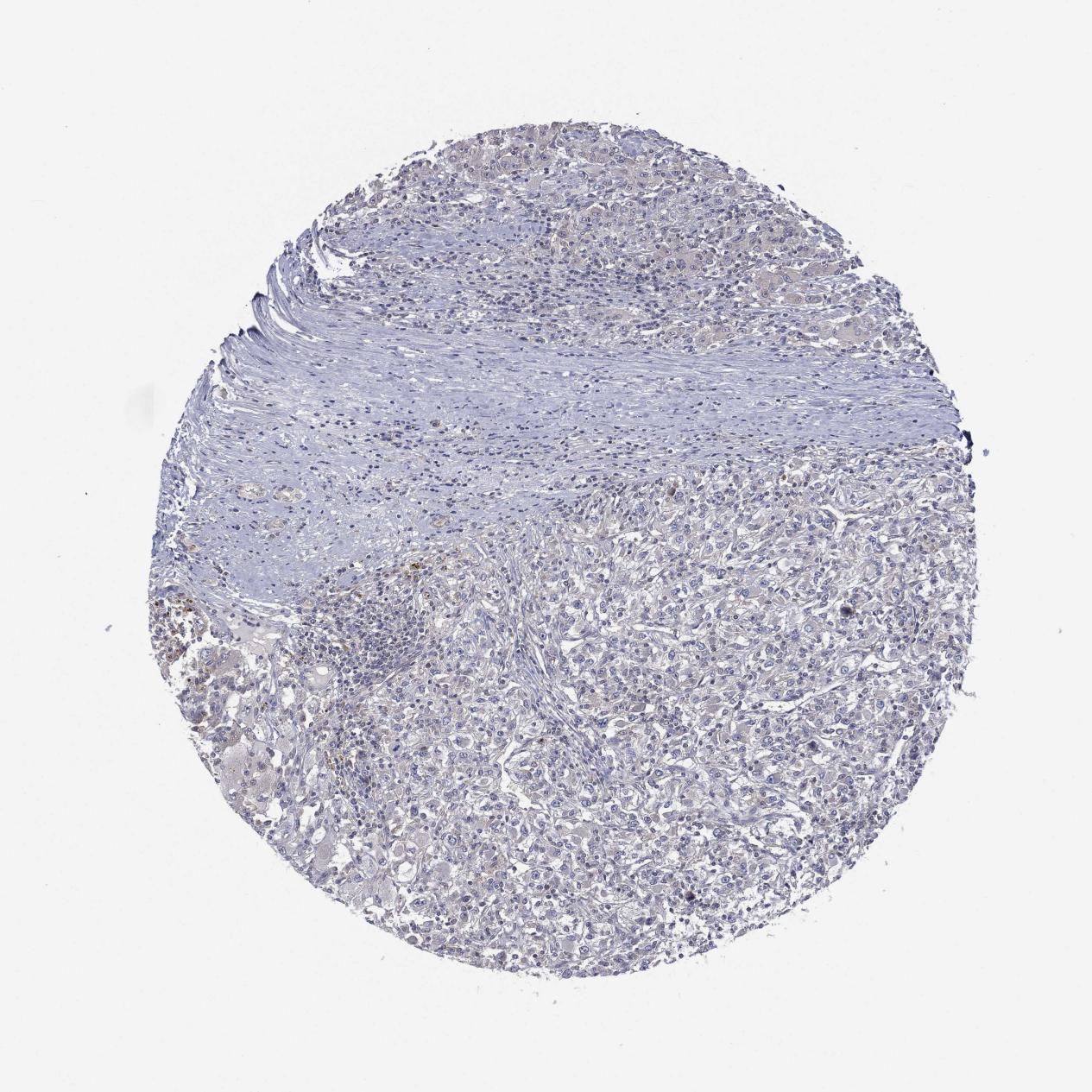

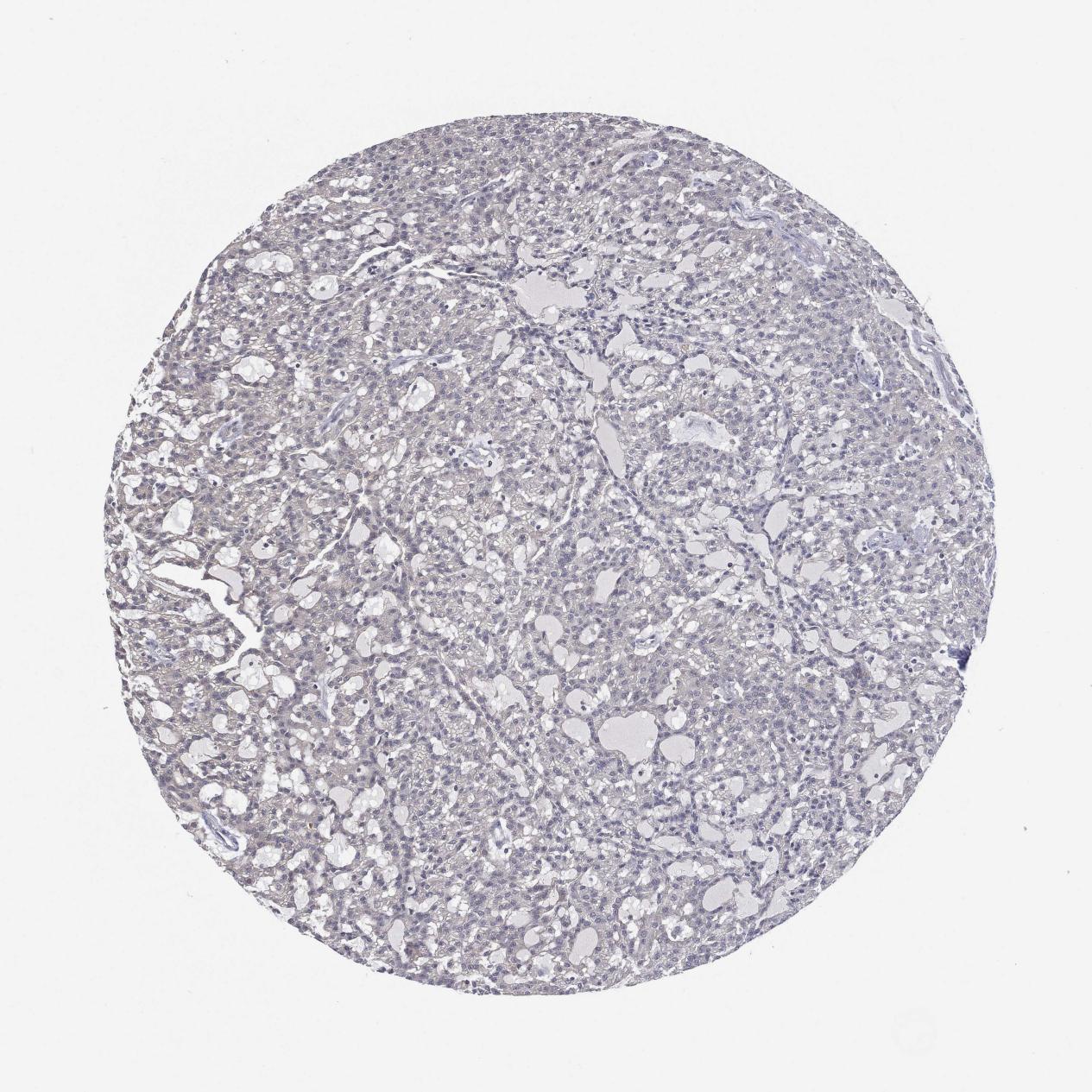

Supplement: Supplementary Material 2 — Original protein expression figure. [file DataSheet2.docx]
